# Multimodal deep-learning optimization of chiroptical properties in all-inorganic perovskite-coated TiO_2_ nanohelices and inverse-design transfer to organic chiral luminophores

**DOI:** 10.1038/s41467-026-74010-2

**Published:** 2026-06-04

**Authors:** Haifeng Sun, Yilun Zhang, Xiao Chen, Wentao Wang, Guang-Jie Xia, Zhifeng Huang

**Affiliations:** 1 Joint Institute of Advanced Materials and Green Energy Research, Great Bay University, Dongguan, Guangdong Province 523000 P. R. China; 2 Department of Chemistry, The Chinese University of Hong Kong, Shatin, N. T., Hong Kong SAR 999077 P. R. China; 3 Centre for Intelligent Computing, Great Bay Institute for Advanced Study, Dongguan, Guangdong Province 523000 P. R. China; 4 School of Automation, Chongqing University of Posts and Telecommunications, Chongqing, 400065 P. R. China; 5 School of Physical Sciences, Great Bay University, Dongguan, Guangdong Province 523000 P. R. China; 6 Shenzhen Research Institute, The Chinese University of Hong Kong, No. 10, 2nd Yuexing Road, Nanshan, Shenzhen, Guangdong Province 518057 P. R. China

**Keywords:** Nanoscale materials, Theory and computation

## Abstract

Circularly polarized luminescence (CPL) has been catching increasing attention for developing advanced photonic displays, quantum communication, bioimaging, and chiral sensing. All-inorganic chiral luminophores are superior to their organic or organic-inorganic hybrid counterparts in thermal stability, environmental robustness and device compatibility, but limited by the difficulty in fabrication and low luminescence dissymmetry factor (*g*_lum_ < 0.1), whereby *g*_lum_ is generally applied to evaluate the purity of circular polarization of CPL. Herein, chiral TiO_2_ nanohelices (NHs) act as chiral templates that are conformally coated with achiral perovskite luminophores composed of cesium lead bromides, to form all-inorganic chiral core@shell nano-luminophores. Chirality transmission from TiO_2_ NHs to perovskites accounts for the generation of CPL. Given by the complex and multifactorial experimental conditions, the manual engineering of fabrication procedure leads to an optimized *g*_lum_ = 0.2. To further optimize *g*_lum_, we develop OptiCPL, a few-shot multimodal deep-learning framework that integrates spectral and morphological features, to boost *g*_lum_ from 0.20 to 0.35 through model prediction and experimental validation. In addition, the OptiCPL model is transferrable to polymer F8BT-based chiral organic luminophores, achieving *g*_lum_ = 0.87. This work establishes a synergistic chiral core@shell approach and offers a transferable deep-learning framework for designing high-*g*_lum_ CPL materials.

## Abstract (web summary)

Manual optimization of materials with circularly polarized luminescence is time-consuming and labor-intensive. Here, the authors use multimodal deep learning to develop inorganic core-shell nanoluminophores with enhanced emission and extend this approach to an organic system.

## Introduction

Chiral subjects interact with right-handed (RH) and left-handed (LH) circularly polarized light in a differential manner, resulting in a diversity of chiroptical activities^1,2^. Investigating these chiroptical activities substantially promote potential development of a wide range of applications, such as three-dimensional display^3^, information storage^4^, encryption^5^, bioimaging^6^, chiral sensing^7^, and asymmetric photocatalysis^8^. As sunlight naturally exhibits little to no circular polarization, circular polarization is traditionally obtained by passing light through half- and quarter-waveplates^9^. However, this two-step modulation is limited by energy loss, attenuation of light intensity, and difficulty in miniaturizing optical devices^10^.

**Fig. 1:**
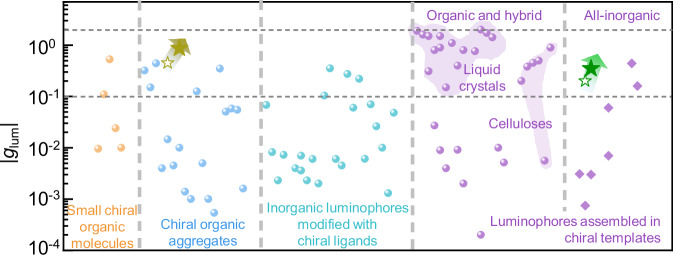
Summary of the absolute luminescence dissymmetric factors (|*g*_lum_|) of circularly polarized luminescence (CPL) in green generated from four kinds of chiral luminophores (refer to Supplementary Table 1). Luminophores assembled in liquid crystals and celluloses are highlighted by shadowed areas in rose. The all-inorganic chiral nano-luminophores (i.e., TiO_2_ NHs@CsPbBr_3_/Cs_4_PbBr_6_), as reported in this work, exhibit |*g*_lum_| = 0.2 (marked by hollow green star), increased to 0.35 (marked by solid green star) guided by OptiCPL, a universal few-shot multimodal deep-learning framework. The OptiCPL model can be transferred to organic chiral luminophores, leading to raising |*g*_lum_| of the polymer-based luminophores (*R*/*S*-5011/F8BT) from 0.50 (marked by hollow brown star) to 0.87 (marked by solid brown star). The data points within each category are separated along *x*-axis for visualization purposes. The horizontal dashed lines represent *g*_lum_ = 0.1 and 2, respectively. Data points are color-coded by material types: orange for small chiral organic molecules, blue for chiral organic aggregates, cyan for inorganic luminophores modified with chiral ligands, and purple for luminophores assembled in chiral templates, whereby purple diamonds denote all-inorganic ones. Source data are provided as a Source Data file.

To overcome these limitations, researchers have devised types of chiral luminophores capable of generating circularly polarized luminescence (CPL) under irradiative or electric stimulation^11,12^. Chiral luminophores include small chiral organic molecules^13^, chiral organic aggregates^14,15^, inorganic luminophores modified with chiral ligands (i.e., organic-inorganic hybrid luminophores)^16^, and luminophores embedded in chiral templates^17^. These CPL materials are typically characterized by the luminescence dissymmetry factor (*g*_lum_) indicating the purity of LH or RH circular polarization^18^, defined as^19^

1 $${g}_{{{\rm{lum}}}}=2({I}_{{{\rm{L}}}}-{I}_{{{\rm{R}}}})/({I}_{{{\rm{L}}}}+{I}_{{{\rm{R}}}})$$

where *I*_L_ and *I*_R_ are the emission intensities of LH- and RH-CPL, respectively. The maximum values of *g*_lum_ are +2 and –2, indicating exclusive generation of LH- and RH-CPL, respectively. The diverse types of chiral luminophores exhibit a wide range of *g*_lum_. For example, absolute values of *g*_lum_ (or |*g*_lum_|) of CPL in green are in a range of 10^−2^–10^−1^ for small chiral organic molecules, and 10^−4^–10^−1^ for chiral organic aggregates and hybrid chiral luminophores (Fig. 1, Supplementary Table 1). CPL with |*g*_lum_| <0.1 tends to severely limit its practical applications^20^, and less than one-fourth (11 out of 47 reports) of these organic or organic-inorganic hybrid luminophores possess |*g*_lum_| >0.1.

One way to increase |*g*_lum_| is to assemble luminophores in chiral templates, such as chiral metal-organic frameworks^21^, chiral polymers^22^, liquid crystals^17^, celluloses^23^, carbon dots^24^, and chiral inorganic nanomaterials^25,26^, due to chirality transfer from chiral templates to the assembled luminophores. However, most kinds of chiral templates result in |*g*_lum_| <0.1, except for organic liquid crystals and celluloses. Celluloses provide |*g*_lum_| <0.75^27^; but they are typically extracted from homochiral natural cellulosic sources, limiting the selection of generated CPL to either LH or RH circular polarization. Chiral liquid crystals serve as a promising chiral template^5,17,28^, reaching the theoretical maximum of |*g*_lum_| = 2.0^29^. Nevertheless, their use is limited by intricate synthesis and chiral resolution processes^30^, limited compatibility with luminophores^29^, complex control of co-assembly stability^31^, and vulnerability to external stimuli (e.g., temperature^32^ and electric fields^33^). It is desirable to develop alternative inorganic chiral templates with strong, engineerable chiroptical activity and structural robustness. More importantly, nearly all chiral luminophores exhibiting |*g*_lum_| >0.1 consist of either organic or organic-inorganic hybrid materials (Fig. 1), which generally lack thermal stability, environmental robustness, and device compatibility. To tackle these problems, CPL-based optoelectronic devices prefer all-inorganic chiral luminophores, but few of them show |*g*_lum_| >0.1 (marked as purple diamonds in Fig. 1). It is highly desired to enrich the material library of all-inorganic chiral luminophores with high |*g*_lum_|.

Generating CPL from guest luminophores embedded in chiral templates involves complicated fabrication processes, comprising the synthesis of luminophores, fabrication of chiral templates, and chiral host-guest assemblies^34^. Numerous experimental factors involved in these intricate processes interdependently influence the overall CPL performance. Such complexity presents challenges in the design and production of chiral host-guest assemblies that can yield high *g*_lum_. Therefore, systematic and guidance-oriented investigation is essentially required to elucidate fundamental correlations of multiple factors influencing *g*_lum_. Recently, deep learning and other artificial intelligence (AI) has been widely adopted in the fields of materials science, such as high-throughput screening of materials^35,36^, optimization of synthesis protocols and formulations^37^, high-fidelity modeling of material dynamics^38,39^, elucidation of material properties^40,41^, and inverse design^42^. By the same token, AI methodologies have been developed to accelerate the investigation of generating high-*g*_lum_ luminophores. For example, Dai et al. developed an “experiment–prediction–verification” framework to produce G-quartet-based CPL gels exhibiting *g*_lum_ = 0.15, whereby a decision-tree model was employed to correlate the synthesis conditions with *g*_lum_^34^. For circularly polarized phosphorescence, Liu et al. introduced a transfer learning strategy that integrated large language models with spectral embedding, leading to inverse design of materials yielding |*g*_lum_| >1.8^43^. The transfer learning framework gave rise to narrow emission bandwidth and customized chiroptical performance, based on the limited experimental data. These pioneering efforts highlight the potential of AI in guiding the discovery of alternative materials generating high-quality CPL. However, several critical challenges, including data sparsity, limited multimodal integration, and poor model generalization^34,43^, remain unsolved in AI-directed fabrications, limiting the development of all-inorganic high-*g*_lum_ CPL materials.

**Fig. 2:**
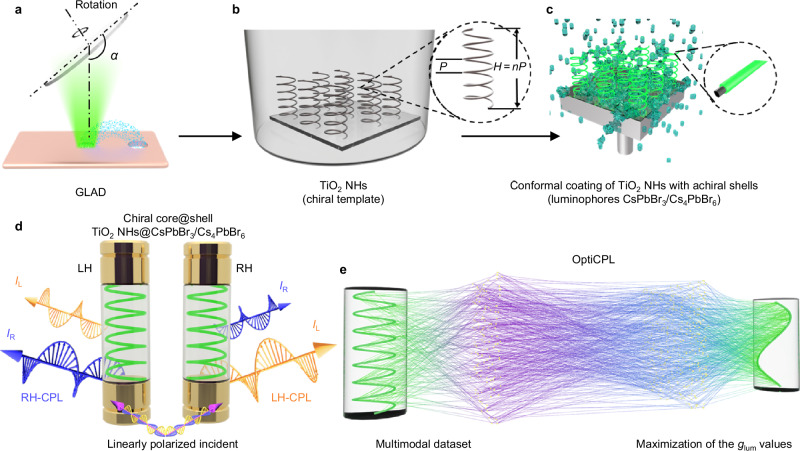
Schematic pipeline of fabrication, chiroptical characterization, and deep learning-enabled chiroptical optimization of all-inorganic chiral core@shell nano-luminophores. **a** Glancing angle deposition (at an angle $\alpha$) of titanium dioxide (TiO_2_) with unidirectional substrate rotation, for **b** generating TiO_2_ nanohelices (NHs) with the helicity characterized with the handedness, helical pitch (*P*), the number of pitches (*n*), and helical height (*H* = *nP*). **c** TiO_2_ NHs, functioning as chiral templates, are conformally coated with achiral luminophores to form chiral core@shell nano-luminophores (i.e., TiO_2_ NHs@CsPbBr_3_/Cs_4_PbBr_6_). **d** Under linearly polarized excitation, left-handed (LH) and right-handed (RH) TiO_2_ NHs@CsPbBr_3_/Cs_4_PbBr_6_ generate green CPL preferentially polarized in RH and LH, respectively. **e** Multimodal dataset including the experimental conditions of forming chiral core@shells, as well as structural images and optical characterizations, are used to train the deep-learning model, OptiCPL, which is applied to accelerate the optimization of |*g*_lum_|.

Herein, inorganic nanohelices (NHs), fabricated by glancing angle deposition (GLAD, Fig. 2a, b)^44^, function as chiral templates on which achiral inorganic luminophores composed of cesium lead bromides (a mixture of CsPbBr_3_ and Cs_4_PbBr_6_) are conformally coated (Fig. 2c). This leads to forming all-inorganic chiral core@shell nano-luminophores (i.e., NHs@CsPbBr_3_/Cs_4_PbBr_6_) for photoinduced generation of CPL in green (Fig. 2d), owing to chirality transfer from the chiral cores to achiral luminophores. Inorganic NHs are made of titanium dioxide (TiO_2_), commonly used as electron-transporting layers in optoelectronic devices^45–47^. TiO_2_ NHs tend to exhibit chemical stability while assembling with hybrid and all-inorganic perovskites, possess structural robustness under external stimulations^48^, and enhance photoluminescence (PL) stability of cesium lead halides^49^. GLAD is a versatile, scalable technique^50,51^ capable of producing inorganic NHs^52^ in large area^53^, favorable for mass production of optoelectronic devices. More importantly, GLAD enables flexible engineering of the helicity (including the handedness, helical pitch *P*, and the number of pitches *n*, Fig. 2b)^54,55^, which plays an essential role in determining chiroptical activities of inorganic NHs possessing *P* comparable to the excitation wavelength^56^. For instance, we found that elongating *P* of luminophore NHs made of cadmium selenide (CdSe) from 150 to ≈550 nm resulted in the maximization of chiroptical activities characterized with circular dichroism (CD) and 40–fold amplification of *g*_lum_ of CPL in red, whereby CdSe NHs exhibited *g*_lum_ of 0.15 at *P* = 570 nm^56^. Cesium lead bromides are selected for generating green CPL, owing to high photoluminescence quantum yield (PLQY, as favored for producing intensive CPL)^57^, excellent solution processability^58^, and compatibility with TiO_2_^49^. The chiral core@shell formation comprises multiple-step, complicated processes, making the optimization of *g*_lum_ tedious and time-consuming. To address this challenge, we introduce OptiCPL (Fig. 2e), a few-shot multimodal deep-learning framework to achieve 75% enhancement of *g*_lum_ (from 0.20 to 0.35). OptiCPL is transferable to polymer (F8BT)-based chiral organic luminophores, leading to a 74% enhancement of *g*_lum_ (from 0.50 to 0.87). This work not only provides a solution for generating high–*g*_lum_ CPL from all-inorganic chiral luminophores, but also devises a versatile deep-learning framework for accelerating inverse design of high–*g*_lum_ luminophores.

## Results and discussion

### Fabrication of all-inorganic nano-luminophores

**Fig. 3:**
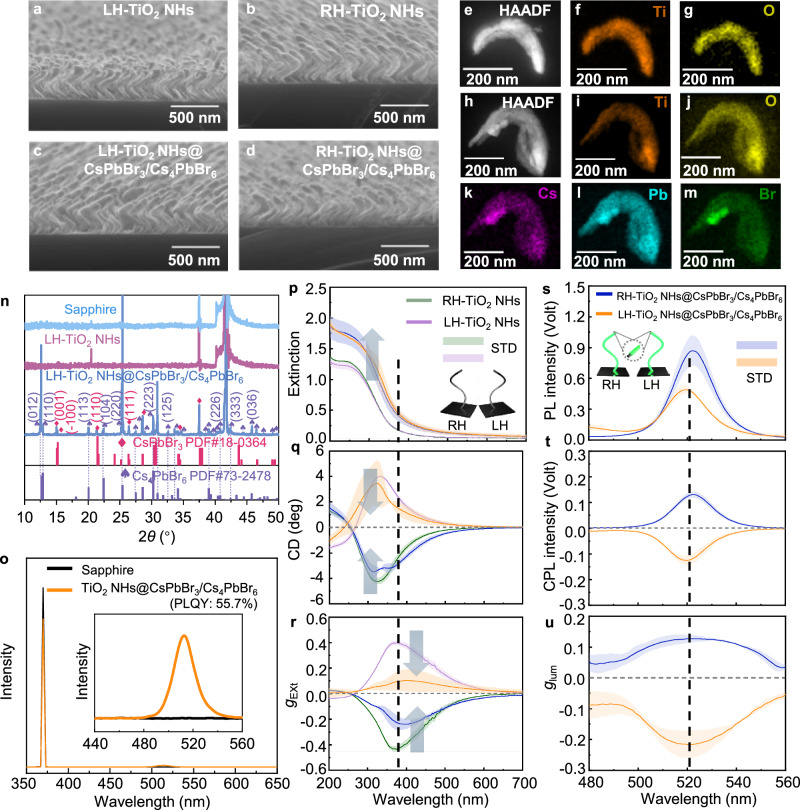
Fabrication of chiral core@shell nano-luminophores consisting of TiO₂ NHs@CsPbBr_3_/Cs_4_PbBr_6_. The core NHs (*P* ≈ 300 nm and *n* = 1), deposited by GLAD, was conformally coated with achiral shells via ligand-assisted co-precipitation (at the concentration of perovskite precursors [CsBr] = [PbBr_2_] = 4 mmol L^-1^, and soaking period (*t*_s_) = 5 h). Scanning electron microscopies (SEM) tilted images: **a** LH- and **b** RH-TiO_2_ NHs (*P* = 310 nm and 300 nm, respectively); **c** LH- an**d d** RH-TiO_2_ NHs@CsPbBr_3_/Cs_4_PbBr_6_ (*P* = 315 nm and 310 nm, respectively). **e** High-angle annular dark-field scanning transmission electron microscopy (HAADF-STEM) image with elemental mapping of LH-TiO_2_ NHs: **f** Ti; **g** O. **h** HAADF-STEM image with elemental mapping of LH-TiO_2_ NHs@CsPbBr_3_/Cs_4_PbBr_6_: **i** Ti; **j** O; **k** Cs; **l** Pb; **m** Br. **n** X-ray diffraction (XRD) spectra of sapphire on which chiral nano-luminophores were deposited, LH-TiO_2_ NHs, LH-TiO_2_ NHs@CsPbBr_3_/Cs_4_PbBr_6_, together with XRD patterns of CsPbBr_3_ (PDF#18-0364) and Cs_4_PbBr_6_ (PDF#73-2478). **o** Measurement of photoluminescence quantum yield (PLQY) of LH-TiO_2_ NHs@CsPbBr_3_/Cs_4_PbBr_6_. **p–r** UV–visible spectra of TiO_2_ NHs and TiO_2_ NHs@CsPbBr_3_/Cs_4_PbBr_6_: **p** extinction, **q** circular dichroism (CD), and **r** anisotropic *g*-factor (*g*_Ext_). Luminescence characterization of TiO_2_ NHs@CsPbBr_3_/Cs_4_PbBr_6_: **s** photoluminescence (PL), **t** circularly polarized luminescence (CPL), and **u** luminescence dissymmetry factor (*g*_lum_). (p-u) Solid lines: mean values; shaded areas: standard deviations (STD); statistically evaluated from multiple measurements. The shell coating enhances **p** extinction while suppressing **q** CD and **r** *g*_Ext_ signals (marked by arrows). **p–r** RH-cores: green spectra; LH-cores: magenta spectra. **p–u** RH-core@shells: blue spectra; LH-core@shells: orange spectra. Source data are provided as a Source Data file.

Forming chiral core@shell nano-luminophores comprises two sequential steps, i.e., GLAD-based fabrication of TiO_2_ NHs (chiral cores) followed by conformal coating with cesium lead bromides. A close-packed array of TiO_2_ NHs (Supplementary Fig. 1) were deposited on a supporting substrate via GLAD of TiO_2_ at a deposition angle (*α*, Fig. 2a) of 87°^59^. During GLAD, unidirectional substrate rotation in counterclockwise and clockwise led to sculpting TiO_2_ NHs in LH and RH, respectively (Fig. 3a, b, Supplementary Fig. 2a, b)^55^. Besides engineering handedness, GLAD enabled adjustment of the helical pitch (*P*, Eq. (4)) and the number of *P* (*n*, equal to the number of substrate-rotation circles) (Fig. 2b)^60^. For example, TiO_2_ NHs (with *P* ≈ 300 nm and *n* = 1) exhibit branched, rough surfaces (Fig. 3e) and are amorphous (claret spectrum, Fig. 3n).

Then TiO_2_ NHs were conformally coated with cesium lead bromides through ligand-assisted co-precipitation (under the conditions of, e.g., the concentration of perovskite precursors [CsBr] = [PbBr_2_] = 4 mmol L^-1^, and soaking period (*t*_s_) of 5 h; see Methods), leading to the formation of chiral core@shell nanostructures (Fig. 3c, d, Supplementary Fig. 2c, d). The shells, with the thickness of 10–20 nm (Fig. 3e-g versus 3h-m), were made from a mixture of CsPbBr_3_ and Cs_4_PbBr_6_ (Fig. 3n), and thus the chiral core@shell nano-luminophores are denoted as TiO_2_ NHs@CsPbBr_3_/Cs_4_PbBr_6_. The formation of core@shell heterojunctions was further confirmed by observing the shift of binding energy of all the elements comprising the cores and shells (Supplementary Fig. 1), as a result of charge transfer between them.

### Photoinduced generation of CPL in green

Functioning as chiral templates, TiO_2_ NHs are expected to possess chiroptical activity transferable to achiral luminophore shells. Chiroptical activity can be typically characterized by CD, denoting differential absorption/extinction of LH and RH circularly polarized light by a chiral substance^61^. A close-packed array of TiO_2_ NHs exhibited significant extinction (including absorption, reflection, scattering and light trapping in the array)^62^ in the ultraviolet (UV) region (Fig. 3p), where the array resonantly showed bisignate CD signals (Supplementary Figs. 3, 4). Switching the helical handedness from LH to RH made the CD spectrum flip around the zero-CD axis (Fig. 3q), illustrating that the TiO_2_ NHs intrinsically possess chiroptical activity due to their structural helicity. The anisotropic growth of NH arrays will introduce linear birefringence and linear dichroism that may mask their CD signals. To eliminate these linear disturbances, eight CD spectra of a given NH array were recorded as a function of the polar angle (*φ*) and azimuth angle (*γ*), and then algebraically averaged (Supplementary Figs. 3, 4; see Methods)^63^. To evaluate the chiroptical activity of individual NHs in an array, anisotropic *g*-factor (*g*_Ext_) was calculated, given by ref. ^64^

2 $${g}_{{{\rm{Ext}}}}=\frac{{{\rm{CD}}}}{16500{{\rm{Ext}}}}$$

where CD and Ext represent the ellipticity (in units: mdeg) and the extinction (excited by linearly polarized light), respectively. The TiO_2_ NHs (*P* ≈ 300 nm and *n* = 1) showed a mean absolute *g*_Ext_ (or |*g*_Ext_| ) of 0.4 at a wavelength of 370 nm (Fig. 3r), underscoring their strong intrinsic chiroptical activity, given that |*g*_Ext_| has the theoretical maximum value of 2.

Conformally coating the chiral cores with CsPbBr_3_/Cs_4_PbBr_6_ resulted in enhancing extinction mainly in the UV region (marked by arrow, Fig. 3p) and weakening their chiroptical activity (marked by arrows, Fig. 3q, r, Supplementary Figs. 5, 6). The suppression of their chiroptical activity is probably ascribed to the shell-induced change of dielectric permittivity of the medium surrounding the TiO_2_ NHs^65–67^. The chiroptical activity of TiO_2_ NHs is governed not only by their helicity, but also by the local electromagnetic field and optical chirality in the vicinity of TiO_2_ NHs^67^. Compared to the surrounding air medium of the chiral cores, the shell coating leads to an increase in the medium dielectric permittivity, reducing the electromagnetic field gradients near the chiral cores, suppressing the local density of optical chirality, and thus decreasing the chiroptical activity of TiO_2_ NHs^65,66^.

Under the optimized photo-excitation conditions (i.e., excitation wavelength = 370 nm and *γ* = 30°, Supplementary Fig. 7), the TiO_2_ NHs@CsPbBr_3_/Cs_4_PbBr_6_ generated PL at a wavelength of ≈ 520 nm (Fig. 3s), with PLQY = 55.7% (Fig. 3o). On resonant with PL, the chiral nano-luminophores generated green CPL. LH- and RH-TiO_2_ NHs@CsPbBr_3_/Cs_4_PbBr_6_ preferentially generated RH- and LH-CPL with an average *g*_lum_ of −0.2 and 0.13 (at the wavelength of 520 nm), respectively (Fig. 3t,u, Supplementary Figs. 8, 9). |*g*_lum_| of the LH-core@shells was not equal to that of RH-core@shells, possibly ascribed to gear backlash-induced asymmetric substrate rotation in clockwise and counterclockwise during GLAD. Analogous to the measurement of CD spectra, multiple CPL spectra of a core@shell sample were monitored at *φ* = 0°, 45°, 90°, 135° and *γ* = 30°, and then algebraically averaged for eliminating the linear disturbance (Supplementary Figs. 7–9).

In the absence of the NH cores, the as-precipitated CsPbBr_3_/Cs_4_PbBr_6_ generated strong PL at 520 nm under 370-nm irradiative excitation, without chiroptical activity characterized by CD or CPL (Supplementary Fig. 10). It illuminates that chiroptical activities of the achiral CsPbBr_3_/Cs_4_PbBr_6_ shells originate from chirality transfer from the helicity of NH cores.

### Mechanisms of CPL generation

As intuitively understood, CPL of the luminous shells made of achiral all-inorganic perovskites originates from chirality transfer from the NH cores to the shells, whereby the shells duplicate the TiO_2_ helicity via conformal coating. To elucidate the core-to-shell chirality transfer, the microstructures of shells were characterized. The shells are polycrystalline (Fig. 3n) with a mean grain size of ≈ 10 nm (Supplementary Fig. 11). This indicates that the shells are composed of achiral single-crystal perovskite nano-domains, which helically assemble through connections with amorphous perovskite grain boundaries to duplicate the helicity of NH cores.

CPL may result from the scattering of green PL (generated from achiral nanocrystals in the shells) in the close-packed core@shell array. However, the TiO_2_ NHs@CsPbBr_3_/Cs_4_PbBr_6_ array exhibited negligible extinction in the visible region, especially at wavelengths > 450 nm (Fig. 3p). This indicates that the core@shell array is transparent to green PL, and thus CPL cannot stem from the array-induced scattering of green PL. Another possible mechanism is the collective coupling of PL from these helically assembled CsPbBr_3_/Cs_4_PbBr_6_ nano-domains, and the CPL color corresponds to the PL color of the CsPbBr_3_/Cs_4_PbBr_6_ nano-domains (Supplementary Fig. 10c)^25,26^. Such collective coupling possibly realizes the core-to-shell chirality transmission for generating CPL.

### Manual engineering of *g*_lum_

The core@shell formation consists of the GLAD-based fabrication of NH cores and shell coating, and thus a set of experimental conditions of these two fabrication processes have essential effects on *g*_lum_. In the GLAD process, the packing density of NHs in an array is mainly determined by *α*^68^, while deposition rate (*R*_d_) influences the coil diameter of individual NHs^52^. The NH density and coin diameter will significantly affect the diffusion of perovskite precursors (CsBr and PbBr_2_) in the NH arrays, governing the conformal shell coating. The helicity of NH cores, including handedness, *P* and *n*, has been found to determine *g*_Ext, NHs_ (*g*_Ext_ of individual NHs in an array)^69^, which will influence *g*_lum_ due to the core-to-shell chirality transfer. In the shell coating, [CsBr], [PbBr_2_], and *t*_s_ play critical roles in forming conformal coatings of perovskites.

**Fig. 4:**
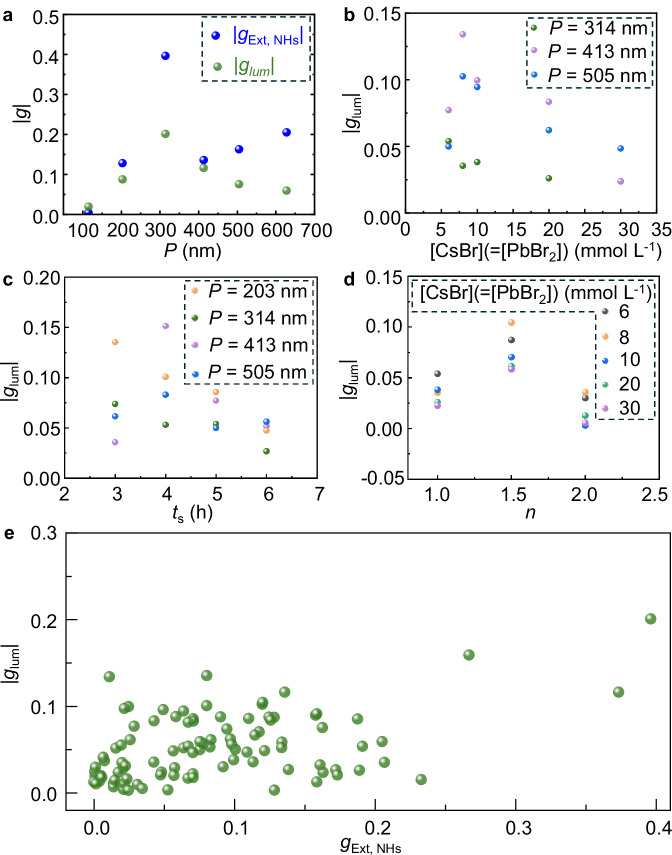
Manual engineering of *g*_lum_ of LH-TiO_2_ NHs@CsPbBr_3_/Cs_4_PbBr_6_. **a** Plots of *g*_Ext_ of LH-TiO_2_ NHs (*g*_Ext, NHs_, measured at a wavelength of 370 nm, blue dots) and the absolute *g*_lum_ (|*g*_lum_| , evaluated at a wavelength of 520 nm, green dots) versus *P* ( = 100–650 nm) of LH-TiO_2_ NHs, at *n* = 1, [CsBr] = [PbBr_2_] = 4 mmol L^-1^, and *t*_s_ = 5 h. **b** Plots of |*g*_lum_| versus [CsBr] ( = [PbBr_2_] = 6–30 mmol L^-1^), at *P* = 300–505 nm, *n* = 1, and *t*_s_ = 5 h. **c** Plots of |*g*_lum_| versus *t*_s_ (= 3–6 h), at *P* = 200–505 nm, *n* = 1, [CsBr] = [PbBr_2_] = 6 mmol L^−1^. **d** Plots of |*g*_lum_| versus *n* (= 1, 1.5, 2), at *P* = 314 nm, [CsBr] = [PbBr_2_] = 6–30 mmol L^-1^, and *t*_s_ = 5 h. In (a-d), GLAD of NH cores were performed at *α* = 87°, deposition rate of TiO_2_ (*R*_d_) = 0.15 nm s^-1^, and the calibration factor of *R*_d_ at *α* (i.e., *β*) = 0.33. **e** Plots of |*g*_lum_| versus *g*_Ext, NHs_, at *α* = 85°–87°, *β* = 0.33-0.4, *R*_d_ = 0.15–0.25 nm s^-1^, *P* = 100–650 nm, *n* = 1–8, [CsBr] = [PbBr_2_] = 4–30 mmol L^-1^, and *t*_s_ = 3–6 h, where there are 98 data points in total.

Conventionally, we optimized *g*_lum_ through systematic, tedious tuning of the abovementioned parameters. First of all, GLAD was performed at *α* varying in a range of 86.5°–89°, and the fabrication of TiO_2_ NHs was optimized at *α* = 87° (Supplementary Fig. 12). The NHs could hardly be formed at *α* > 88.5°^70^. *α* < 87° made the NH arrays too close-packed to facilitate the conformal shell-coating, while the loose-packed NH arrays deposited at *α* > 87° exhibited weak PL and CPL emission. *R*_d_ = 0.15 nm s^-1^ was preferred for generating thin NHs (Supplementary Fig. 13)^53^, favored for the diffusion-induced conformal shell-coating. Then at *α* = 87° and *R*_d_ = 0.15 nm s^-1^, for example, elongating *P* from 100 to 650 nm (Supplementary Figs. 14-19) made |*g*_lum_| show a peak at *P* = 300 nm, under the conditions of [CsBr] = [PbBr_2_] = 4 mmol L^-1^ and *t*_s_ = 5 h (Fig. 4a, Supplementary Fig. 20). The core NHs tend to increase their surface area when *P* rises. At small [CsBr] and [PbBr_2_] ( = 4 mmol L^-1^), the conformal shell-coating is favorable at *P* = 300 nm; *P* > 300 nm induces insufficient shell coating, making |*g*_lum_| shrink. The *P*-induced variation of *g*_Ext, NHs_ was analogous to that of |*g*_lum_| (Fig. 4a), owing to chirality transfer from the chiral cores to achiral shells.

Given that [CsBr] = [PbBr_2_] = 4 mmol L^-1^ may be too small to generate high *g*_lum_, [CsBr] ( = [PbBr_2_]) was adjusted in a range of 6–30 mmol L^-1^. Increasing [CsBr] made |*g*_lum_| continuously shrink at *P* of 314 nm but reach a peak at [CsBr] = 8 mmol L^-1^ and then decrease at *P* = 413 and 505 nm (Fig. 4b and Supplementary Figs. 21–23). When the one-turn TiO_2_ NHs are short (*P* = 314 nm), perovskite precursors ([CsBr] = [PbBr_2_] > 5 mmol L^-1^) are in excess to deteriorate chirality transfer from the NH cores, resulting in the suppression of |*g*_lum_| . The elongation of *P* (i.e., *P* > 400 nm) caused the excess of perovskite precursors to occur at [CsBr] > 8 mmol L^-1^, so that |*g*_lum_| reached a peak at 8 mmol L^-1^. Therefore, [CsBr] = [PbBr_2_] = 6 mmol L^-1^ was selected to facilitate chirality transfer and study the influence of *t*_s_ (varying in a range of 3–6 h) on |*g*_lum_| . With elongating *t*_s_, |*g*_lum_| continuously decreased from 0.13 to 0.05 at *P* of 203 nm and from 0.07 to 0.03 at *P* of 314 nm, but reached a peak at *t*_s_ of 4 h when *P* > 400 nm (Fig. 4c and Supplementary Fig. 24). It is indicated that a moderate *t*_s_ (= 4–5 h) is generally preferred. Furthermore, at *P* = 314 nm and *t*_s_ = 5 h, CPL was generated with varying *n* in a range of 1–2. It was found that at [CsBr] = 6–30 mmol L^-1^, |*g*_lum_| was generally maximized at *n* = 1.5, while small [CsBr] ( = 6 and 8 mmol L^-1^) led to high |*g*_lum_| (Fig. 4d and Supplementary Fig. 25).

Figure 4e summarizes |*g*_lum_| of LH-TiO_2_ NHs@CsPbBr_3_/Cs_4_PbBr_6_ fabricated at *α* = 85°–87°, *R*_d_ = 0.15–0.25 nm s^-1^, *P* = 100–650 nm, *n* = 1–8, [CsBr] = [PbBr_2_] = 4–30 mmol L^-1^, and *t*_s_ = 3–6 h. The core-to-shell chirality transfer makes |*g*_lum_| correlated with *g*_Ext, NHs_. However, correlation analysis revealed that |*g*_lum_| and *g*_Ext, NHs_ are positively associated but in a moderate manner (Supplementary Fig. 26), because the shell coating exhibits a non-negligible effect on |*g*_lum_| . The tangled influence of diverse core@shell formation parameters on *g*_lum_ causes the *g*_lum_-optimization tedious and time-consuming. |*g*_lum_| was optimized as 0.2 (Fig. 4e), outperforming most reports but possessing a significant gap with the maximum value of 2 (Fig. 1).

Although the manual optimization in Fig. 4 identifies several promising parameter regions, these plots represent low-dimensional projections of a substantially higher-dimensional design space. In the present core@shell system, *g*_lum_ is not governed by any single parameter independently, but by coupled effects among template geometry (*P*, *n*), GLAD conditions (*α*, *R*_d_, *β*), and shell-growth variables ([CsBr], [PbBr_2_], and *t*_s_). For example, the favorable precursor concentration ([CsBr], [PbBr_2_]) and *t*_s_ depend on the helix geometry, because *P* and *n* affect precursor diffusion, shell coverage, optical resonance, and chirality transfer. Therefore, simply combining the apparent maxima from one-factor scans does not necessarily yield the joint optimum. This coupling makes conventional trend-based optimization inefficient and motivates the use of deep-learning model that learns correlations across the full parameter vector rather than isolated one-dimensional trends.

### OptiCPL: multimodal deep-learning framework for *g*_lum_ amplification

**Fig. 5:**
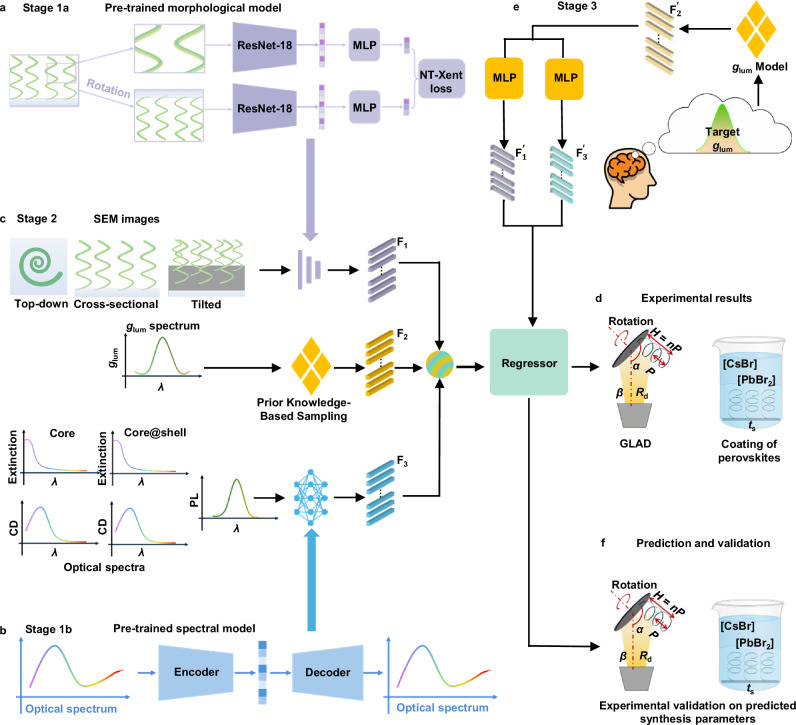
The workflow of OptiCPL. Stage 1 comprises two pre-trained modules: (**a** Stage 1a) morphological module, where a vision pre-trained model is fine-tuned on SEM images via self-supervised learning; (**b** Stage 1b) spectral module, where different types of spectra are encoded using an autoencoder-based framework. **c** Stage 2: embeddings extracted from the SEM images and diverse spectra are fused with *g*_lum_ spectra, and the combined representations are used to train a regressor toward (**d**) the experimental result. **e** Stage 3: a *g*_lum_-guided model is trained to capture correlations among *g*_lum_ spectrum, morphological features, and spectral features. Given a desired *g*_lum_ spectrum, the model reconstructs both morphological and spectral embeddings, which are then processed by the regressor to (**f**) predict the optimized set of fabrication parameters, and finally validated by the experiments.

To further enhance the *g*_lum_ values, we developed a few-shot deep-learning framework, OptiCPL, to guide and accelerate the *g*_lum_-enhancement process (Fig. 5). The OptiCPL framework consists of three major components: multimodal input pre-training (Stage 1a and 1b), multimodal model training (Stage 2), and inverse design (Stage 3).

#### Stage 1

Construction of the dataset and fine-tuning. The dataset of OptiCPL comprises 230 data points (128 independent fabrication and data augmentation; see Methods), each consisting of three major components: (1) fabrication parameters, including *α*, *R*_d_, *β* (the calibration factor of *R*_d_ at *α*), *P*, *n*, [CsBr], [PbBr_2_] and *t*_s_; (2) images of the chiral core@shells (three SEM images per sample, in top-down, cross-sectional and tilted views); and (3) optical spectra in the UV-visible region, including the extinction and CD spectra of the close-packed arrays of TiO_2_ NHs, as well as the extinction, CD, PL and *g*_lum_ spectra of the close-packed core@shell arrays. Despite this immense multimodal data, the dataset remains sparse relative to the vast design space. To overcome this data sparsity, a transfer learning strategy was applied to enhance the model’s capacity and robustness to generalize from limited samples and accurately infer structure–property relationships across diverse chiral composite systems. A pre-trained ResNet-18 model^71^, which was originally trained on a large-scale dataset comprising over 1.4 million natural images (ImageNet)^72^, was employed to encode SEM images after fine-tuning on our domain-specific dataset (Stage 1a, Fig. 5a). This pre-trained model prioritizes extracting high-level visual features and has been widely used in computer vision applications involving macroscopic objects, such as animals, vehicles, and scenes. However, a significant domain gap exists between natural images and the SEM images used in this study, which capture the fine-scale morphological details of material microstructures. Unlike natural images characterized by semantic textures and global object shapes, the SEM images exhibit dense, high-frequency patterns that are critical for understanding nanoscale architectures^73,74^. To address the domain gap between natural image features and microscopic textures present in SEM images, we employed a self-supervised contrastive learning strategy (SimCLR) to adapt a ResNet-18 encoder to our dataset^75^. By leveraging contrastive learning with random augmentations including rotation, cropping, and blurring on unlabeled SEM images, the SimCLR model learned transformation-invariant features that effectively capture the nano-morphological characteristics (Supplementary Figs. 27, 28). For the optical spectra, we implemented an autoencoder-based architecture, which was jointly pre-trained across multiple optical modalities to capture correlations among spectra and enable robust representation learning (Stage 1b, Fig. 5b). A spectral modality was constructed, comprising five types of one-dimensional spectra: extinction of chiral cores and core@shells, CD of chiral cores and core@shells, and PL of chiral core@shells. As these spectra collectively capture the CPL-relevant features, we designed an autoencoder model to jointly learn latent representations across all five spectral types. Each spectrum was passed through a dedicated encoder to project it into a shared latent space, followed by a fusion layer and individual decoders for reconstruction. Latent representations were learned through this pretraining phase, serving as input to the downstream multimodal model for the prediction of *g*_lum_.

#### Stage 2

Training a parameter prediction model using multimodal features. Both morphological and spectral embeddings (F_1_ and F_3_) were integrated and then fused with the corresponding prior-sampled *g*_lum_ spectra (F_2_) to form a comprehensive representation of each sample, which was then fed into a regression block (Stage 2, Fig. 5c). Several machine learning methods were applied, including Support Vector Regression (SVR)^76^, Random Forest (RF)^77^, Gradient Boosting^78^, AdaBoost^79^, K-Nearest Neighbors (KNN)^80^, Ridge^81^, ElasticNet^82^ and Multilayer Perceptron (MLP^83^), among which MLP achieved the best performance in terms of mean squared error (MSE) and the coefficient of determination *R*^2^ (Supplementary Fig. 29). The MLP model directly predicts an eight-dimensional vector corresponding to key fabrication parameters: *α*, *R*_d_, *β*, *P*, *n*, [CsBr], [PbBr_2_], and *t*_s_ (Fig. 5d). This generative modeling strategy enables an inverse mapping from target optical performance to candidate fabrication conditions, substantially improving the practicality and accessibility of OptiCPL for rational design of CPL materials. Through joint training on these modalities, OptiCPL captures deep physicochemical correlations and learns rich, semantically meaningful latent representations that reflect the underlying principles governing chiroptical behavior in complex material systems.

#### Stage 3

Inverse design enabled by *g*_lum_-guided reconstruction. For the design of CPL materials, researchers often desire a target *g*_lum_ spectrum but lack access to the corresponding morphology or spectral profiles, since morphological and spectral modalities are not directly observable prior to material fabrication. OptiCPL introduces an intermediate inverse reconstruction stage to bridge this gap. Specifically, two lightweight MLP regressors were trained to map targeted *g*_lum_ spectra (F_2_’) to the latent spaces of image and spectral features (F_1_’ and F_3_’, respectively, Stage 3, Fig. 5e). These reconstructed embeddings are then fed into the regressor (Stage 2, Fig. 5c) to predict optimal fabrication parameters for realizing the targeted CPL behavior (Fig. 5f). This strategy enables a highly accessible and user-friendly design pipeline: users are only required to input a sketch or approximation of the desired *g*_lum_ spectrum. The pretrained *g*_lum_-to-latent models then infer the corresponding morphological and spectral embeddings, which are subsequently decoded by OptiCPL into feasible fabrication conditions. A key advantage of this framework lies in its capacity to support property-driven inverse design, meaning that the user-desired optical targets can be seamlessly translated into actionable experimental parameters, where no prior knowledge of material structures or optical signatures are needed.

### Interpretability analysis

**Fig. 6:**
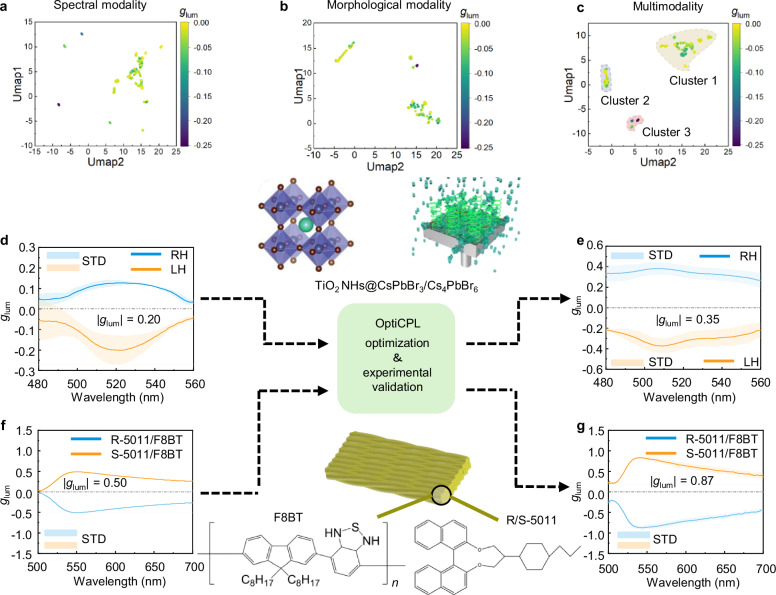
OptiCPL-guided optimization of *g*_lum_ of all-inorganic and organic chiral luminophores. **a** Spectral modality feature map after dimensionality reduction. **b** Morphological modality feature map after dimensionality reduction. **c** Multimodal feature map combining spectral and morphological features. **d, e** OptiCPL framework is applied to guide the optimization of |*g*_lum_| of the all-inorganic chiral nano-luminophores (TiO_2_ NHs@CsPbBr_3_/Cs_4_PbBr_6_) from (**d**) 0.20 (manually optimized) to (**e**) 0.35. **f, g** OptiCPL framework is applied to guide the optimization of |*g*_lum_| of organic chiral luminophores (*R*/*S*-5011/F8BT) from (**f**) 0.50 (manually optimized) to (**g**) 0.87. Solid lines: mean values; shaded areas: standard deviations (STD). Source data are provided as a Source Data file.

The incorporation of multimodal features in CPL-material design has been proven highly discriminative, as evidenced by the improved clustering observed in uniform manifold approximation and projection (UMAP) visualizations (Fig. 6a)^84^. UMAP is a nonlinear dimensionality reduction algorithm that preserves both local and global structure by optimizing a low-dimensional embedding based on the fuzzy topological relationships of high-dimensional data, which is more faithful than t-SNE^85^. Figure 6a, b present dimensionality-reduced feature maps derived from spectral and image modalities, respectively. In both cases, the features fail to form meaningful clusters correlated with high *g*_lum_ performance. Specifically, the spectral modality does not show distinct clustering in Fig. 6a. The morphological (image-based) modality yields three visually separable clusters in Fig. 6b, but the high-*g*_lum_ samples do not consistently aggregate in one single region. This limitation arises from the inherent nature of every single modality. The spectral modality primarily reflects the optical properties of the perovskite shells, which, on its own, lacks intrinsic chirality. Meanwhile, CPL generated from the chiral core@shell systems stems from the chirality transfer from the chiral cores to the achiral perovskite shells. Consequently, spectral data alone cannot fully capture the structural asymmetry or its underlying influence on emission dissymmetry. Similarly, the morphological features largely encode the chiral geometry but do not reflect the light-structure interactions, which is critical for the CPL generation. Thus, unimodal approaches fail to fully capture the underlying physical parameters governing CPL generation. In contrast, the multimodal feature embedding, which integrates both spectral and morphological information, successfully clusters high-*g*_lum_ samples into a distinct group (cluster 3, Fig. 6c). This result demonstrates that the fusion of multimodal data provides a more comprehensive representation of the optical behavior of the chiral nano-luminophores, enabling better identification of key factors influencing the CPL performance. The multimodal model captures both structural chirality and light–matter interactions, offering a more scientific basis for predictive modeling.

To gain deeper insight into how morphology modality works, post hoc attribution analyses were performed (Supplementary Fig. 30)^86^. The results show that the morphology modality of OptiCPL consistently highlights physically relevant structural regions, such as pitch textures and nanostructure-coverage areas, while assigning minimal attribution to background or irrelevant areas. This indicates that the model effectively captures the physically plausible structural cues. Moreover, additional tests show that these attribution patterns remain robust under extra Gaussian noises with 0.1–0.5 in normalized intensity, maintaining high correlation and significant spatial overlap of salient regions compared to the unperturbed baseline (Supplementary Fig. 31).

### Experimental validation

Based on the optimization using OptiCPL, the optimal fabrication conditions were identified: *α* = 87°, *β* = 0.33, *R*_d_ = 0.15 nm s^-1^, *P* = 370 nm, *n* = 1.07, [CsBr] = 5.6 mmol L^-1^, [PbBr_2_] = 6 mmol L^-1^, and *t*_s_ = 4.9 h. Under these OptiCPL-predicted conditions, the as-fabricated TiO_2_ NHs@CsPbBr_3_/Cs_4_PbBr_6_ exhibited *g*_lum_ = +0.33 and −0.35 for RH- and LH-core@shell nano-luminophores, respectively (Fig. 6e, Supplementary Figs. 32–34). Compared to the manually optimized |*g*_lum_| ( = 0.2) (Fig. 6d), the OptiCPL resulted in a 75% improvement of *g*_lum_ optimization. The OptiCPL-optimized |*g*_lum_| ( = 0.35) is the second-highest value of green CPL generated by all-inorganic chiral luminophores (Fig. 1). This success of OptiCPL comes from its treatment of each experiment as a multimodal data instance, rather than as a collection of independently varied parameters. The regressor was trained to simultaneously predict the multi-dimensional parameter vector, where the correlations and interactions among helical geometries, shell-growth conditions, and optical responses are implicitly encoded. In the inverse-design stage, a target *g*_lum_ spectrum is mapped back to morphology and spectral embeddings and then to a feasible fabrication parameter vector. Thus, OptiCPL performs joint optimization over the coupled experimental manifold, rather than selecting parameter values independently from one-factor trends. That makes the OptiCPL easily bypass the limit of manual optimization based on independent parameters in Fig. 4.

### Generalizability of OptiCPL

OptiCPL leverages complementary structural and physicochemical representations by jointly modeling both morphological and spectral modalities, enabling the model to capture the underlying determinants of CPL performance across diverse material systems. This multimodal integration not only enhances predictive accuracy but also promotes transferability and adaptability to other chiral luminophore systems, e.g., organic chiral luminophores composed of *R*/*S*-5011/F8BT. In the experiment, F8BT with a molecular weight of ≈ 36 kg mol^-1^ was utilized. As shown in Supplementary Fig. 35, OptiCPL was applied to a *R*/*S*-5011/F8BT dataset composed of three types of data: (1) fabrication parameters, (2) SEM images, and (3) UV-visible spectra of extinction, CD, PL and *g*_lum_ spectra. After data augmentation, the dataset contained 2,688 data points with the manually optimized |*g*_lum_| = 0.50 (Fig. 6f and Supplementary Fig. 36). The fabrication parameters obtained by training OptiCPL on this dataset to predict optimal *g*_lum_ values were experimentally verified, achieving |*g*_lum_| = 0.87 (Fig. 6g and Supplementary Fig. 37), which is the record-breaking value for F8BT-based chiral luminophores (Supplementary Fig. 38 and Supplementary Table 2). These results underscore the generalizability of OptiCPL and highlight its potential as a universal tool for AI-driven optimization of CPL materials.

#### Application scope of OptiCPL

OptiCPL is designed as a modular multimodal inverse-design workflow that jointly leverages the morphological information and optical observables (extinction, CD, PL, and *g*_lum_) of CPL materials. Here, the OptiCPL framework is validated on the chiral core@shell CPL system, and its generalizability is demonstrated by the successful transfer from all-inorganic to organic chiral luminophores (*R*/*S*-5011/F8BT). The applicability of OptiCPL to broader classes of CPL emitters, however, warrants further exploration. In particular, for intrinsically chiral molecular luminophores, the chiroptical performance is governed by intricate factors such as molecular configurations and local packing motifs^87^, which may not be fully captured by the current morphology-based modality in OptiCPL. Extending the framework to such systems would likely require integrating new data modalities, such as molecular graph representations^88^ or conformational fingerprints^89^, alongside the development of larger, domain-specialized datasets. While these frontiers remain to be fully explored, the modular architecture of OptiCPL provides a scalable foundation for evolving it into a universal platform for AI-driven CPL material discovery.

Beyond CPL optimization, the same modular formulation can be readily adapted for inverse design to optimize CD signals. By redefining the target property in the inverse-design stage from *g*_lum_ to a prescribed CD spectral profile, the framework called “OptiCD” can propose feasible fabrication parameter combinations for achieving desired CD features. As demonstrated in the proof-of-concept study in Supplementary Fig. 39, OptiCD successfully generated the fabrication conditions targeting a specified CD spectrum, and the experimental validation yielded a CD response in considerable agreement with the target. This extension illustrates that OptiCPL is not limited to CPL optimization but can be potentially generalized to other chiroptical design objectives.

#### Comparison with conventional data-driven methods

To benchmark OptiCPL against conventional data-driven workflows, six widely used machine learning baseline models were evaluated under the identical experimental constraints, including inverse models (RF/XGBoost/SVR) mapping a target *g*_lum_ spectrum to fabrication parameters, as well as forward models (RF/XGBoost^90^/SVR) predicting *g*_lum_ from the fabrication parameters followed by Bayesian optimization. Experimental validation showed that these conventional approaches either propose non-realizable conditions due to instrument constraints (like SVR) or yield substantially lower verified *g*_lum_ values (in a range of 0.08–0.14). In contrast, OptiCPL generates feasible parameter sets and achieves a markedly higher experimentally validated CPL performance, surpassing the best manually identified points in the original search space (Supplementary Fig. 40, and Supplementary Tables 3, 4).

In this work, we demonstrate a robust strategy for generating green CPL with high polarization purity by integrating GLAD-fabricated TiO_2_ NHs with all-inorganic perovskites (CsPbBr_3_/Cs_4_PbBr_6_). The TiO_2_ NHs serve as chiral templates effectively transferring their handedness to achiral perovskites conformally coated on the NHs, enabling the formation of chiral core@shell nano-luminophores. The chiroptical activity (characterized with CD) and CPL are tunable as a function of the helicity of chiral core and the shell-coating conditions, resulting in the manually experimental optimization of |*g*_lum_| = 0.20. To overcome the complexity and inefficiency of manual parameter tuning, we introduce OptiCPL, a few-shot multimodal deep-learning framework that integrates spectral and morphological features for inverse design. According to the prediction of OptiCPL followed by experimental validation, |*g*_lum_| is further increased by 75% (from 0.20 to 0.35) in the all-inorganic chiral core@shell nano-luminophores. In addition, OptiCPL is further applied to polymer-based chiral luminophores, leading to raising |*g*_lum_| of *R*/*S*-5011/F8BT from 0.50 (manually optimized) to 0.87. It underscores the robustness, generalizability, and transferability of this multimodal deep-learning framework. Collectively, these findings establish a versatile platform for designing CPL-active materials, highlight the critical role of deep learning in accelerating optimization of chiroptical materials, and open new opportunities for advancing optoelectronic applications in information storage, secure communication, three-dimensional displays, and bioimaging.

## Methods

### Materials

Chemicals, including CsBr (99.999%, Yingkou Shangneng Photoelectricmaterial Co., Ltd.), PbBr_2_ (99.999%, Yingkou Shangneng Photoelectricmaterial Co., Ltd.), oleylamine (OAm, 80 − 90%, Aldrich), poly[(9,9-di-n-octylfluorenyl-2,7-diyl)-alt-(benzo[2,1,3]thiadiazol-4,8-diyl)] (F8BT, Luminescence Technology Corp. Batch LT-S957-250317001), 35,902 g mol^-1^, Đ = 3.24), and S-(13bR)-5,6-dihydro-5-(trans-4-propylcyclohexyl)-4H-dinaphtho[2,1-f:1’,2’-h][1,5]dioxonin (S-5011, 99%, Macklin, batch: Lot#C15307502, 450.62 g mol^-1^), R-(13bR)-5,6-dihydro-5-(trans-4-propylcyclohexyl)-4H-dinaphtho[2,1-f:1’,2’-h][1,5]dioxonin (R-5011, 99%, Bidepharma, batch: BD01413661, 450.61 g mol^-1^), toluene (Aldrich) and *N*, *N*-dimethylformamide (DMF, Aldrich) were used as received. TiO_2_ pellets (99.9%) were purchased from Kurt J Lesker Company.

### GLAD-based fabrication of TiO_2_ NHs

Close-packed arrays of TiO_2_ NHs were deposited on a supporting substrate (sapphires (MTL, Hong Kong) and silicon wafers (Semiconductor Wafer, Inc.)) using GLAD. Then the as-deposited samples were sequentially cleaned with deionized water, acetone, and ethanol, followed by being dried with N_2_. In a custom-built physical vapor deposition system (JunSun Tech Co. Ltd., Taiwan), GLAD was performed at *α* in a range of 85°–89° and in a high vacuum of 10^−7^–10^−6^ torr. TiO_2_ pellets were evaporated via electron-beam evaporation to condense on supporting substrates in an area of 1.5$\times$1.5 cm^2^. *R*_d_ of TiO_2_ was monitored by a quartz crystal microbalance as 1.5 Å s⁻¹ (corresponding to *α* = 0°), at an electron-beam accelerating voltage of 8 kV and power of 17%–24%. At a given *α*, the deposition rate on the surface of the supporting substrate (*R*_d_) was calibrated by

3 $${R}_{{{\rm{d}}}}=\beta {R}_{{{\rm{d}}}}$$

For example, *β* = 1/3 at *α* of 87°, and thus *R*_d_ = 0.5 Å s⁻¹. During GLAD, unidirectional rotation of substrates in clockwise and counterclockwise enabled the sculpture of TiO_2_ NHs in RH and LH, respectively^91^. *P* (in units of nm per revolution) was engineered by,

4 $$P=360{R}_{{{\rm{d}}}}/{R}_{{{\rm{r}}}}$$

where *R*_r_ is the substrate rotation rate (in ° s⁻¹). *P* was adjusted in a range of 100–650 nm, and experimentally measured by

5 $$P=H/n$$

where *H* is the helical height measured via SEM, and *n*, equal to the number of circles in which supporting substrates were rotated during GLAD, was tuned in a range of 1–8 (Fig. 2b).

### Conformal coating of TiO_2_ NHs with CsPbBr_3_/Cs_4_PbBr_6_

Under ambient conditions, ligand-assisted co-precipitation was performed for conformally coating TiO_2_ NHs with cesium lead bromides. The perovskite precursor solution was prepared by dissolving CsBr and PbBr_2_ in 10 mL of DMF, followed by stirring for 3 h at room temperature. TiO_2_ NHs were immersed in the perovskite precursor solution mixed with 1 mL of OAm for a period of *t*_s_. Then, spin coating of the modified TiO_2_ NHs with 0.5 mL toluene (acting as an antisolvent) was performed at 3000 rpm for 30 s, resulting in the formation of TiO_2_ NHs@CsPbBr_3_/Cs_4_PbBr_6_. [CsBr], [PbBr_2_] and *t*_s_ were tuned in a range of 4–30 mmol L^-1^ and 3–6 h, respectively, to engineer and optimize the shell coating.

### Fabrication of CsPbBr_3_/Cs_4_PbBr_6_ nanocrystals

The perovskite precursor solution (with [CsBr] = [PbBr_2_] = 4 mmol L^-1^) was thoroughly mixed with 1 mL of OAm. 1 mL of the OAm-mixed perovskite precursor solution was rapidly injected into 10 mL of toluene under vigorous stirring, leading to the formation of CsPbBr_3_/Cs_4_PbBr_6_ nanocrystals.

### Generation of chiral organic luminophores composed of *R*/*S*-5011/F8BT

F8BT and *R*/*S*-5011 (10–40 mg) were dissolved in 1 mL of toluene and stirred for 3 h. Then 30 µL of the mixture solution was drop-cast on silicon wafers or sapphires, followed by spin-coating at 1000–4000 rpm for 20–90 s. The as-formed 5011/F8BT thin films were annealed on a hot stage at various temperatures (140–250 ℃) and durations (5–30 min).

### Material characterizations

The samples were mechanically split, leaving the freshly exposed surfaces for the characterization with SEM (Thermo Scientific Scios 2 DualBeam). Nanomaterials were scratched off the supporting substrates and well dispersed in ethanol via ultrasonication for 15 min. Several drops of the mixture were applied to a lacey carbon film on a grid structure (Electron Microscopy Sciences). The grid was dried under ambient conditions and inspected by TEM (FEI Tecnai F20 and TS12). The nanostructures were characterized with XPS (Thermo Scientific K-Alpha using Al as the excitation source) and XRD (X’Pert PRO instrument (PANalytcal, Netherlands) with a scan range from 5° to 90° at 3° min^−1^; Cu Kα radiation, 40 kV, 40 mA, wavelength of 1.5418 Å).

### Optical characterizations

ChirascanV100 (AppliedPhotophysics) was used to monitor UV-visible extinction, CD, PL and CPL spectra of chiral samples deposited on sapphires, exposed to linearly polarized incident at a wavelength of 370 nm and 330 nm for TiO_2_ NHs@CsPbBr_3_/Cs_4_PbBr_6_ and 5011/F8BT, respectively. To measure extinction and CD spectra, linearly polarized light was incident along the normal direction of sapphires, i.e., $\gamma=\,$0° or 180° (Supplementary Fig. 4). Eight CD spectra were measured at ($\varphi,\gamma$) of (0°, 0°), (45°, 0°), (90°, 0°), (135°, 0°), (0°, 180°), (45°, 180°), (90°, 180°), and (135°, 180°), and then algebraically averaged to obtain an average CD spectrum, to eliminate linear birefringence and linear dichroism^92^. For measuring CPL of chiral luminophores, four CPL spectra were recorded at (0°, 30°), (45°, 30°), (90°, 30°), (135°, 30°) (whereby $\gamma$ was adjusted in a range of 25°–43° and set as 30° with the maximization of *g*_lum_) and then algebraically averaged to obtain a mean CPL spectrum (Supplementary Fig. 7).

### OptiCPL model construction and inverse design

The OptiCPL dataset for TiO_2_ NHs@CsPbBr_3_/Cs_4_PbBr_6_ comprised 230 data instances derived from 128 independent syntheses. Each independent sample contains three types of information: (i) Fabrication parameters, including *α*, *R*_*d*_, *β*, *P*, *n*, [CsBr], [PbBr_2_], and *t*_*s*_; (ii) SEM images of the chiral core@shell nano-luminophores; and (iii) optical spectra, consisting extinction and CD spectra of both TiO_2_ NH templates and core@shell arrays, as well as PL and *g*_lum_ spectra of the core@shell arrays. For *R*/*S*-5011/F8BT, the OptiCPL dataset contained the fabrication parameters ([F8BT], [5011], spin-coating speed and duration, annealing temperature, and annealing duration), SEM images of polymer films, and optical spectra of extinction, CD, PL, and glum of polymer films. Data augmentation and normalization scaling were applied.

The morphology modality uses an ImageNet-pretrained ResNet-18^71^ as the SEM encoder, followed by SimCLR-based contrastive learning strategy to bridge the domain gap between natural images and SEM images^75^. Positive image pairs were generated using random resized cropping to 224 × 224 pixels, grayscale conversion, and Gaussian blurring, enabling the encoder to learn transformation-invariant morphology representations (Supplementary Fig. 23). The classification head was replaced by a projection head to generate normalized 128-dimensional embeddings.

The spectral modality uses a multi-branch autoencoder to encode five optical spectra: PL, extinction of the core@shell sample, CD of the core@shell sample, CD of the TiO_2_ template, and extinction of the TiO_2_ template. Each spectrum was encoded by an independent multilayer perceptron, and the latent vectors were concatenated and fused into a shared spectral representation. The decoder reconstructs each spectral modality from the shared latent representation, encouraging the model to capture correlations among optical responses relevant to CPL generation. For the *R*/*S*-5011/F8BT polymers, the same multimodal regression and inverse-design workflow was applied via replacing the output vector with the corresponding polymer-processing parameters, including [F8BT], [5011], spin-coating speed and duration, annealing temperature, and annealing duration.

In the multimodal regression stage, morphology embeddings, spectral embeddings, *g*_lum_ spectra, and optical descriptors were concatenated and passed into a fully connected neural network to predict the eight fabrication parameters. The inverse-design module then uses a target *g*_lum_ spectrum to reconstruct the corresponding morphology and spectral latent representations, which are subsequently processed by the trained regressor to output an optimized set of experimentally feasible fabrication parameters. Model performance was evaluated on held-out test samples after inverse transformation into the original parameter scales. For the attempt on CD-targeted design, the same inverse-design architecture was re-parameterized by replacing the target *g*_lum_ with a prescribed CD response, while keeping the multimodal encoders and feasibility-aware regression pipeline unchanged. The model architecture, hyperparameters, and training details are provided in the released code.

### Reporting summary

Further information on research design is available in the Nature Portfolio Reporting Summary linked to this article.

## Supplementary information

Supplementary Information

Reporting Summary

Transparent Peer Review file

## Source data

Source Data

## Data Availability

The data that support the findings of this study, including experimental data for model training, are available from Zenodo^93^ and from the corresponding authors upon request. Source data are provided with this paper.

## Code availability

The code and models of OptiCPL are available from Zenodo^93^ and from the corresponding authors upon request.

## References

[1] Yang, L. et al. Chiral ligand-free, optically active nanoparticles inherently composed of chiral lattices at the atomic scale. *Small* **16**, 2001473 (2020). 10.1002/smll.202001473 32419372

[2] Li, G., Wang, Y., Lu, H. & Huang, Z. Amplification of chiral Raman scattering: A review of resonance Raman optical activity and surface enhanced Raman optical activity. *Adv. Mater. Interfaces* **12**, 2400930 (2025).

[3] Zhan, X. et al. 3d laser displays based on circularly polarized lasing from cholesteric liquid crystal arrays. *Adv. Mater.* **33**, e2104418 (2021). 34337797 10.1002/adma.202104418

[4] Lin, S. et al. Photo-triggered full-color circularly polarized luminescence based on photonic capsules for multilevel information encryption. *Nat. Commun.* **14**, 3005 (2023). 37231049 10.1038/s41467-023-38801-1 PMC10212932

[5] Guo, Q. et al. Multimodal-responsive circularly polarized luminescence security materials. *J. Am. Chem. Soc.* **145**, 4246–4253 (2023). 10.1021/jacs.2c13108 36724236

[6] Stachelek, P., Mackenzie, L., Parker, D. & Pal, R. Circularly polarised luminescence laser scanning confocal microscopy to study live cell chiral molecular interactions. *Nat. Commun.* **13**, 553 (2022). 35087047 10.1038/s41467-022-28220-z PMC8795401

[7] Shang, X. et al. Supramolecular nanostructures of chiral perylene diimides with amplified chirality for high-performance chiroptical sensing. *Adv. Mater.* **29**, 1605828 (2017). 10.1002/adma.201605828 28370408

[8] Wei, X. Q. et al. Enantioselective photoinduced cyclodimerization of a prochiral anthracene derivative adsorbed on helical metal nanostructures. *Nat. Chem.* **12**, 551–559 (2020). 32313237 10.1038/s41557-020-0453-0

[9] Sang, Y., Han, J., Zhao, T., Duan, P. & Liu, M. Circularly polarized luminescence in nanoassemblies: Generation, amplification, and application. *Adv. Mater.* **32**, e1900110 (2020). 31394014 10.1002/adma.201900110

[10] Wang, X., Ma, S., Zhao, B. & Deng, J. Frontiers in circularly polarized phosphorescent materials. *Adv. Funct. Mater.* **33**, 2214364 (2023).

[11] Jiang, S. & Kotov, N. A. Circular polarized light emission in chiral inorganic nanomaterials. *Adv. Mater.* **35**, 2108431 (2023). 10.1002/adma.202108431 35023219

[12] Yang, X. et al. Recent progress of circularly polarized luminescence materials from chinese perspectives. *CCS Chem.* **5**, 2760–2789 (2023).

[13] Wang, J. et al. Chiral phosphine-copper iodide hybrid cluster assemblies for circularly polarized luminescence. *J. Am. Chem. Soc.* **143**, 10860–10864 (2021). 34279083 10.1021/jacs.1c05476

[14] Sen, S., Mukhopadhyay, R., Choi, S., Hwang, I. & Kim, K. Spatiotemporal segregation of chiral supramolecular polymers. *Chem* **9**, 624–636 (2023).

[15] Wang, Z., Liu, S., Quan, Y. & Cheng, Y. Tunable aicpl of (s)-binaphthyl-based three-component polymers via fret mechanism. *Macromol. Rapid Commun.* **38**, 1700150 (2017). 10.1002/marc.201700150 28488396

[16] Jia, T. et al. Enantiomeric alkynyl-protected Au10 clusters with chirality-dependent radiotherapy enhancing effects. *Nano Today* **39**, 101222 (2021).

[17] Liu, S. et al. Circularly polarized perovskite luminescence with dissymmetry factor up to 1.9 by soft helix bilayer device. *Matter* **5**, 2319–2333 (2022).

[18] Zhang, Y. et al. Circularly polarized luminescence in chiral materials. *Matter* **5**, 837–875 (2022).

[19] Yao, J. et al. Efficient spin-light-emitting diodes with tunable red to near-infrared emission at room temperature. *Adv. Mater.* **37**, e2413669 (2025). 39887568 10.1002/adma.202413669 PMC11899487

[20] Zhang, R. et al. Energy transfer for constructing circularly polarized luminescence materials: Recent progress and future prospects. *Adv. Funct. Mater.* **35**, 2417308 (2024).

[21] Zhang, C., Li, Z., Dong, X., Niu, Y. & Zang, S. Multiple responsive CPL switches in an enantiomeric pair of perovskite confined in lanthanide MOFs. *Adv. Mater.* **34**, e2109496 (2022). 35020258 10.1002/adma.202109496

[22] Zhao, B., Yu, H., Pan, K., Tan, Z. & Deng, J. Multifarious chiral nanoarchitectures serving as handed-selective fluorescence filters for generating full-color circularly polarized luminescence. *ACS Nano* **14**, 3208–3218 (2020). 32022541 10.1021/acsnano.9b08618

[23] Jia, S. et al. Dual-direction circularly polarized luminescence materials with on-demand handedness and superior flexibility. *Adv. Funct. Mater.* **34**, 2410206 (2024).

[24] Ru, Y. et al. Full-color circularly polarized luminescence of cspbx(3) nanocrystals triggered by chiral carbon dots. *Adv. Mater.* **35**, e2207265 (2023). 36408928 10.1002/adma.202207265

[25] Liu, P. et al. Optically active perovskite cspbbr_3_ nanocrystals helically arranged on inorganic silica nanohelices. *Nano Lett.* **20**, 8453–8460 (2020). 32880460 10.1021/acs.nanolett.0c02013

[26] Veksler, M. et al. Circularly polarized light emission from single chiral hedgehog particles coated with nanofilms of achiral perovskites. *Adv. Mater.* **37**, e18765 (2025). 40905504 10.1002/adma.202418765 PMC12617027

[27] Zheng, H. Z. et al. Circularly polarized luminescent carbon dot nanomaterials of helical superstructures for circularly polarized light detection. *Adv. Opt. Mater.* **6**, 1801246 (2018).

[28] Zhou, Y. et al. Helical-caging enables single-emitted large asymmetric full-color circularly polarized luminescence. *Nat. Commun.* **15**, 251 (2024). 38177173 10.1038/s41467-023-44643-8 PMC10767107

[29] Ji, M., Zhao, W., Li, M. & Chen, C. Circularly polarized luminescence with high dissymmetry factors for achiral organic molecules in solutions. *Nat. Commun.* **16**, 2940 (2025). 40133332 10.1038/s41467-025-58355-8 PMC11937319

[30] Tschierske, C. Mirror symmetry breaking in liquids and liquid crystals. *Liq. Cryst.* **45**, 2221–2252 (2018).

[31] Huang, S. et al. Synthesis and properties of chiral liquid crystal copolymers with tunable circularly polarized luminescence. *Eur. Polym. J.* **219**, 113398 (2024).

[32] Foster, E., Jones, R., Lavigueur, C. & Williams, V. Structural factors controlling the self-assembly of columnar liquid crystals. *J. Am. Chem. Soc.* **128**, 8569–8574 (2006). 16802823 10.1021/ja0613198

[33] Yang, X., Zhou, M., Wang, Y. & Duan, P. Electric-field-regulated energy transfer in chiral liquid crystals for enhancing upconverted circularly polarized luminescence through steering the photonic bandgap. *Adv. Mater.* **32**, 2000820 (2020). 10.1002/adma.202000820 32378267

[34] Dai, Y. et al. Machine-learning-driven g-quartet-based circularly polarized luminescence materials. *Adv. Mater.* **36**, e2310455 (2024). 37983564 10.1002/adma.202310455

[35] Li, B. et al. Accelerating ionizable lipid discovery for mrna delivery using machine learning and combinatorial chemistry. *Nat. Mater.* **23**, 1002–1008 (2024). 38740955 10.1038/s41563-024-01867-3

[36] Zhang, S. et al. Deep learning-assisted design of novel donor–acceptor combinations for organic photovoltaic materials with enhanced efficiency. *Adv. Mater.* **37**, 2407613 (2025). 10.1002/adma.202407613 39648547

[37] Szymanski, N. et al. An autonomous laboratory for the accelerated synthesis of novel materials. *Nature* **624**, 86–91 (2023). 38030721 10.1038/s41586-023-06734-w PMC10700133

[38] Fan, Q. et al. Entropy in catalyst dynamics under confinement. *Chem. Sci.* **15**, 18303–18309 (2024). 39464620 10.1039/d4sc05399k PMC11500834

[39] Zhu, J. & Cheng, J. Machine learning potential for electrochemical interfaces with hybrid representation of dielectric response. *Phys. Rev. Lett.* **135**, 018003 (2025). 40743077 10.1103/48ct-3jxm

[40] Im, S. et al. Investigating chiral morphogenesis of gold using generative cellular automata. *Nat. Mater.* **23**, 977–983 (2024). 38693448 10.1038/s41563-024-01889-x

[41] Zhang, M. et al. Revealing transition state stabilization in organocatalytic ring-opening polymerization using data science. *Angew. Chem. Int. Ed.* **64**, e202502090 (2025). 10.1002/anie.202502090 40146080

[42] Zeni, C. et al. A generative model for inorganic materials design. *Nature* **639**, 624–632 (2025). 39821164 10.1038/s41586-025-08628-5 PMC11922738

[43] Liu, X. et al. Design of circularly polarized phosphorescence materials guided by transfer learning. *Nat. Commun.* **16**, 4970 (2025). 40436886 10.1038/s41467-025-60310-6 PMC12119802

[44] Huang, Z. & Liu, J. Chiroptically active metallic nanohelices with helical anisotropy. *Small* **13**, 1701883 (2017). 10.1002/smll.201701883 28960853

[45] Wu, Z. et al. Low-temperature-deposited TiO_2_ nanopillars for efficient and flexible perovskite solar cells. *Adv. Mater. Interfaces* **8**, 2001512 (2021).

[46] Xiao, T. et al. Sensitive, high-speed, and broadband perovskite photodetectors with built-in tio(2) metalenses. *Small* **17**, e2102694 (2021). 34510709 10.1002/smll.202102694

[47] Fang, Y., Yang, L. & Huang, Z. Advanced optoelectronic applications of nanopillar arrays fabricated by glancing angle deposition. *Nanomaterials* **15**, 1555 (2025). 41149524 10.3390/nano15201555 PMC12566347

[48] Zhang, S. et al. Extracellular nanomatrix-induced self-organization of neural stem cells into miniature substantia nigra-like structures with therapeutic effects on parkinsonian rats. *Adv. Sci.* **6**, 1901822 (2019). 10.1002/advs.201901822 PMC6918115 31871862

[49] Peng, K., Yu, L. & Zhang, M. Synthesis of cspbx3 lead halide perovskite nanocrystals in flower-like tio2 matrices with enhanced stability. *J. Lumin.* **253**, 119428 (2023).

[50] Huang, Z., Hawkeye, M. & Brett, M. Enhancement in broadband and quasi-omnidirectional antireflection of nanopillar arrays by ion milling. *Nanotechnology* **23**, 275703 (2012). 22705498 10.1088/0957-4484/23/27/275703

[51] Sun, P. et al. Highly efficient large-area flexible perovskite solar cells containing tin oxide vertical nanopillars without oxygen vacancies. *ACS Appl. Energy Mater.* **5**, 3568–3577 (2022).

[52] Deng, J. & Huang, Z. Radiative loss-determined circular dichroism of plasmonic nanospirals with bendable stability of chiroptical activity. *RSC Adv.* **6**, 84348–84353 (2016).

[53] Huang, Z. & Bai, F. Wafer-scale, three-dimensional helical porous thin films deposited at a glancing angle. *Nanoscale* **6**, 9401–9409 (2014). 24838479 10.1039/c4nr00249k

[54] Deng, J., Fu, J., Ng, J. & Huang, Z. Tailorable chiroptical activity of metallic nanospiral arrays. *Nanoscale* **8**, 4504–4510 (2016). 26530309 10.1039/c5nr06291h

[55] Yang, L. et al. Nanohelix-induced optical activity of liquid metal nanoparticles. *Small* **18**, 2200620 (2022). 10.1002/smll.202200620 35319827

[56] Ni, Z. Y. et al. Significant enhancement of circular polarization in light emission through controlling helical pitches of semiconductor nanohelices. *ACS Nano* **17**, 20611–20620 (2023). 37796740 10.1021/acsnano.3c07663 PMC10604094

[57] Han, T. et al. A roadmap for the commercialization of perovskite light emitters. *Nat. Rev. Mater.* **7**, 757–777 (2022).

[58] De Bastiani, M. et al. Inside perovskites: Quantum luminescence from bulk Cs_4_PbBr_6_ single crystals. *Chem. Mater.* **29**, 7108–7113 (2017).

[59] Zhao, J. et al. Titanium nanopillar arrays functioning as electron transporting layers for efficient, anti-aging perovskite solar cells. *Small* **17**, 2004778 (2021). 10.1002/smll.202004778 33325649

[60] Ma, Y. et al. Chiral nanoparticles with enhanced thermal stability of chiral structures through alloying. *Small* **18**, 2107657 (2022). 10.1002/smll.202107657 35174949

[61] Yang, L. et al. Binary chiral nanoparticles exhibit amplified optical activity and enhanced refractive index sensitivity. *Small* **16**, 1906048 (2020). 10.1002/smll.201906048 31961482

[62] To, W., Fu, J., Yang, X., Roy, V. & Huang, Z. Porosification-reduced optical trapping of silicon nanostructures. *Nanoscale* **4**, 5835–5839 (2012). 22899347 10.1039/c2nr31680c

[63] Sun, P., Liu, J., Yan, M. & Huang, Z. Helical nanoparticle-induced enantiospecific adsorption of N3 dyes. *Chem. Commun.* **54**, 4270–4273 (2018). 10.1039/c8cc01836g 29629717

[64] Ni, Z. et al. Extension of compositional space to the ternary in alloy chiral nanoparticles through galvanic replacement reactions. *Adv. Sci.* **7**, 2001321 (2020). 10.1002/advs.202001321 PMC7710001 33304745

[65] Lau, W., Yang, L., Bai, F. & Huang, Z. Weakening circular dichroism of plasmonic nanospirals induced by surface grafting with alkyl ligands. *Small* **12**, 6698–6702 (2016). 27805771 10.1002/smll.201602236

[66] Schäferling, M., Dregely, D., Hentschel, M. & Giessen, H. Tailoring enhanced optical chirality: Design principles for chiral plasmonic nanostructures. *Phys. Rev. X* **2**, 031010 (2012).

[67] Tang, Y. & Cohen, A. Optical chirality and its interaction with matter. *Phys. Rev. Lett.* **104**, 163901 (2010). 20482049 10.1103/PhysRevLett.104.163901

[68] Lau, W., Bai, F. & Huang, Z. Ballistic glancing angle deposition of inclined ag nanorods limited by adatom diffusion. *Nanotechnology* **24**, 465707 (2013). 24164870 10.1088/0957-4484/24/46/465707

[69] Yang, L. et al. Chiral nanoparticle-induced enantioselective amplification of molecular optical activity. *Adv. Funct. Mater.* **29**, 1807307 (2019).

[70] Xiao, C. et al. Microfluidic-based metal enhanced fluorescence for capillary electrophoresis by Ag nanorod arrays. *Nanotechnology* **25**, 225502 (2014). 24833562 10.1088/0957-4484/25/22/225502

[71] He, K., Zhang, X., Ren, S. & Sun, J. Deep residual learning for image recognition. *IEEE Conference on Computer Vision and Pattern Recognition* 770–778 (2016).

[72] Deng, J. et al. Imagenet: A large-scale hierarchical image database. *IEEE Conference on Computer Vision and Pattern Recognition* 248–255 (2009).

[73] Kim, J. et al. Self-supervised machine learning framework for high-throughput electron microscopy. *Sci. Adv.* **11**, eads5552 (2025). 40173219 10.1126/sciadv.ads5552 PMC11963987

[74] Bendidi, I. et al. Exploring self-supervised learning biases for microscopy image representation. *Biol. Imaging* **4**, e12 (2024). 39776611 10.1017/S2633903X2400014X PMC11704125

[75] Chen, T., Kornblith, S., Norouzi, M. & Hinton, G. A simple framework for contrastive learning of visual representations. International Conference on Machine Learning, 1597–1607 (PMLR, 2020).

[76] Drucker, H., Burges, C., Kaufman, L., Smola, A. & Vapnik, V. Support vector regression machines. *Adv. Neural Inf. Process. Syst.* **9**, 155–161 (1996).

[77] Breiman, L. Random forests. *Mach. Learn.* **45**, 5–32 (2001).

[78] Friedman, J. Greedy function approximation: A gradient boosting machine. *Ann. Stat.* **29**, 1189–1232 (2001).

[79] Freund, Y. & Schapire, R. A decision-theoretic generalization of on-line learning and an application to boosting. *J. Comput. Syst. Sci.* **55**, 119–139 (1997).

[80] Cover, T. & Hart, P. Nearest neighbor pattern classification. *IEEE Trans. Inf. Theory* **13**, 21–27 (1967).

[81] Hoerl, A. & Kennard, R. Ridge regression: Biased estimation for nonorthogonal problems. *Technometrics* **12**, 55–67 (1970).

[82] Zou, H. & Hastie, T. Regularization and variable selection via the elastic net. *J. R. Stat. Soc. Ser. B. Stat. Methodol.* **67**, 301–320 (2005).

[83] Rumelhart, D., Hinton, G. & Williams, R. Learning representations by back-propagating errors. *Nature* **323**, 533–536 (1986).

[84] McInnes, L. & Mclnnes, L. Uniform manifold approximation and projection. *Nat. Rev. Methods Prim.* **4**, 82 (2024).

[85] Maaten, L. & Hintin, G. Visualizing data using t-SNE. *J. Mach. Learn. Res.* **9**, 2579–2605 (2008).

[86] Selvaraju, R. et al. Grad-CAM: Visual Explanations from Deep Networks via Gradient-Based Localization. *2017 IEEE International Conference on Computer Vision (ICCV)*, 618-626, (2017).

[87] Nitti, A. & Pasini, D. Aggregation-Induced Circularly Polarized Luminescence: Chiral Organic Materials for Emerging Optical Technologies. *Adv. Mater.* **32**, 1908021 (2020). 10.1002/adma.201908021 32173906

[88] Gilmer, J., Schoenholz, S., Riley, P., Vinyals, O. & Dahl, G. Neural message passing for quantum chemistry. ICML, 1263–1272, 10.48550/arXiv.1704.01212 (2017).

[89] Axen, S. et al. A Simple Representation of Three-Dimensional Molecular Structure. *J. Med. Chem.* **60**, 7393–7409 (2017). 28731335 10.1021/acs.jmedchem.7b00696 PMC6075869

[90] Chen T. & Guestrin C. Xgboost: A scalable tree boosting system. *Proceedings of the 22nd acm sigkdd international conference on knowledge discovery and data mining*, 785–794 (2016).

[91] Qin, P. et al. Dynamic kinetic resolution of helical polycyclic arenes directed at inorganic chiral surfaces deposited via substrate rotation. *Chem* **12**, 102720 (2026).

[92] Yao, Y. et al. Extracting pure circular dichroism from hierarchically structured CdS magic cluster films. *ACS Nano* **16**, 20457–20469 (2022). 36395373 10.1021/acsnano.2c06730

[93] Sun, H. et al. Multimodal deep-learning optimization of chiroptical properties in all-inorganic perovskite-coated TiO_2_ nanohelices and inverse-design transfer to organic chiral luminophores. *Zenodo*, 10.5281/zenodo.20061189 (2026). 10.1038/s41467-026-74010-2 PMC13396460 42243137

