# Supplementary material for: Multimodal deep-learning optimization of chiroptical properties in all-inorganic perovskite-coated TiO2 nanohelices and inverse-design transfer to organic chiral luminophores
Source: Nat Commun. 2026 Jun 4;17:7180. doi: 10.1038/s41467-026-74010-2 (PMC13396460; doi:10.1038/s41467-026-74010-2)
Supplement: Supplementary file 1 — Supplementary Information [file 41467_2026_74010_MOESM1_ESM.pdf]

Supplementary Information

Multimodal deep-learning optimization of chiroptical properties in all-inorganic perovskite-coated TiO_2 nanohelices and inverse-design transfer to organic chiral luminophores

Haifeng Sun,[1,2#] Yilun Zhang,[1,2,3#] Xiao Chen,[2] Wentao Wang,[4] Guang-Jie Xia,[3,5*] and Zhifeng Huang[1,2,6*]

1. Joint Institute of Advanced Materials and Green Energy Research, Great Bay University, Dongguan 523000, Guangdong Province, P. R. China
2. Department of Chemistry, The Chinese University of Hong Kong, Shatin, N. T., Hong Kong SAR 999077, P. R. China
3. Centre for Intelligent Computing, Great Bay Institute for Advanced Study, Dongguan 523000, Guangdong Province, P. R. China
4. School of Automation, Chongqing University of Posts and Telecommunications, Chongqing 400065, Chongqing, P. R. China
5. School of Physical Sciences, Great Bay University, Dongguan 523000, Guangdong Province, P. R. China
6. Shenzhen Research Institute, The Chinese University of Hong Kong, No. 10, 2nd Yuexing Road, Nanshan, Shenzhen, Guangdong Province, 518057, P. R. China

Correspondence: xiagj@gbu.edu.cn; zfhuang@cuhk.edu.hk

#These authors contributed equally: Haifeng Sun and Yilun Zhang.

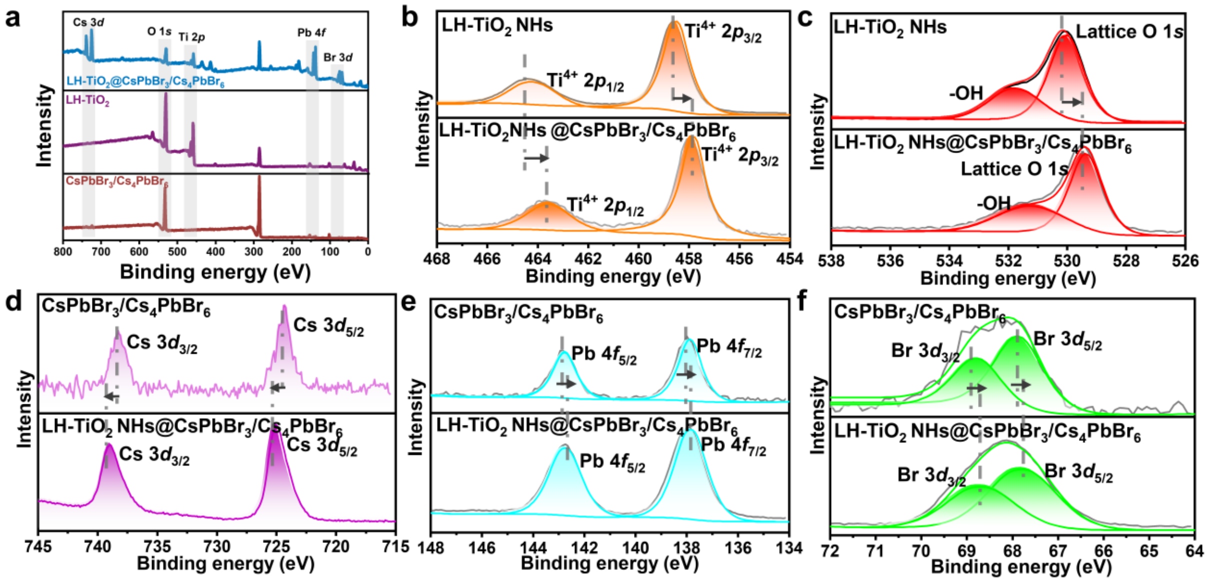

Supplementary Figure 1| Characterization of TiO_2 NHs, $CsPbBr_3/Cs_4PbBr_6$ and TiO_2 NHs@$CsPbBr_3/Cs_4PbBr_6$ by X-ray photoelectron spectroscopy (XPS): a XPS full survey scan; high-resolution XPS spectra of **b** Ti 2*p*, **c** O 1*s*, **d** Cs 3*d*, **e** Pb 4*f*, and **f** Br 3*d*.

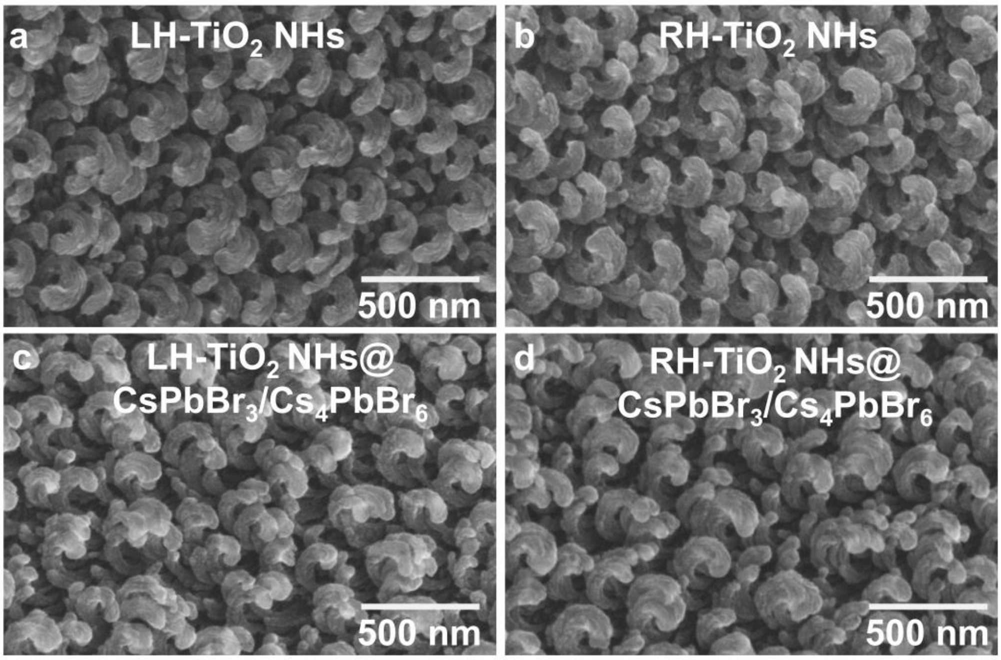

Supplementary Figure 2| Nanostructural characterization of TiO_2 NHs and TiO_2 NHs@$CsPbBr_3/Cs_4PbBr_6$ by SEM. SEM top-down images: **a** LH-TiO_2 NHs (P = 310 nm, n = 1); **b** RH-TiO_2 NHs (P = 300 nm, n = 1); **c** LH-core@shells (P = 315 nm, n = 1); **d** RH-core@shells (P = 310 nm, n = 1). Shell-coating condutions: [CsBr] = [$PbBr_2$] = 4 mmol L^{-1}, and soaking period (t_s) of 5 h.

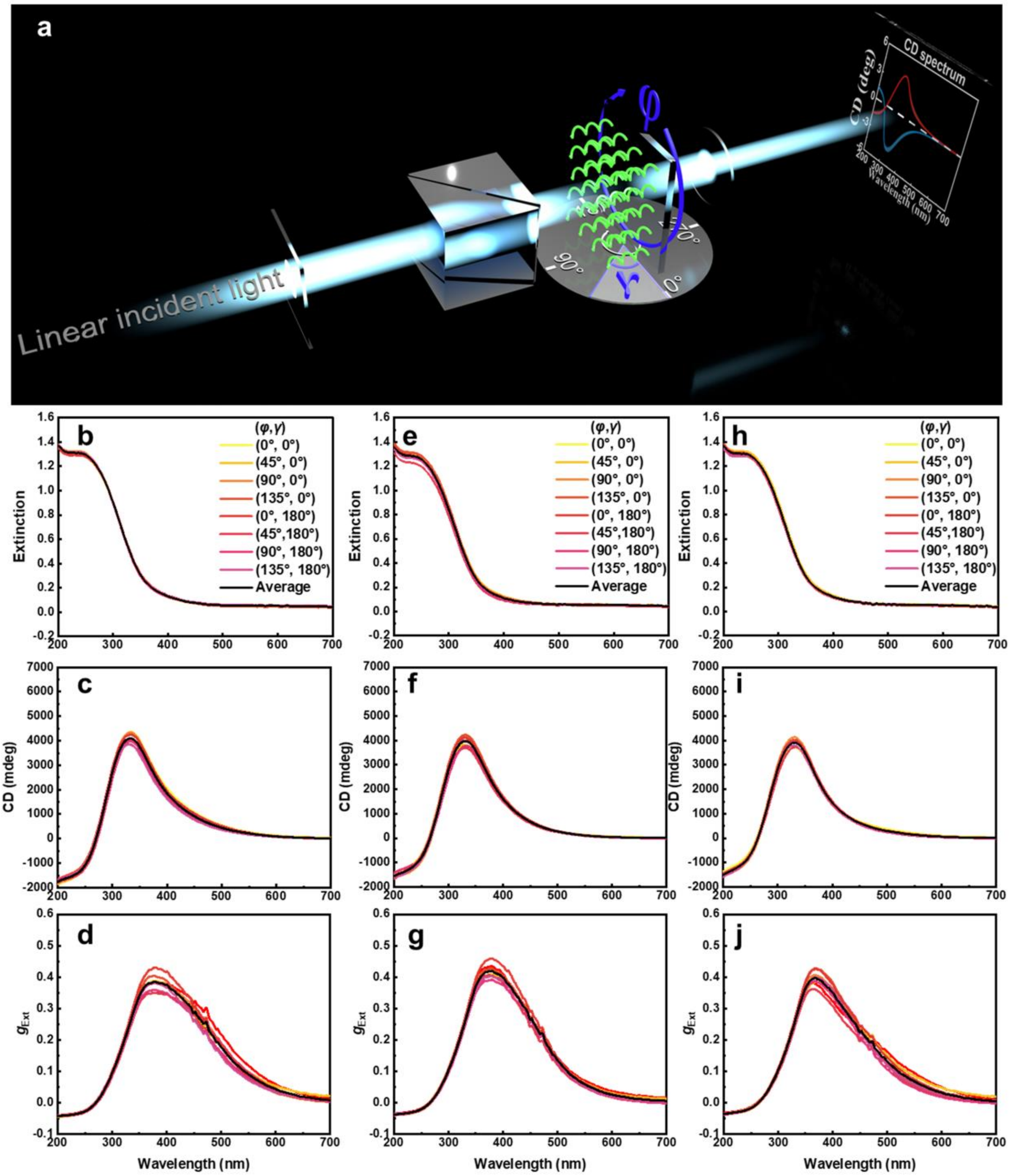

Supplementary Figure 3| Optical characterizations of close-packed arrays of LH-TiO_2 NHs (*P* = 310 nm, *n* = 1). a Schematic of measuring extinction and CD spectra, as a function of polar angle (φ) and azimuth angle (γ). UV-visible spectra of (**b**, **e**, and **h**) extinction, (**c**, **f**, and **i**) CD, and (**d**, **g**, and **j**) anisotropic *g*-factor (g_{Ext}), recorded at (φ, γ) = (0°, 0°), (45°, 0°), (90°, 0°), (135°, 0°), (0°, 180°), (45°, 180°), (90°, 180°), and (135°, 180°). Eight spectra were then algebraically averaged (marked as black spectra) to eliminate linear birefringence and linear dichroism. (**b**-**d**, **e**-**g**, and **h**-**j**) Three samples were monitored for obtaining mean spectra with standard deviation, as shown in **Fig. 3p-r**.

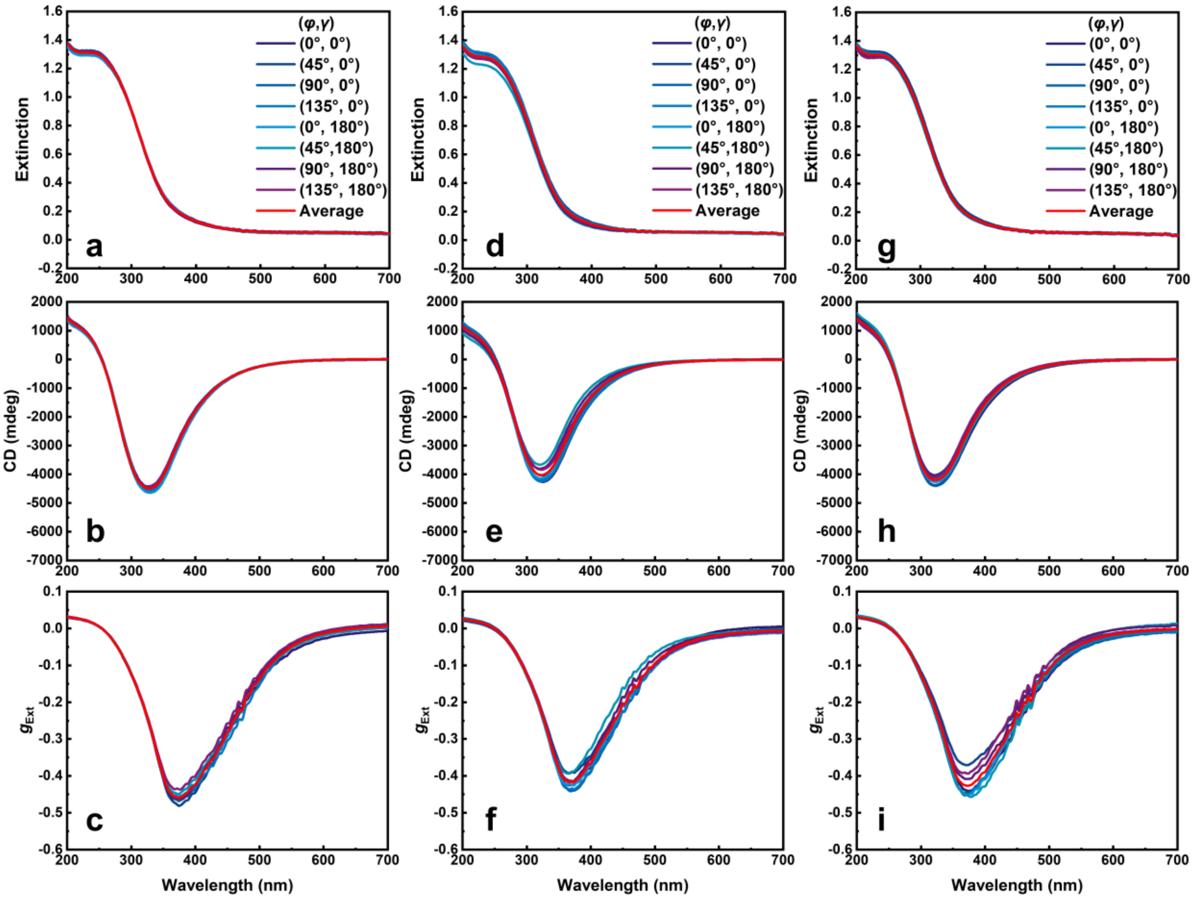

Supplementary Figure 4| Optical characterizations of RH-TiO_2 NHs (*P* = 300 nm, *n* = 1). UV-visible spectra of (**a**, **d**, and **g**) extinction, (**b**, **e**, and **h**) CD, and (**c**, **f**, and **i**) g_{Ext} recorded at (φ, γ) = (0°, 0°), (45°, 0°), (90°, 0°), (135°, 0°), (0°, 180°), (45°, 180°), (90°, 180°), and (135°, 180°). Eight spectra were then algebraically averaged (marked as red spectra) to eliminate linear birefringence and linear dichroism. (**a**-**c**, **d**-**f**, and **g**-**i**) Three samples were monitored for obtaining mean spectra with standard deviation, as shown in **Fig. 3p-r**.

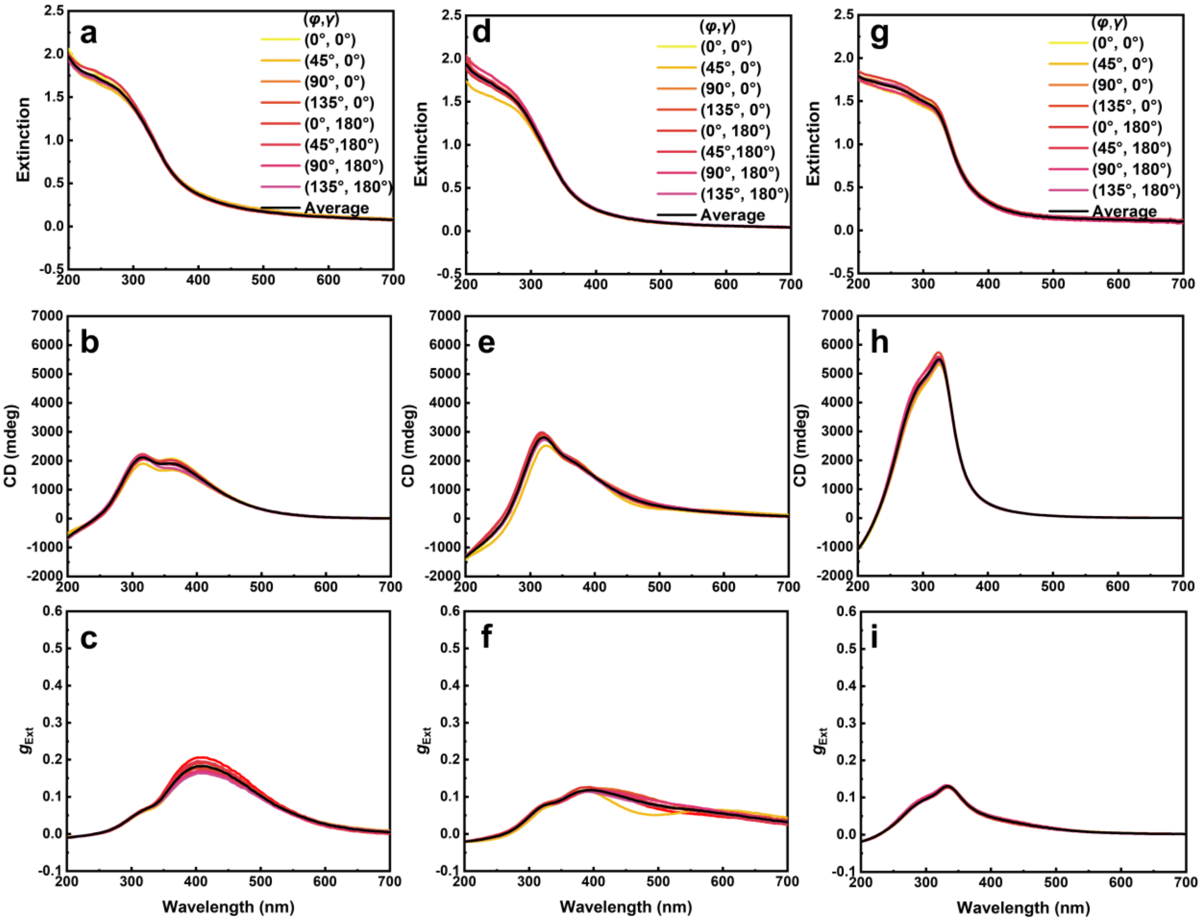

Supplementary Figure 5| Optical characterizations of LH-TiO_2 NHs@ $CsPbBr_3/Cs_4PbBr_6$ (TiO_2 NHs: *P* = 315 nm, *n* = 1; shell coating: [CsBr] = [$PbBr_2$] = 4 mmol L^{-1}, and t_s = 5 h). UV-visible spectra of (**a**, **d**, and **g**) extinction, (**b**, **e**, and **h**) CD, and (**c**, **f**, and **i**) g_{Ext}, recorded at (φ, γ) = (0°, 0°), (45°, 0°), (90°, 0°), (135°, 0°), (0°, 180°), (45°, 180°), (90°, 180°), and (135°, 180°). Eight spectra were then algebraically averaged (marked as black spectra) to eliminate linear birefringence and linear dichroism. (**a**-**c**, **d**-**f**, and **g**-**i**) Three samples were monitored for obtaining mean spectra with standard deviation, as shown in **Fig. 3p-r**.

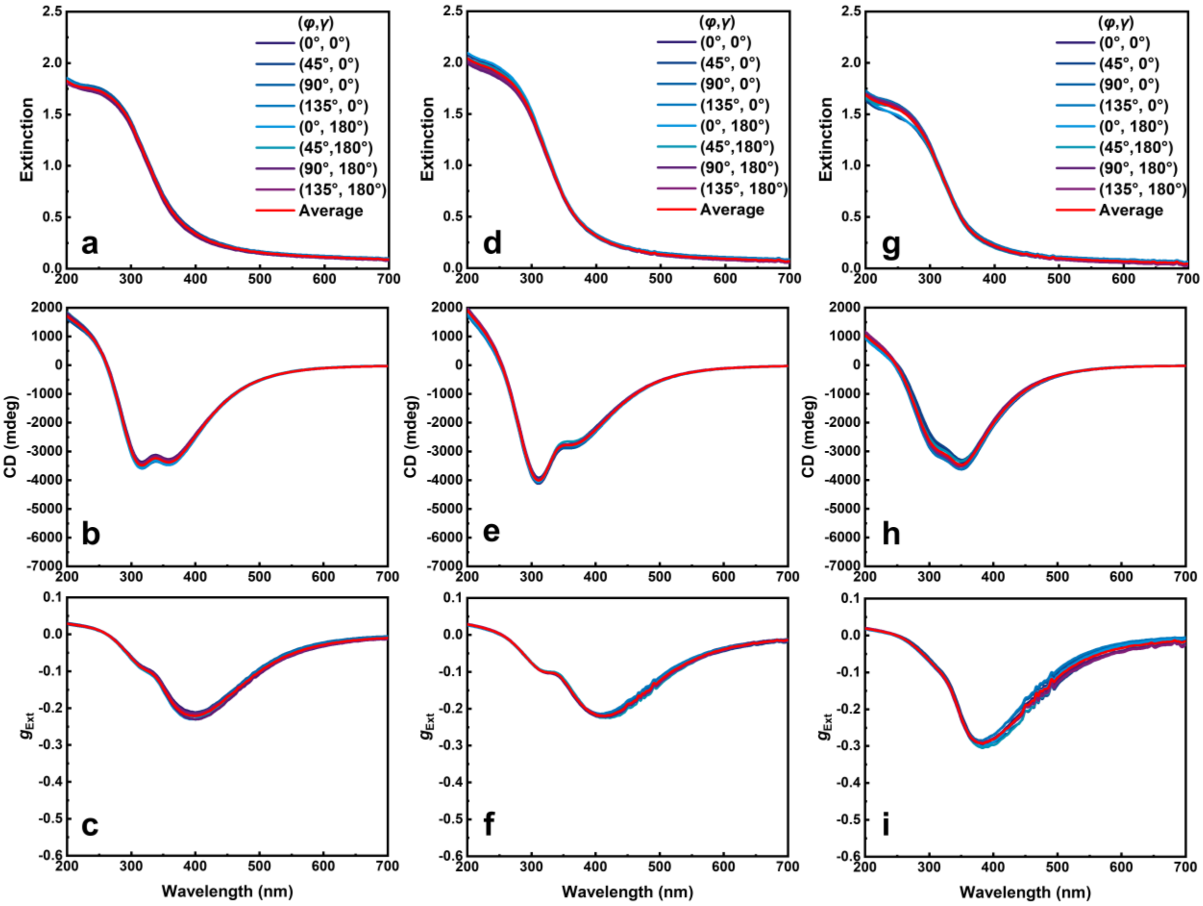

Supplementary Figure 6| Optical characterizations of RH-TiO_2 NHs@ $CsPbBr_3$/Cs_4PbBr_6 (TiO_2 NHs: *P* = 310 nm, *n* = 1; shell coating: [CsBr] = [$PbBr_2$] = 4 mmol L^{-1}, and t_s = 5 h). UV-visible spectra of (**a**, **d**, and **g**) extinction, (**b**, **e**, and **h**) CD, and (**c**, **f**, and **i**) g_{Ext}, recorded at (φ, γ) of (0°, 0°), (45°, 0°), (90°, 0°), (135°, 0°), (0°, 180°), (45°, 180°), (90°, 180°), and (135°, 180°). Eight spectra were then algebraically averaged (marked as red spectra) to eliminate linear birefringence and linear dichroism. (**a-c**, **d-f**, and **g-i**) Three samples were monitored for obtaining mean spectra with standard deviation, as shown in **Fig. 3p-r**.

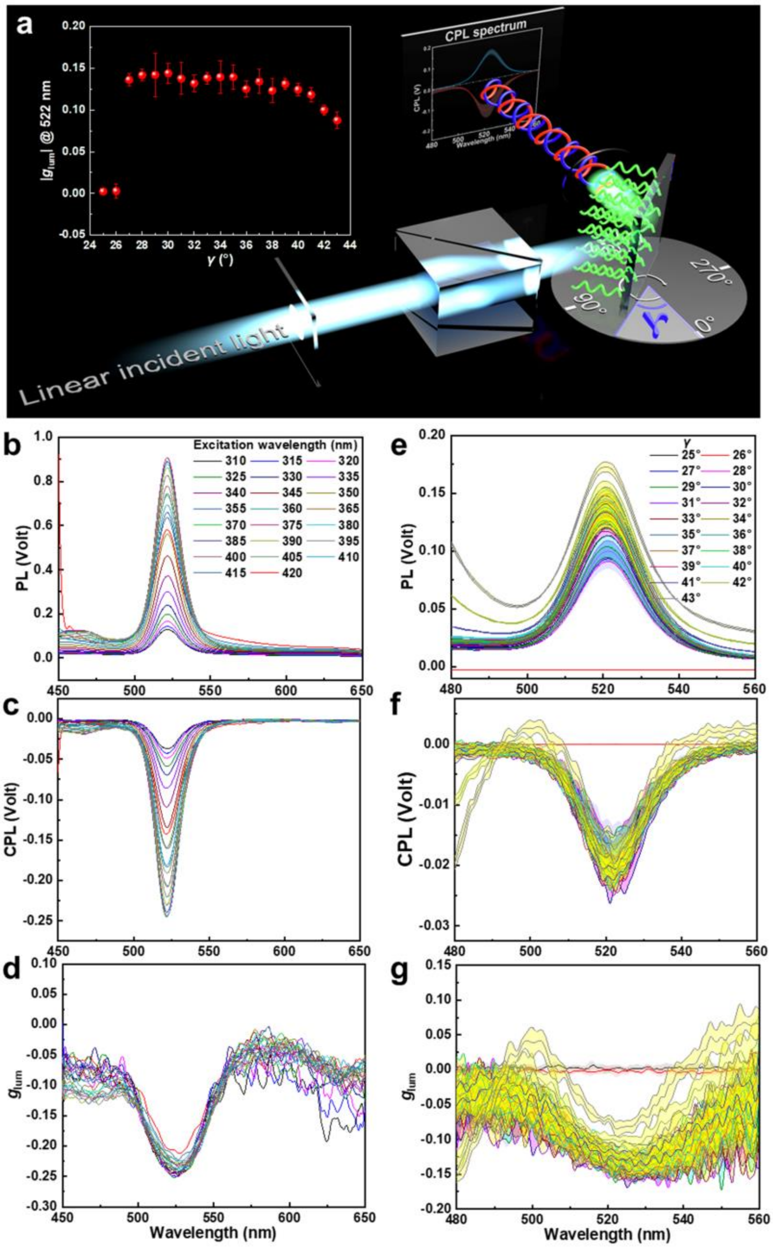

Supplementary Figure 7| Measurement of photo-induced light emission of close-packed arrays of LH-TiO_2 NHs@ $CsPbBr_3$/Cs_4PbBr_6 (TiO_2 NHs: *P* = 315 nm, *n* = 1; shell coating: [CsBr] = [$PbBr_2$] = 4 mmol L^{-1}, and t_s = 5 h). a Schematic of measuring PL and CPL spectra, as a function of polar angle (φ) and azimuth angle (γ). Emission spectra: **b**, **e** PL; **c**, **f** CPL; **d**, **g** g_{lum}. **b-d** Optical measurement was conducted at (φ, γ) = (0°, 30°), and excitation wavelength in a range of 310–420 nm. **e**–**g** Spectra were recorded at γ in a range of 25°– 43° and excitation wavelength of 370 nm. The sample was monitored at φ = 0°, 45°, 90°, 135°, and then four spectra were algebraically averaged and evaluated with standard deviation. Therefore, each spectrum comprises average signals (solid lines) and standard deviation (shaded areas). Plot of mean g_{lum} versus γ is inset in **a**.

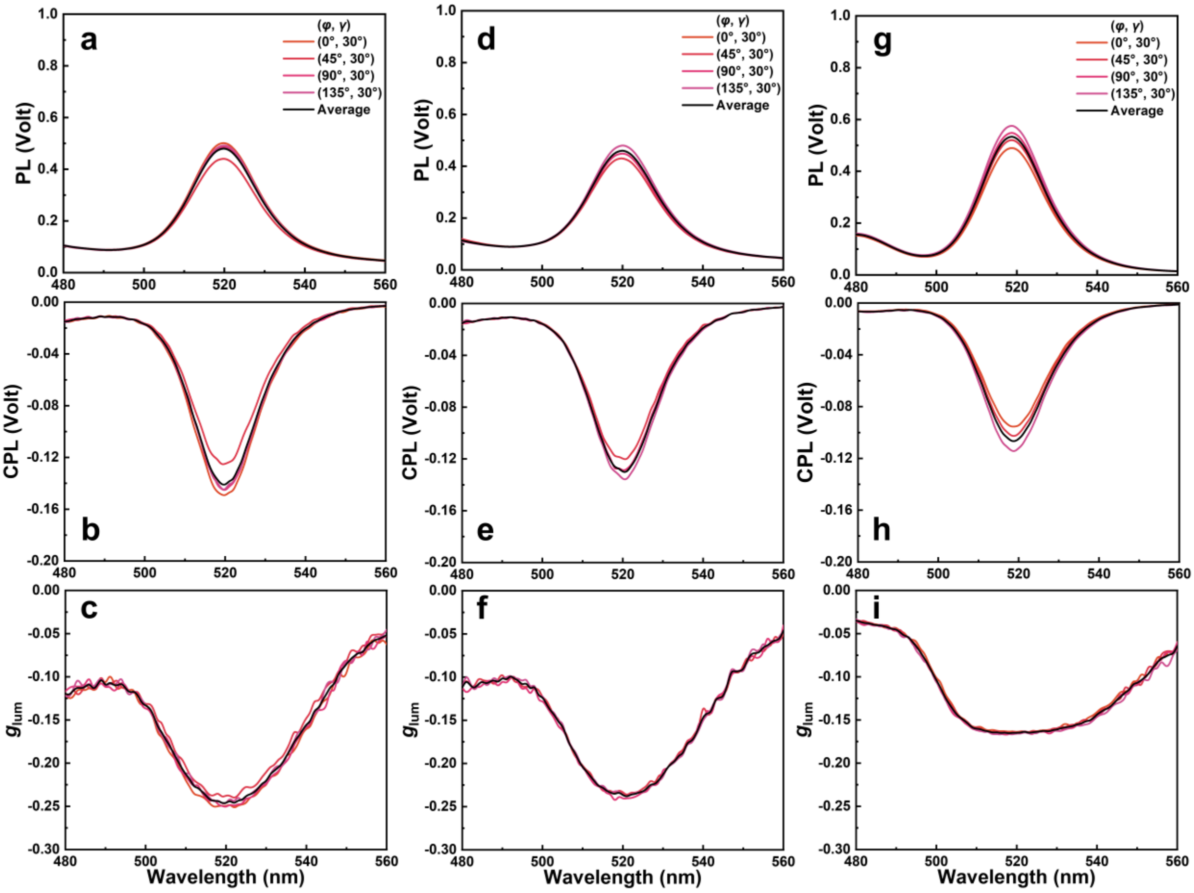

Supplementary Figure 8| Photo-induced light-emission measurement of LH-TiO_2 NHs@$CsPbBr_3$/Cs_4PbBr_6 (TiO_2 NHs: P = 315 nm, n = 1; shell coating: [CsBr] = [$PbBr_2$] = 4 mmol L^{-1}, t_s = 5 h). Emission spectra: **a**, **d**, and **g** PL; **b**, **e**, and **h** CPL; **c**, **f**, and **i** g_{lum}, monitored at an excitation wavelength of 370 nm, $\gamma = 30°$, and $\varphi = 0°, 45°, 90°, 145°$. Four spectra were then algebraically averaged (marked as black spectra) to eliminate linear birefringence and linear dichroism. **a**-**c**, **d**-**f**, and **g**-**i** Three samples were monitored for obtaining mean spectra with standard deviation, as shown in **Fig. 3s-u**.

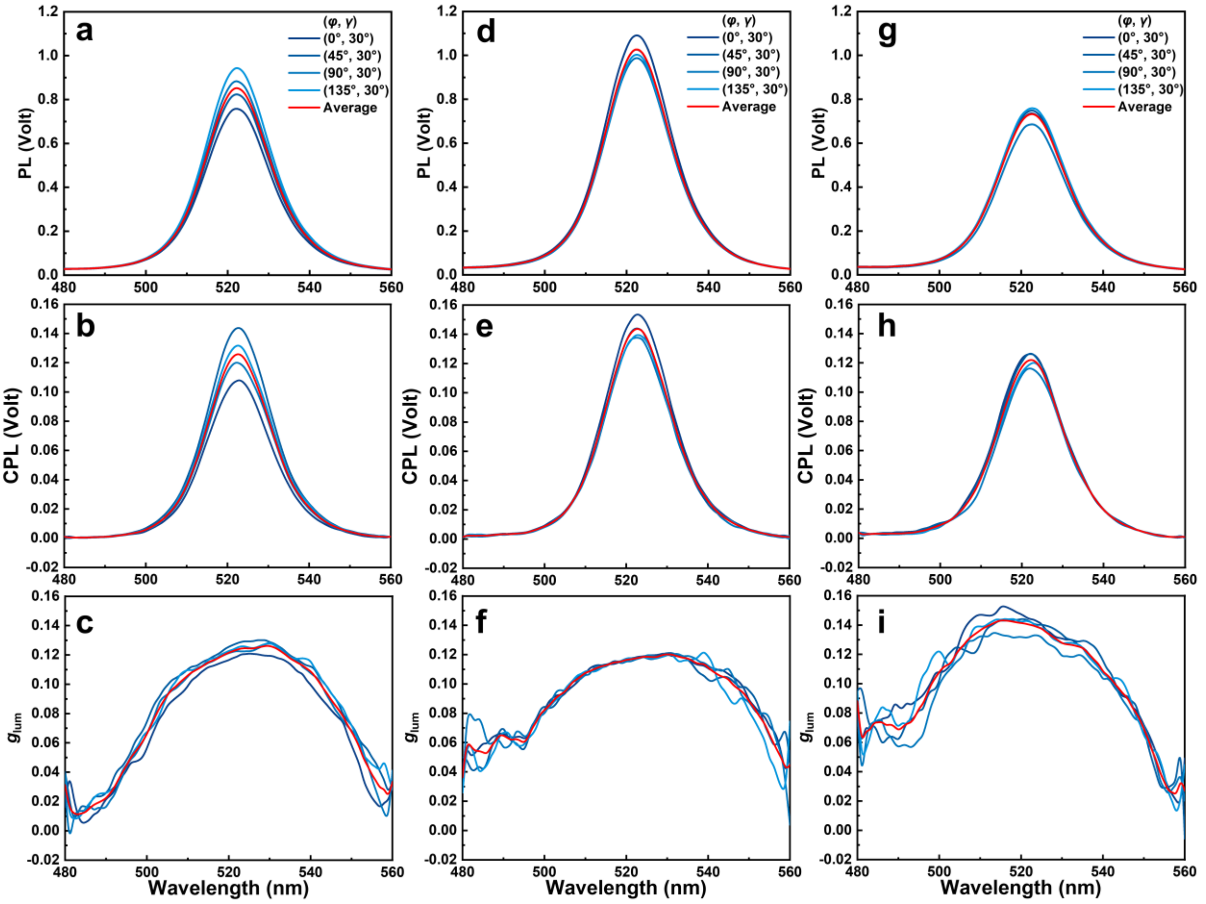

Supplementary Figure 9| Photo-induced Light-emission measurement of RH-TiO_2 NHs@$CsPbBr_3$/Cs_4PbBr_6 (TiO_2 NHs: *P* = 310 nm, *n* = 1; shell coating: [CsBr] = [$PbBr_2$] = 4 mmol L^{-1}, t_s = 5 h). Emission spectra: **a**, **d**, and **g** PL; **b**, **e**, and **h** CPL; **c**, **f**, and **i** g_{lum}, monitored at an excitation wavelength of 370 nm, $\gamma = 30°$, and $\varphi = 0°, 45°, 90°, 145°$. Four spectra were then algebraically averaged (marked as red spectra) to eliminate linear birefringence and linear dichroism. **a-c**, **d-f**, and **g-i** Three samples were monitored for obtaining mean spectra with standard deviation, as shown in **Fig. 2s-u**.

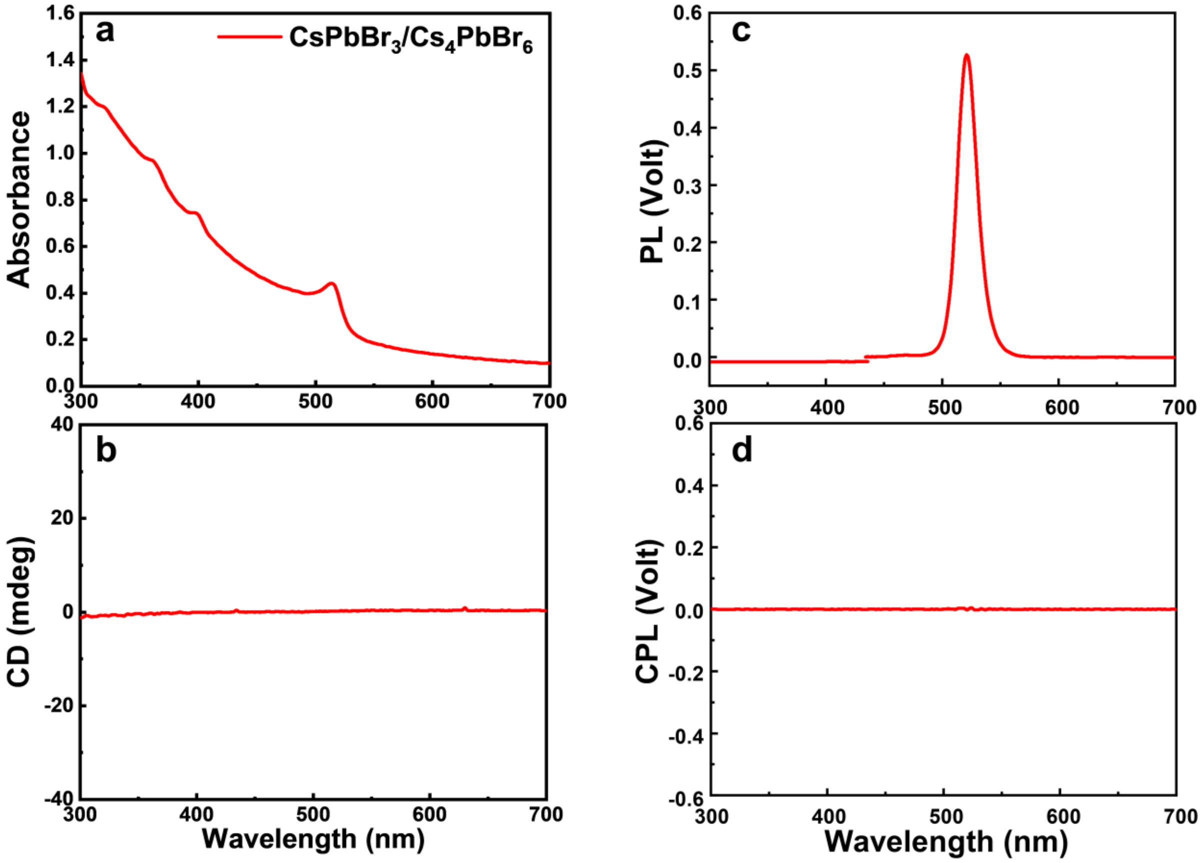

Supplementary Figure 10| Optical characterizations of $CsPbBr_3/Cs_4PbBr_6$ nanocrystals, measured via (**a**) absorption (**b**) CD, (**c**) PL and (**d**) CPL spectroscopies. **c**, **d** Excitation wavelength: 370 nm.

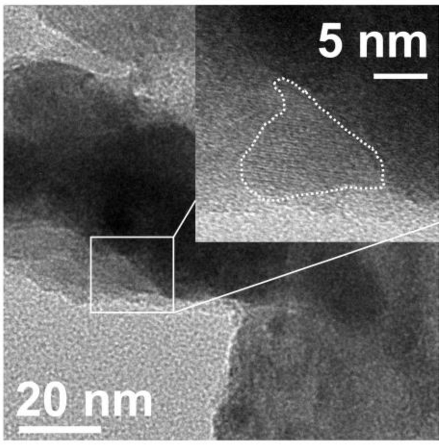

Supplementary Figure 11| High-resolution TEM image of LH-TiO_2 NHs@$CsPbBr_3/Cs_4PbBr_6$.

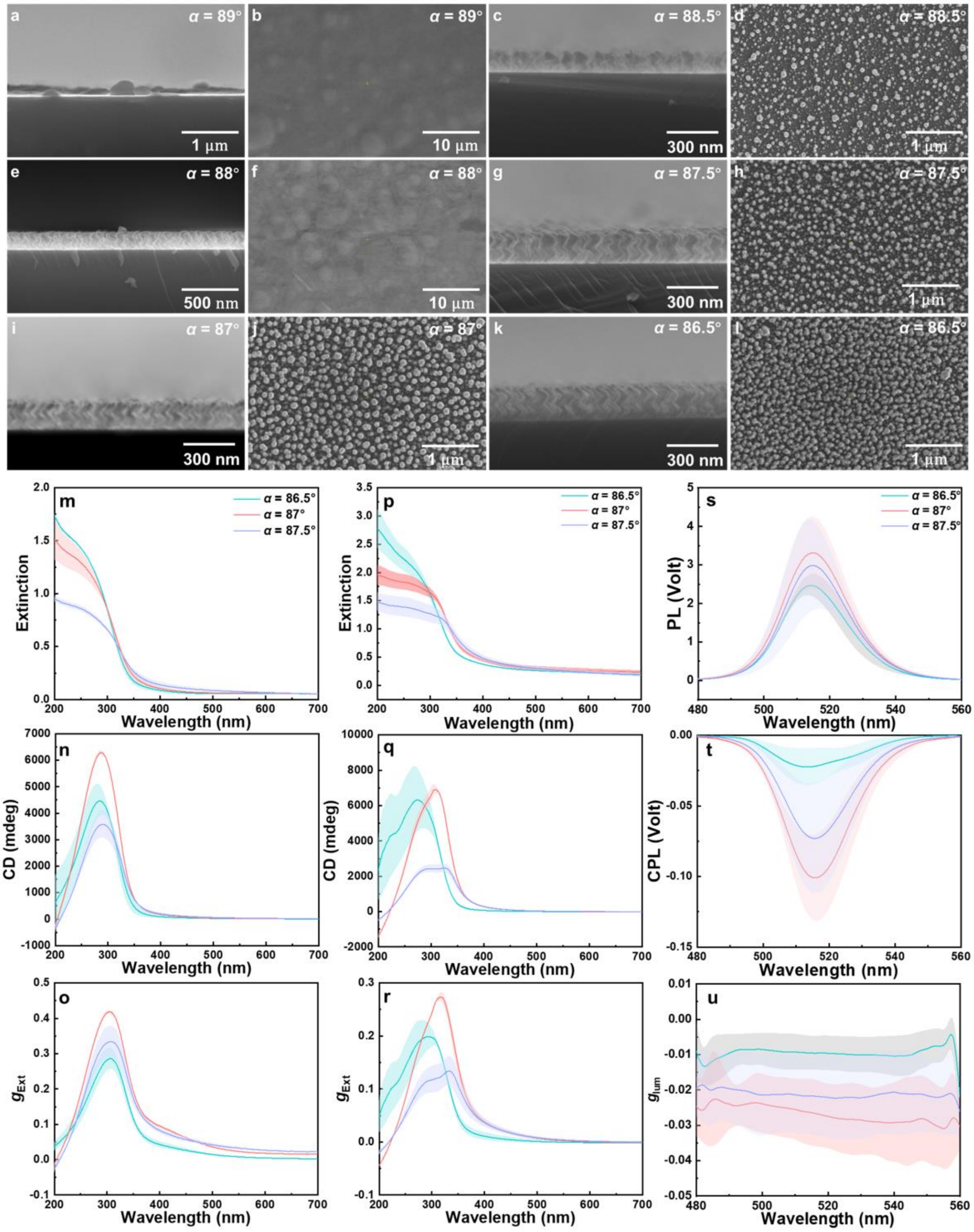

Supplementary Figure 12| Structural and optical characterization of TiO_2 NHs (*P* = 100 nm, and *n* = 3) deposited at controlled *α* and the TiO_2 NHs@ $CsPbBr_3/Cs_4PbBr_6$ grown on these chiral cores. The chiral cores were deposited at *α* of (**a**, **b**) 89°, (**c**, **d**) 88.5°, (**e**, **f**) 88°, (**g**, **h**) 87.5°, (**i**, **j**) 87°, and (**k**, **l**) 86.5°. (**a**, **c**, **e**, **g**, **i**, **k**) SEM cross-sectional images; **b**, **d**, **f**, **h**, **j**, and **l** SEM top-down images. **m**, **n**, and **o** LH-core NHs; **p-r**, and **s-u** LH-core@shells. UV–visible spectra: **m**, **p** extinction; **n**, **q** CD; **o**, **r** g_{Ext}. Emission spectra excited at 370 nm: **s** PL; **t** CPL; **u** g_{lum}. Experimental conditions: R_d = 2.5 Å s^{-1}; *P* = 100 nm; *n* = 3; [CsBr] = [$PbBr_2$] = 20 mmol L^{-1}, t_s = 3 h; *β* = 0.39 (*α*=86.5°); 0.33 (*α*=87°); 0.34 (*α*=87.5°).

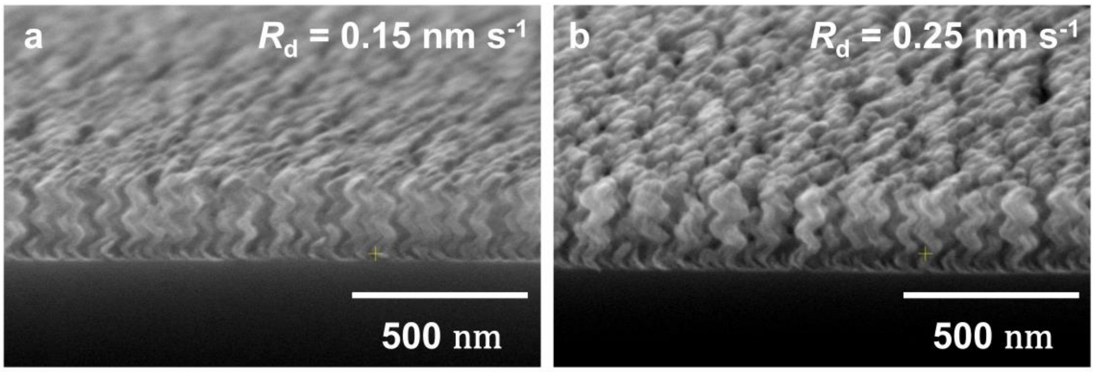

Supplementary Figure 13| Structural characterization of TiO_2 NHs (*P* =100 nm, *n* = 3) deposited at *α* = 87° and engineered deposition rate (R_d). a R_d = 0.15 nm s^{-1}; (b) R_d = 0.25 nm s^{-1}.

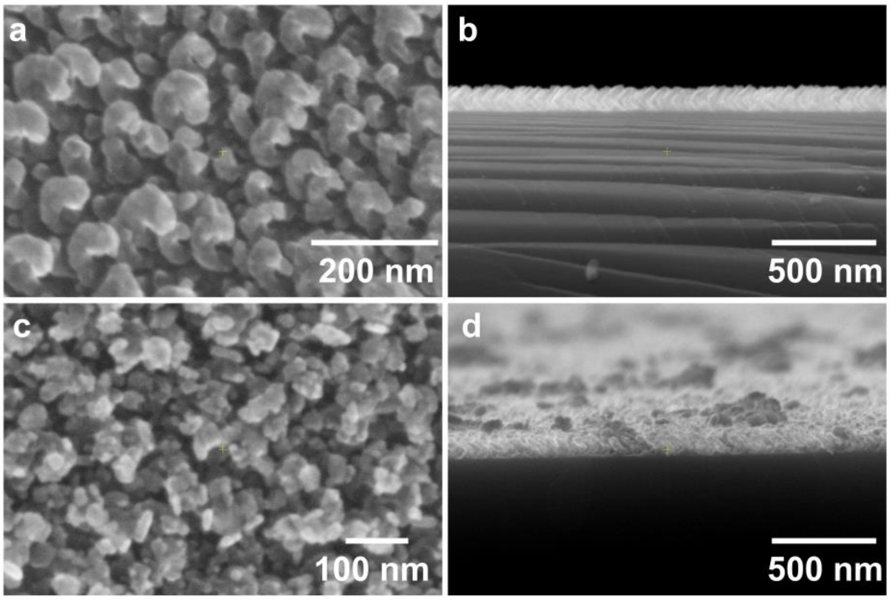

Supplementary Figure 14| Structural characterization of TiO_2 NHs (*P* = 115 nm, *n* = 1) and TiO_2 NHs@$CsPbBr_3$/Cs_4PbBr_6. a, **b** LH-core NHs. **c**, **d** LH-core@shells. **a**, **c** SEM top-down images; **b**, **d** SEM cross-sectional images. Other conditions of forming the core@shells: *α* = 87°; R_d = 0.15 nm s^{-1}; [CsBr] = [$PbBr_2$] = 4 mmol L^{-1}; t_s = 5 h.

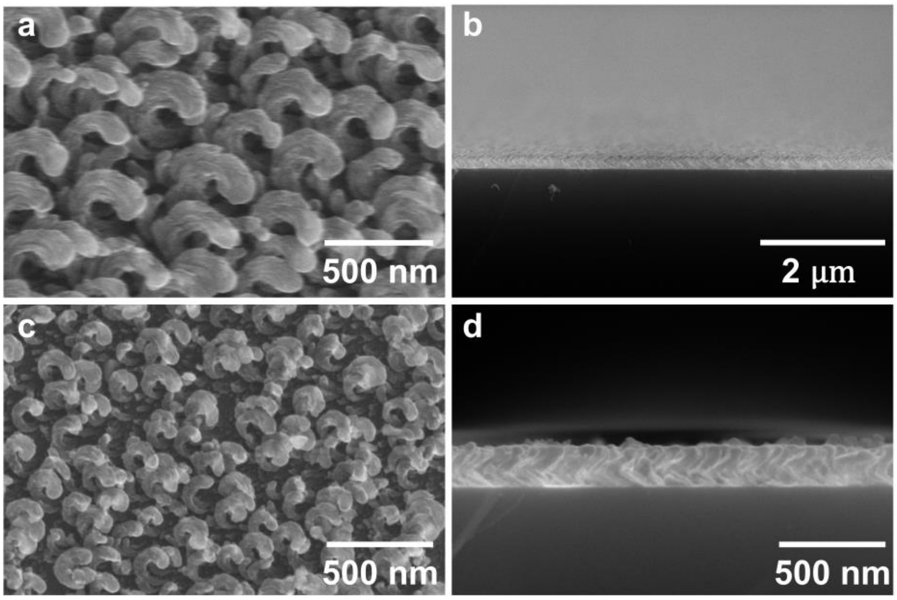

Supplementary Figure 15| Structural characterization of TiO_2 NHs (*P* = 203 nm, *n* = 1) and TiO_2 NHs@$CsPbBr_3$/Cs_4PbBr_6. a, **b** LH-core NHs. **c**, **d** LH-core@shells. **a**, **c** SEM top-down images; **b**, **d** SEM cross-sectional images. Other conditions of forming the core@shells: α = 87°; R_d = 0.15 nm s^{-1}; [CsBr] = [$PbBr_2$] = 4 mmol L^{-1}; t_s = 5 h.

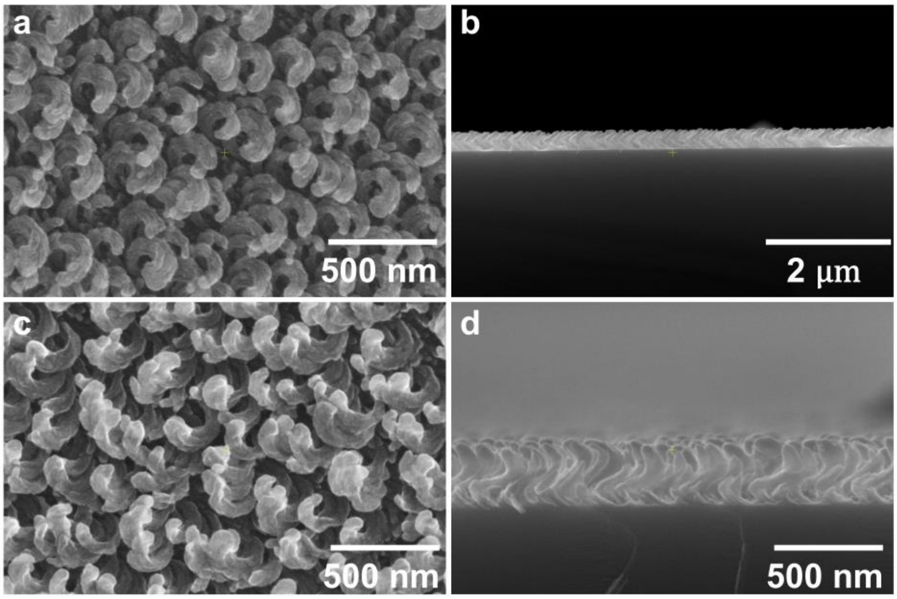

Supplementary Figure 16| Structural characterization of TiO_2 NHs (*P* = 314 nm, *n* = 1) and TiO_2 NHs@$CsPbBr_3$/Cs_4PbBr_6. a, **b** LH-core NHs . **c**, **d** LH-core@shells. **a**, **c** SEM top-down images; **b**, **d** SEM cross-sectional images. Other conditions of forming the core@shells: α = 87°; R_d = 0.15 nm s^{-1}; [CsBr] = [$PbBr_2$] = 4 mmol L^{-1}; t_s = 5 h.

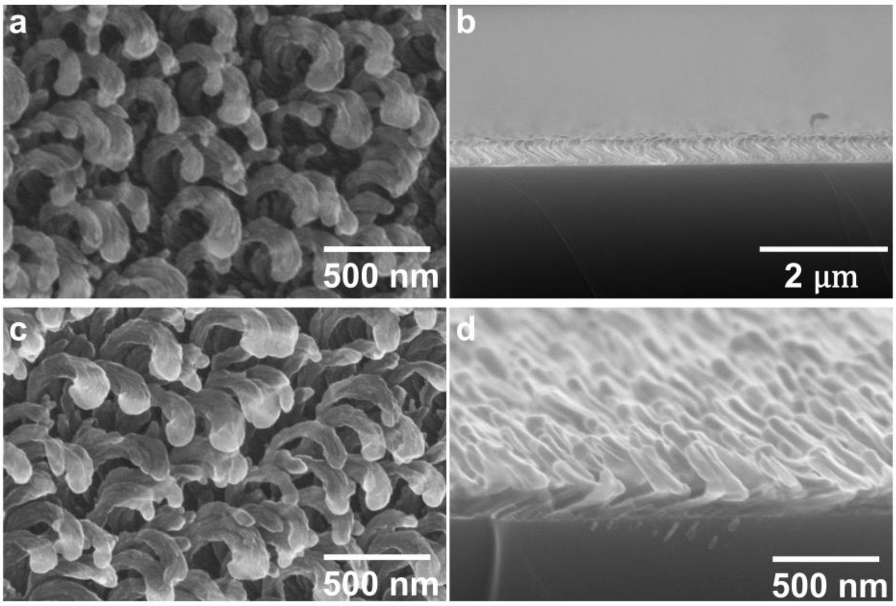

Supplementary Figure 17| Structural characterization of TiO_2 NHs (*P* = 413 nm, *n* = 1) and TiO_2 NHs@$CsPbBr_3$/Cs_4PbBr_6. a, **b** LH-core NHs . **c**, **d** LH-core@shells. **a**, **c** SEM top-down images; **b**, **d** SEM cross-sectional images. Other conditions of forming the core@shells: α = 87°; R_d = 0.15 nm s^{-1}; [CsBr] = [$PbBr_2$] = 4 mmol L^{-1}; t_s = 5 h.

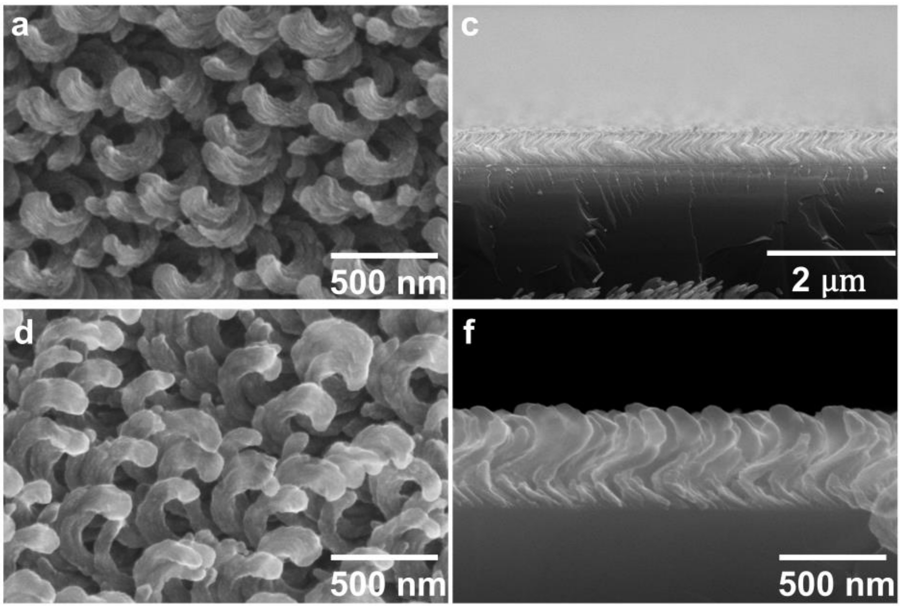

Supplementary Figure 18| Structural characterization of TiO_2 NHs (*P* = 505 nm, *n* = 1) and TiO_2 NHs@$CsPbBr_3$/Cs_4PbBr_6. a, **b** LH-core NHs . **c**, **d** LH-core@shells. **a**, **c** SEM top-down images; **b**, **d** SEM cross-sectional images. Other conditions of forming the core@shells: α = 87°; R_d = 0.15 nm s^{-1}; [CsBr] = [$PbBr_2$] = 4 mmol L^{-1}; t_s = 5 h.

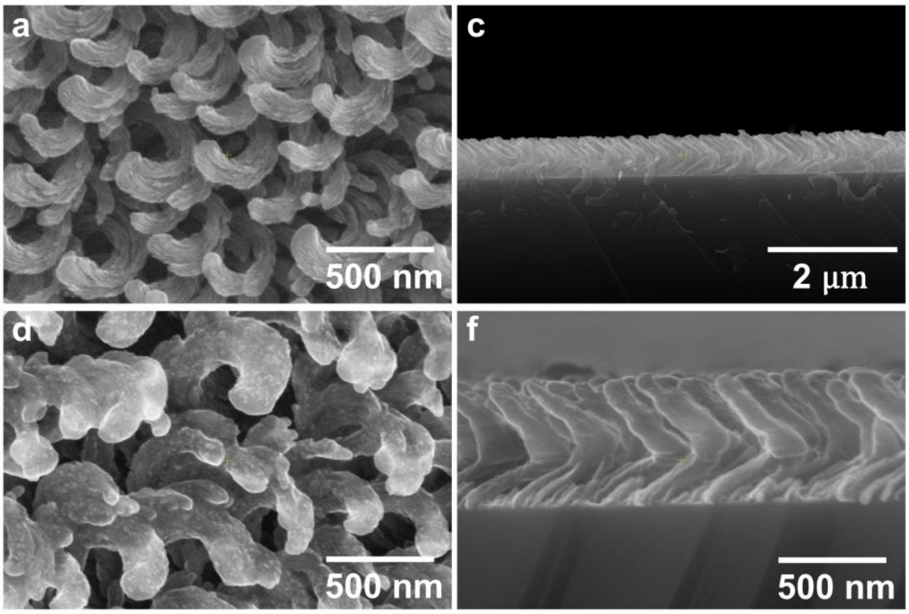

Supplementary Figure 19| Structural characterization of TiO_2 NHs (*P* = 628 nm, *n* = 1) and TiO_2 NHs@$CsPbBr_3$/Cs_4PbBr_6. a, **b** LH-core NHs . **c**, **d** LH-core@shells. **a**, **c** SEM top-down images; **b**, **d** SEM cross-sectional images. Other conditions of forming the core@shells: α = 87°; R_d = 0.15 nm s^{-1}; [CsBr] = [$PbBr_2$] = 4 mmol L^{-1}; t_s = 5 h.

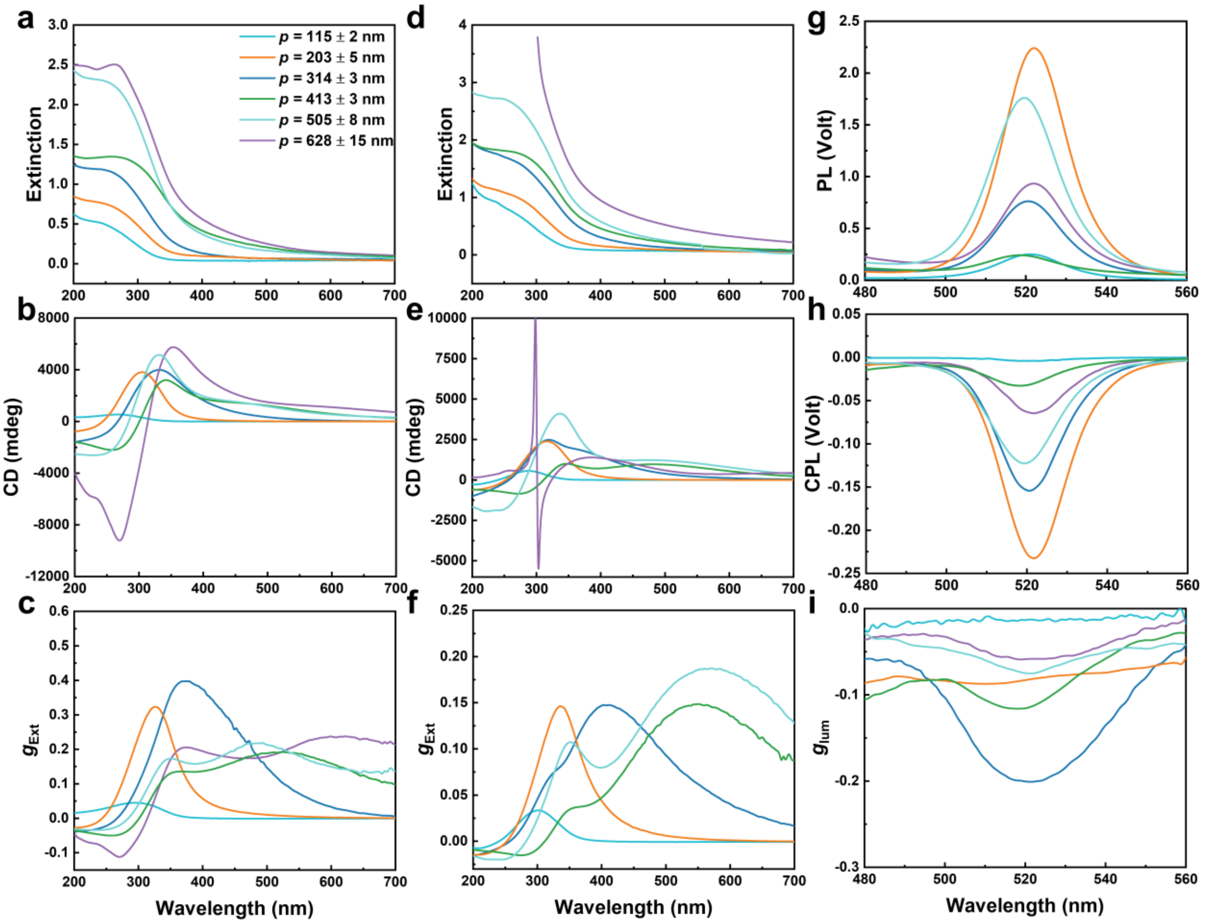

Supplementary Figure 20| Optical characterizations of TiO_2 NHs and TiO_2 NHs@ $CsPbBr_3/Cs_4PbBr_6$, with *P* tuned in a range of 100–650 nm and *n* = 1 (Supplementary Fig. 13-18). a-c LH-core NHs; **d-i** LH-core@shells. UV–visible spectra: **a**, **d** extinction; **b**, **e** CD; **c**, **f** g_{Ext}. Emission spectra excited at 370 nm: **g** PL; **h** CPL; **i** g_{lum}.

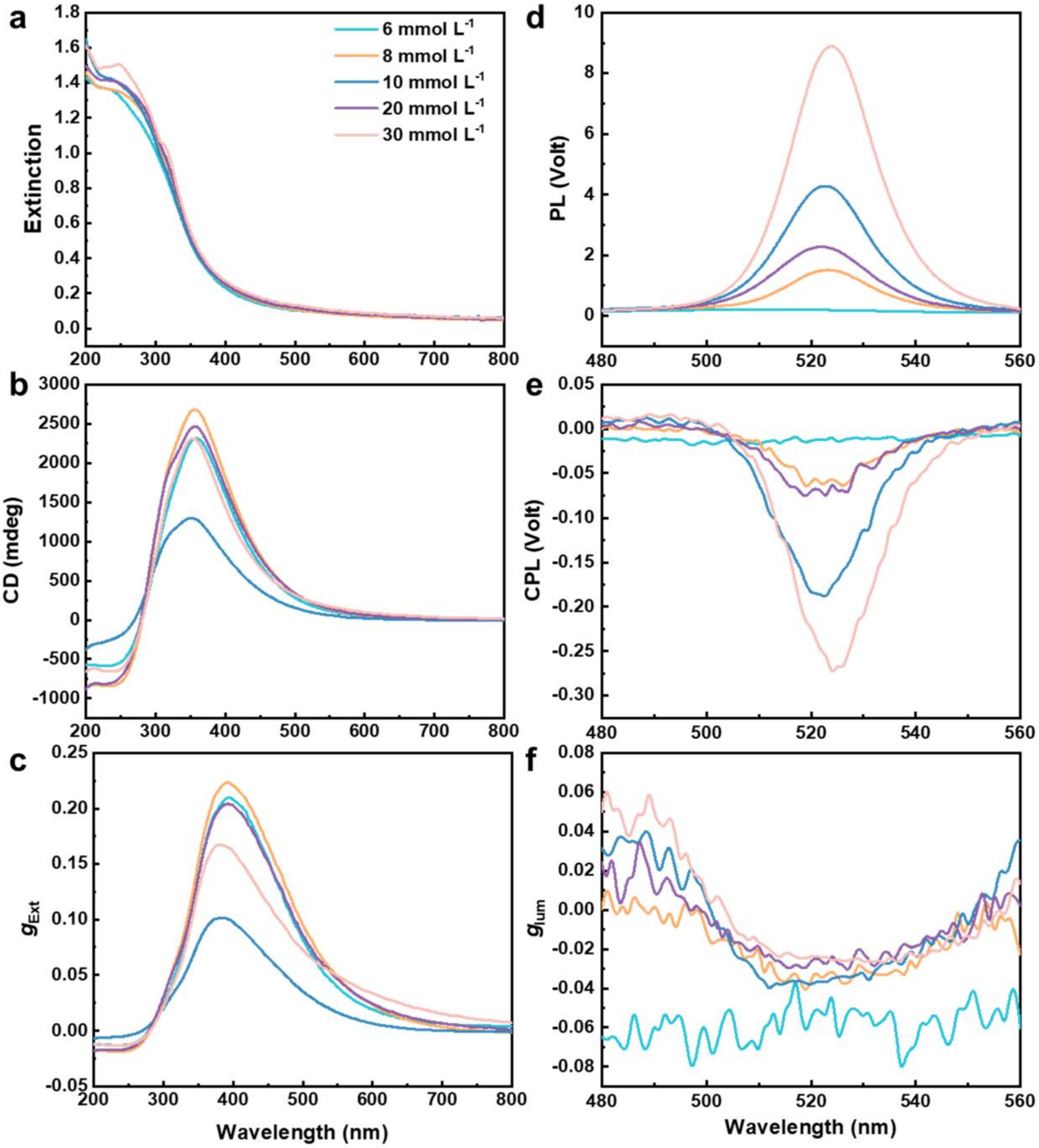

Supplementary Figure 21| Optical characterizations of LH-TiO_2 NHs@ $CsPbBr_3/Cs_4PbBr_6$ (*P* = 314 nm, *n* =1), under shell-coating conditions of [CsBr] = [$PbBr_2$] = 6–30 mmol L^{-1}. UV–visible–NIR spectra: **a** extinction; **b** CD; **c** g_{Ext}. Emission spectra excited at 370 nm: **d** PL; **e** CPL; **f** g_{lum}. Other conditions of forming the core@shells: α = 87°; R_d = 0.15 nm s^{-1}; t_s = 5 h.

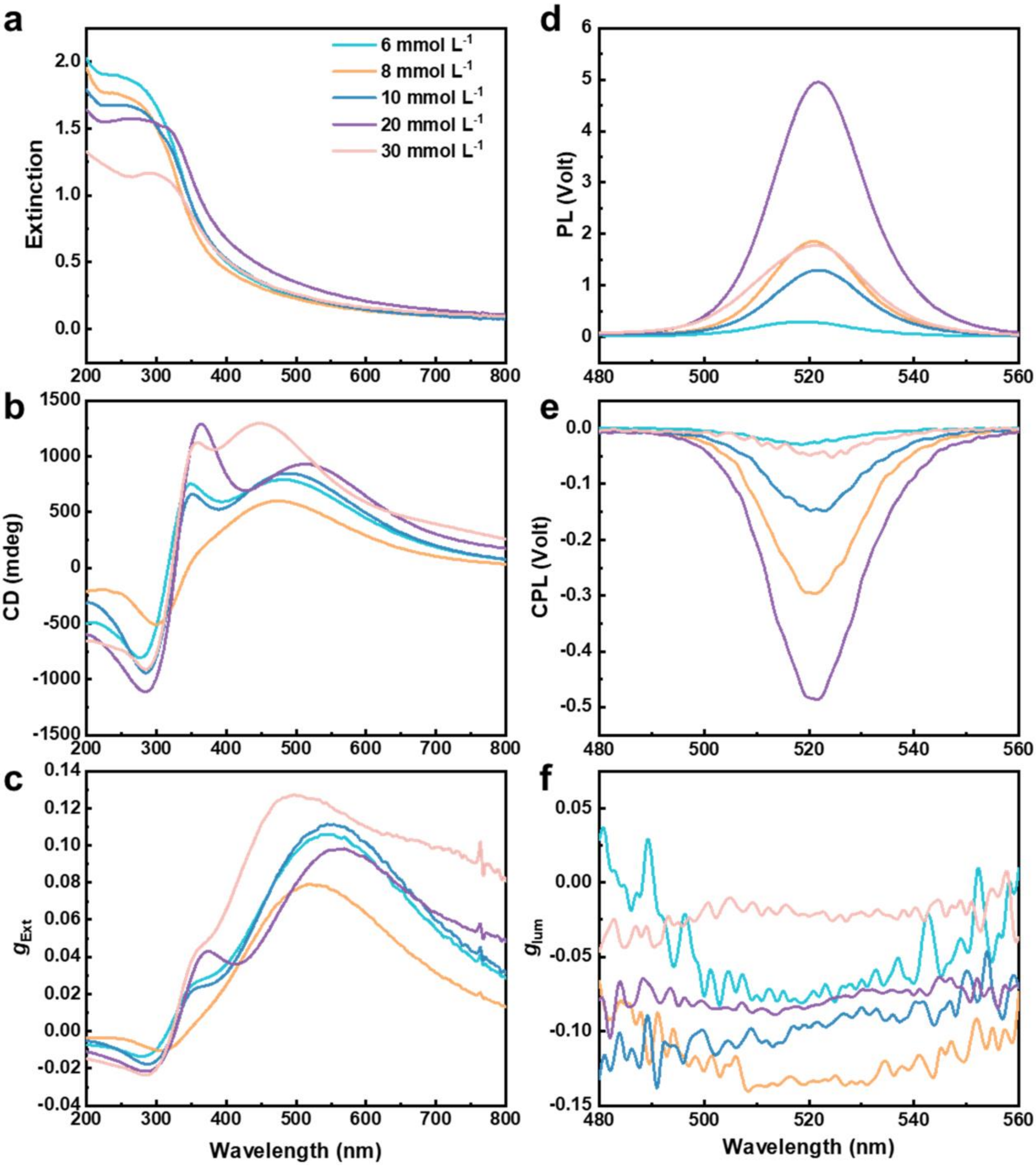

Supplementary Figure 22| Optical characterizations of LH-TiO_2 NHs@ $CsPbBr_3$/Cs_4PbBr_6 (*P* = 413 nm, *n* =1), under shell-coating conditions of [CsBr] = [$PbBr_2$] = 6–30 mmol L^{-1}. UV–visible–NIR spectra: UV–visible–NIR spectra: **a** extinction; **b** CD; **c** g_{Ext}. Emission spectra excited at 370 nm: **d** PL; **e** CPL; **f** g_{lum}. Other conditions of forming the core@shells: α = 87°; R_d = 0.15 nm s^{-1}; t_s = 5 h.

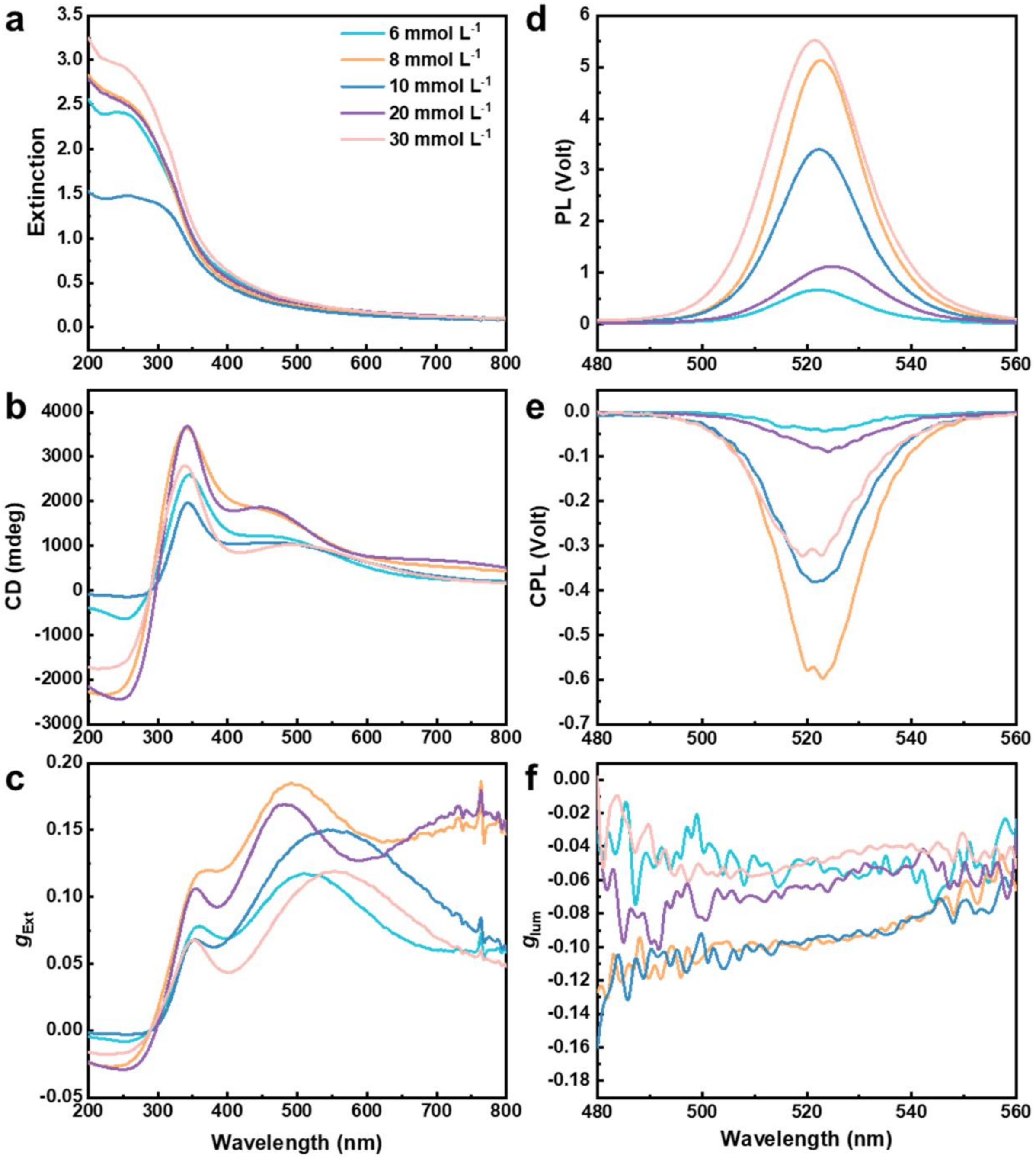

Supplementary Figure 23| Optical characterizations of LH-TiO_2 NHs@ $CsPbBr_3$/Cs_4PbBr_6 (*P* = 505 nm, *n* =1), under shell-coating conditions of [CsBr] = [$PbBr_2$] = 6–30 mmol L^{-1}. UV–visible–NIR spectra: **a** extinction; **b** CD; **c** g_{Ext}. Emission spectra excited at 370 nm: **d** PL; **e** CPL; **f** g_{lum}.Other conditions of forming the core@shells: α = 87°; R_d = 0.15 nm s^{-1}; t_s = 5 h.

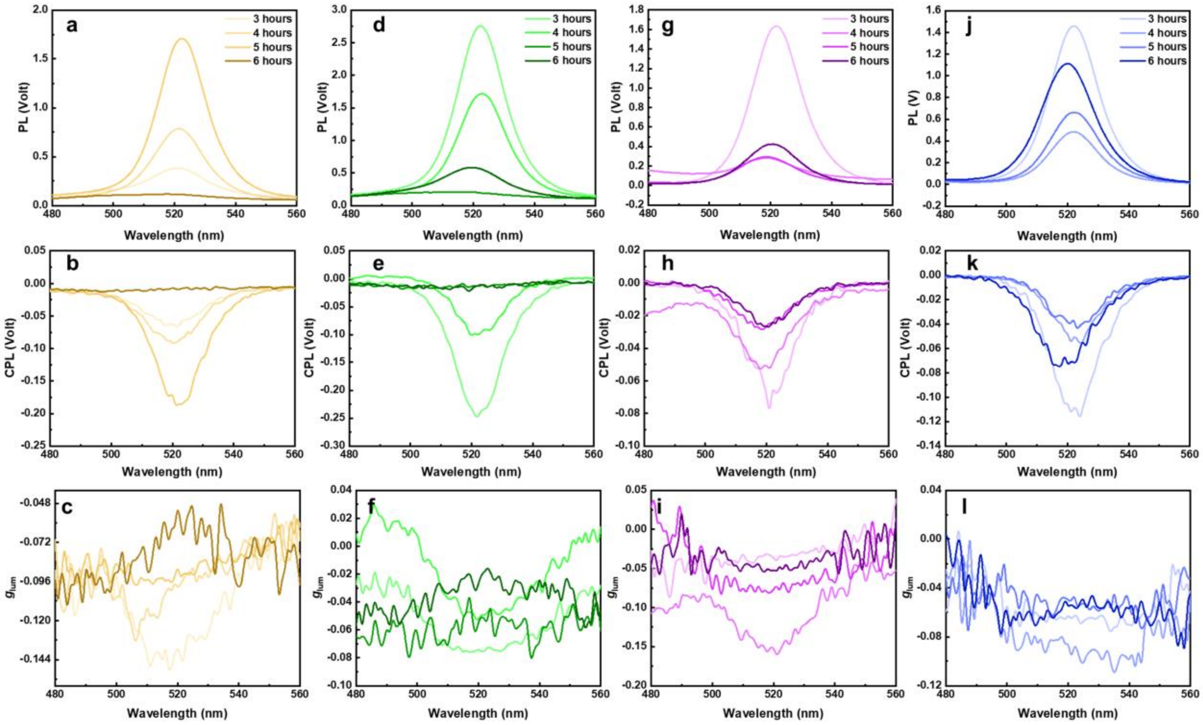

Supplementary Figure 24| Optical characterizations of LH-TiO_2 NHs@ $CsPbBr_3$/Cs_4PbBr_6 (*P* tuned in a range of 200–500 nm, and *n* = 1), formed at t_s = 3–6 h. Emission spectra excited at 370 nm: **a**, **d**, **g**, and **j** PL; **b**, **e**, **h**, and **k** CPL; **c**, **f**, **i**, and **l** g_{lum}. **a**-**c** *P* = 203 nm; **d**-**f** *P* = 314 nm; **g**-**i** *P* = 413 nm; **j**-**l** *P* = 505 nm. Other conditions of forming the core@shells: α = 87°; R_d = 0.15 nm s^{-1}; [CsBr] = [$PbBr_2$] = 6 mmol L^{-1}.

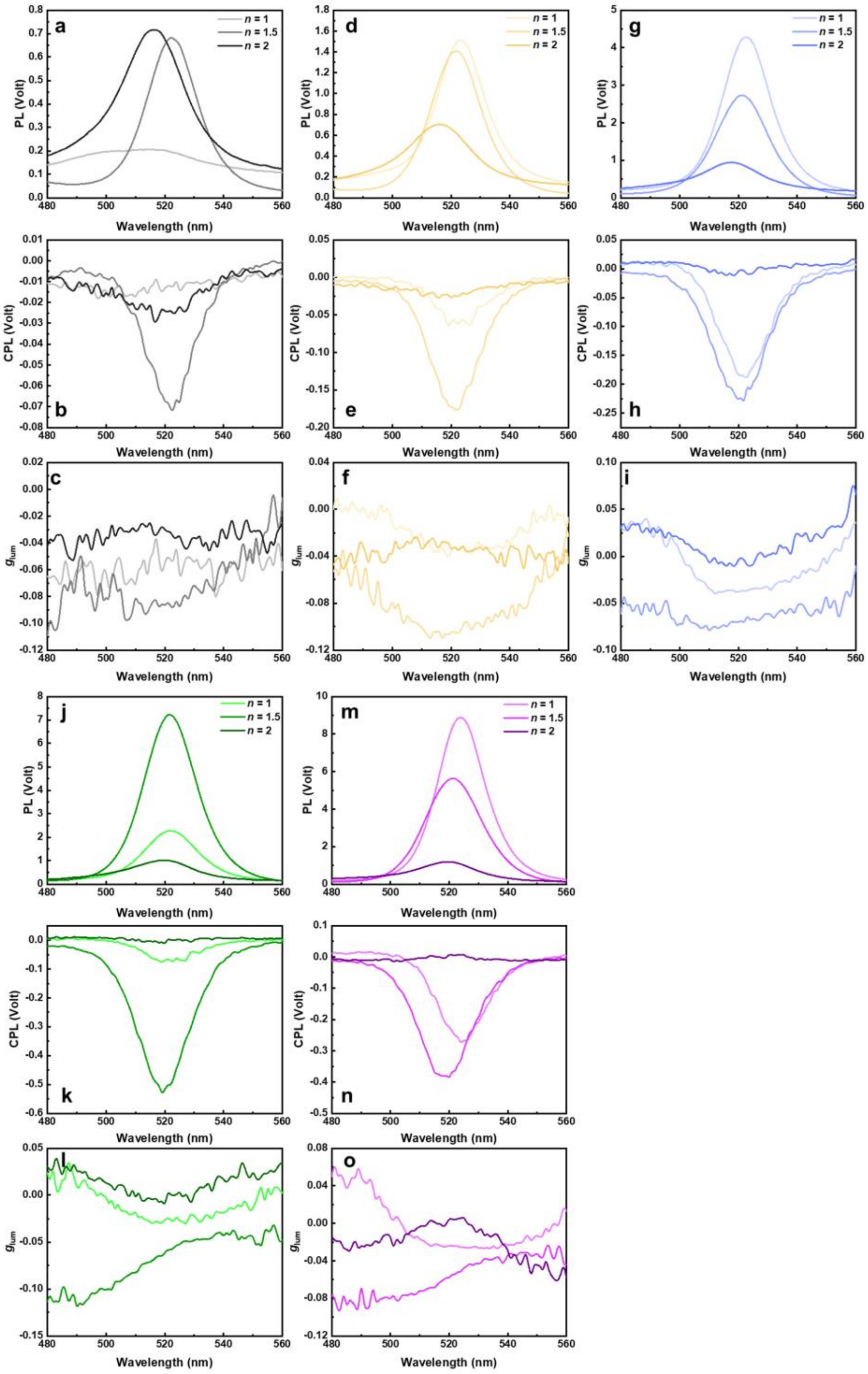

Supplementary Figure 25| Optical characterizations of LH-TiO_2 NHs@ $CsPbBr_3$/Cs_4PbBr_6 (*P* = 314 nm, *n* = 1, 1.5, 2), formed at [CsBr] = [$PbBr_2$] = 6-30 mmol L^{-1}. Emission spectra excited at 370 nm: **a**, **d**, **g**, **j**, and **m** PL; **b**, **e**, **h**, **k**, and **n** CPL; **c**, **f**, **l**, **l**, and **o** g_{lum}. **a-c** [CsBr] = [$PbBr_2$] = 6 mmol L^{-1}; **d-f** [CsBr] = [$PbBr_2$] = 8 mmol L^{-1}; **g-i** [CsBr] = [$PbBr_2$] = 10 mmol L^{-1}; **j-l** [CsBr] = [$PbBr_2$] = 20 mmol L^{-1}; m-o [CsBr] = [$PbBr_2$] = 30 mmol L^{-1}. Other conditions of forming the core@shells: α = 87°; R_d = 0.15 nm s^{-1}; t_s = 5 h.

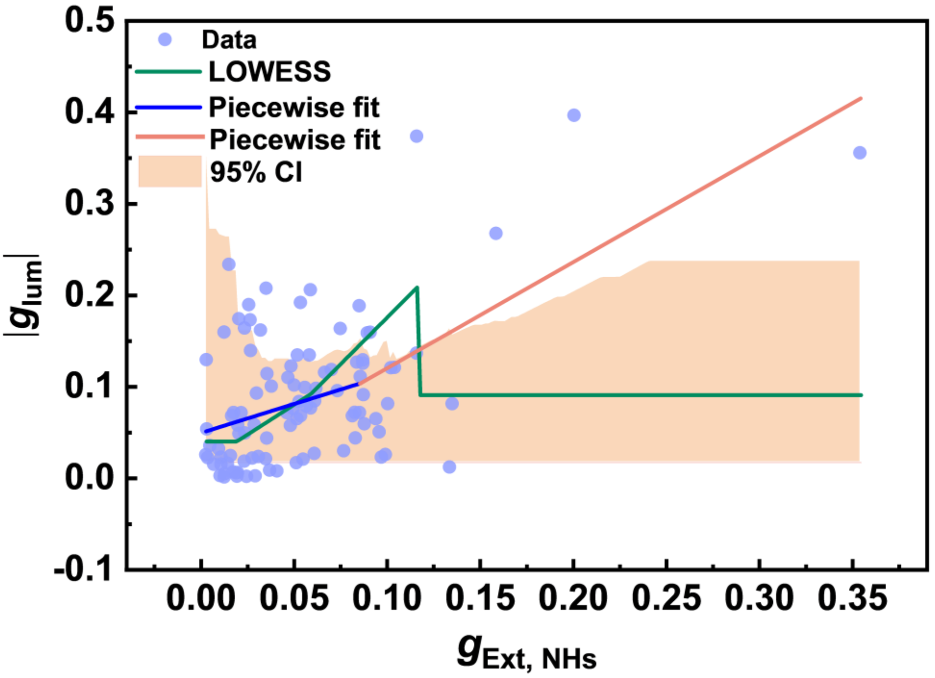

Supplementary Figure 26| Correlation and non-linear analysis between |g_{lum}| and $g_{Ext,NHs}$ of TiO_2 NHs@$CsPbBr_3$/Cs_4PbBr_6. The dark-blue line and orange line represent the piecewise-linear regression, where the optimal breakpoint was selected by minimizing the residual sum of squares within the interior range of $g_{Ext,NHs}$; the two segments remain continuous at the junction. The green line corresponds to the LOWESS non-parametric smoothing (span = 0.3), and the shaded orange shaded region shows its 95% bootstrap confidence interval (CI) (1,000 resamples). Both Pearson (r = 0.555, p = 2.501 × 10^{-9}) and Spearman (ρ = 0.419, p = 1.595 × 10^{-5}) tests indicate a significant positive association, further supported by a distance-correlation coefficient of 0.452 (p = 9.990 × 10^{-4}). The fitted curves collectively reveal an overall near-linear, monotonic positive dependence, with a slight attenuation of slope at intermediate $g_{Ext,NHs}$ values. The number of samples (n) used to execute the statistical analysis is 98. The Pearson correlation coefficient (r) denotes the strength and direction of a linear relationship between two continuous variables, ranging from -1 (perfect negative) to +1 (perfect positive), and r = 0 indicates no linear relationship. p represents the statistical significance, suggesting the chance of observing your results (or more extreme ones) if the null hypothesis (no real effect) were true, which helps us to decide if an experiment's findings are statistically significant. A smaller p values (e.g., <0.05) suggesting stronger evidence against the null hypothesis and towards a real effect. The Spearman rank correlation coefficient (ρ) denotes the strength and direction of a monotonic relationship (not necessarily linear) between two ranked variables, ranging from -1 (perfect negative) to +1 (perfect positive), with 0 indicating no association.

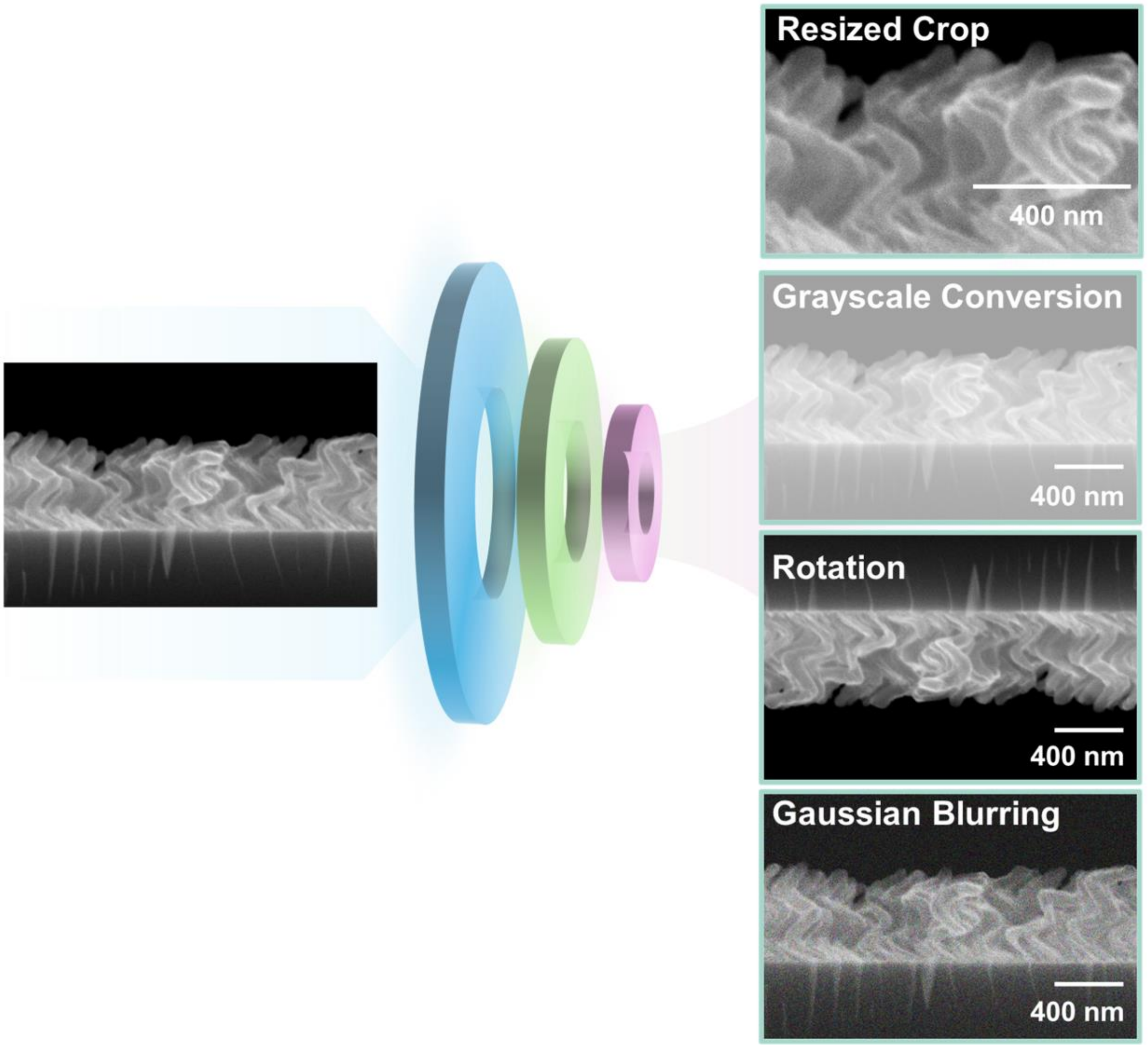

Supplementary Figure 27 | Data augmentation applied in SimCLR. Random resized cropping, grayscale conversion, random rotation and gaussian blurring are implemented for the unsupervised contrastive learning in the morphology modality. The colored circles (blue, green and magenta) schematically indicate the sequential application of data augmentation operations to the input image.

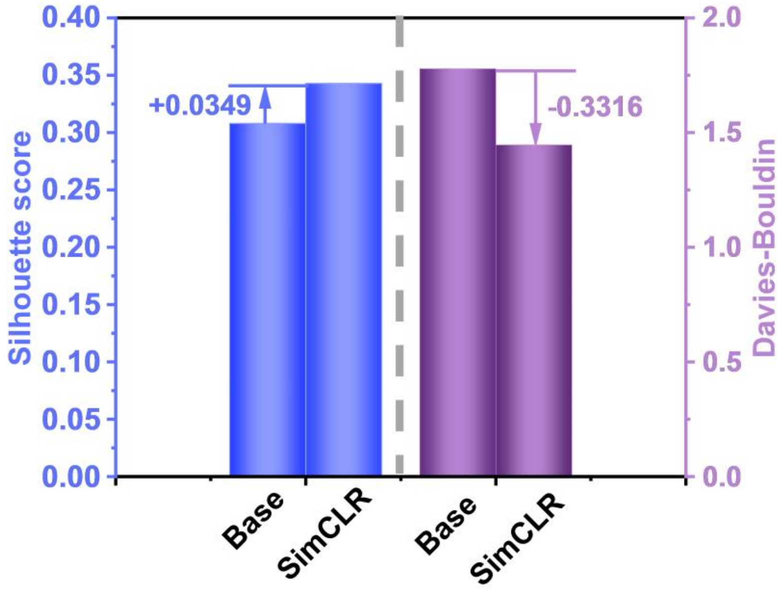

Supplementary Figure 28| Clustering quality comparison of Base to SimCLR. "Base" refers to the model that solely uses ResNet-18 to extract features from SEM images, while "SimCLR" denotes the model enhanced with a rotation-augmented contrastive learning strategy, using ResNet-18 as its backbone. Blue bars show the Silhouette score[S1] (the higher, the better), and purple bars show the Davies–Bouldin index[S2] (the lower, the better). Compared to the Base model, the SimCLR strategy increases the Silhouette score by +0.0349 and reduces the DB index by −0.3316, indicating tighter clusters and clearer inter-cluster separation.

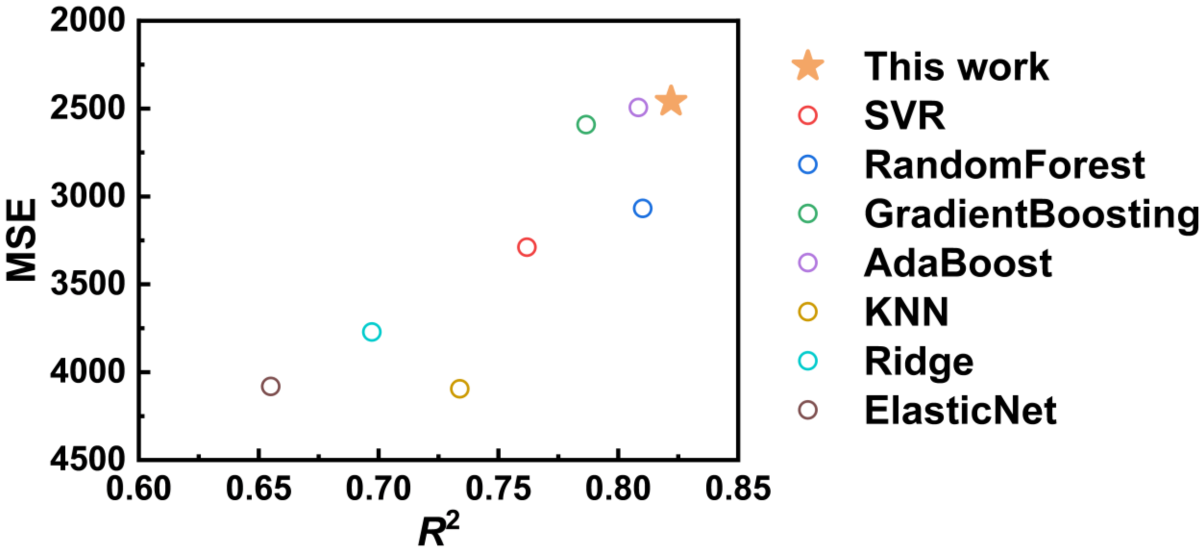

Supplementary Figure 29| Comparison of diverse regressor blocks. Validation performance of candidate regressors includes a scatter of mean squared error (MSE) and the coefficient of determination (R^2). MSE quantifies the average squared deviation between predictions and experimental values, while R^2 measures the proportion of data variance explained by the model. Circles denote classical baselines (SVR,[S3] Random Forest,[S4] Gradient Boosting,[S5] AdaBoost,[S6] KNN,[S7] Ridge,[S8] ElasticNet[S9]). Th orange star marks the result reported in this work (MLP), which achieve the best trade-off (approximately R^2 = 0.82, MSE = 2,503) and is therefore adopted as the regressor used in **Stage 2** (**Figure 5**).

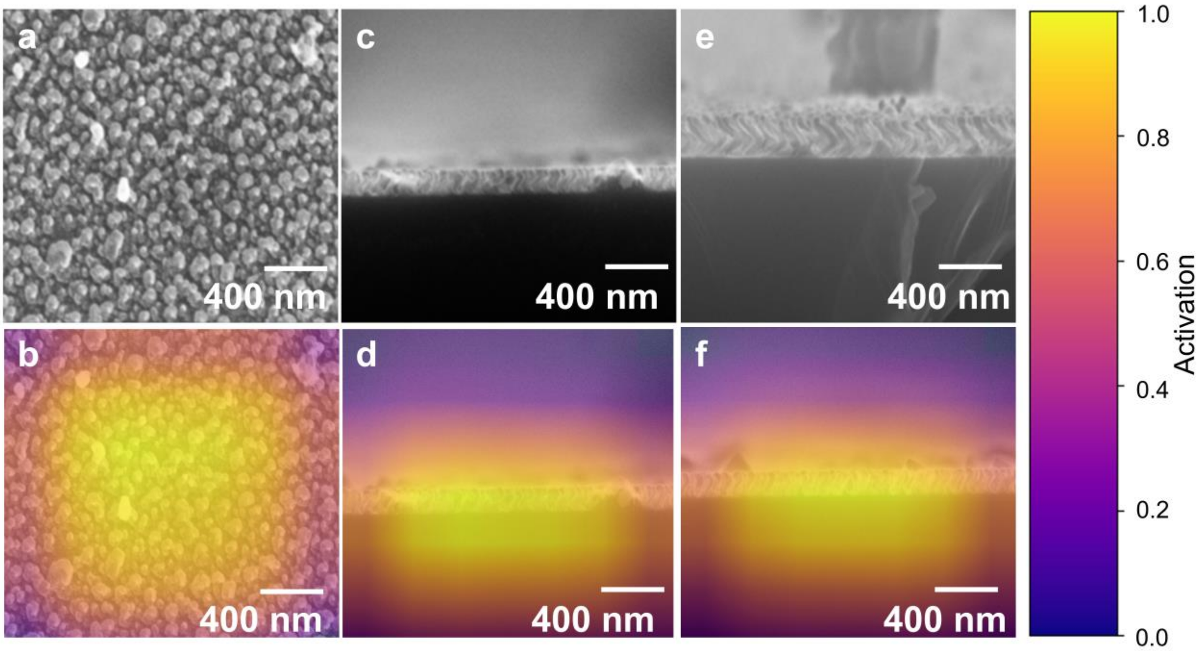

Supplementary Figure 30 | Grad-CAM visualization of morphology modality. a Representative SEM top-view image and **b** Grad-CAM overlay show dominant attribution within structure-bearing regions. **c** Representative SEM cross-sectional image and **d** Grad-CAM overlay (Morphological Module last convolutional block). High activation regions (red) align with the helical layer/pitch-textured region, rather than the substrate/background. **e** Representative SEM tilted image and **f** Grad-CAM overlay (Morphological Module last convolutional block). All SEM images were cropped to remove scale bars and annotations prior to training and attribution. Darker red and blue areas indicate greater and less AI attention, respectively.

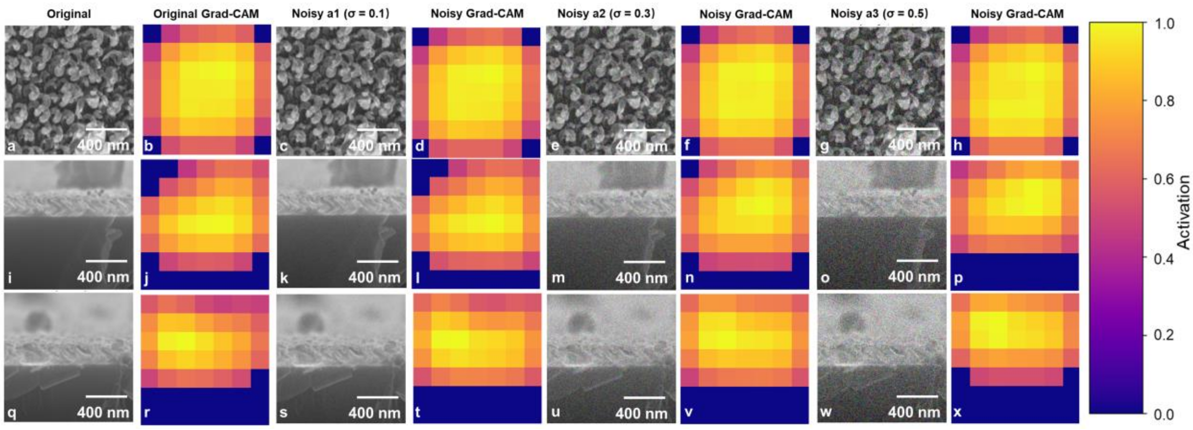

Supplementary Figure 31 | Robustness of Grad-CAM under additive noise perturbations in SEM images. SEM images: **a-h** top-down viewing; **i-p** cross-sectional viewing; **q-x** tilted viewing. **a**, **c**, **e**, **g**, **I**, **k**, **m**, **o**, **q**, **s**, **u** and **w** Original SEM images. (**b**, **d**, **f**, **h**, **j**, **l**, **n**, **p**, **r**, **t**, **v** and **x**) Grad-CAM maps computed from the original SEM images. SEM images via adding Gaussian noise with amplitudes = (**c**, **k**, and **s**) 0.1, (**e**, **m** and **u**) 0.3, (**g**, **o** and **w**) 0.5 (defined on the normalized image-intensity scale), and the corresponding (**b**, **d**, **f**, **h**, **j**, **l**, **n**, **p**, **r**, **t**, **v** and **x**) Grad-CAM maps, respectively. It is found that the Grad-CAM patterns remain highly stable under increasing noise levels: the mean Pearson correlation is 0.992 at 0.1 noise, 0.933 at 0.3 noise, and remains high (0.862) at 0.5 noise. These results indicate that the model's attention is not dominated by noise, and the morphology modality remains robust to SEM perturbations within practical ranges.

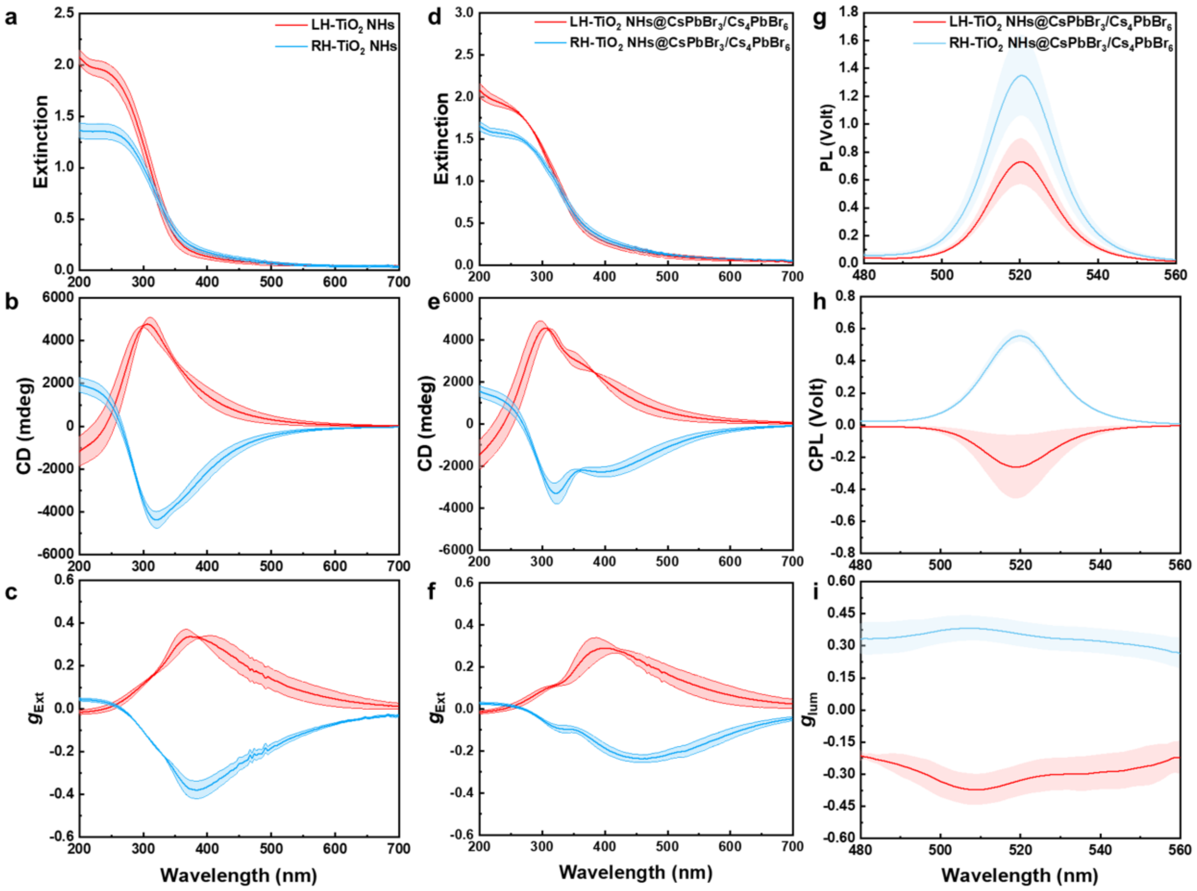

Supplementary Figure 32| Optical characterizations of TiO_2 NHs (with *P* = 395 nm, *n* = 1.07) and TiO_2 NHs@$CsPbBr_3$/Cs_4PbBr_6. The fabrication was performed under the OptiCPL-optimized synthesis conditions: α = 87°, β = 0.33, R_d = 0.15 nm s^{-1}, *P* = 370 nm, *n* = 1.07, [CsBr] = 5.6 mmol L^{-1}, [$PbBr_2$] = 6 mmol L^{-1}, and t_s = 4.9 h. **a-c** Core NHs; **d-i** core@shells. UV–visible spectra: **a**, **d** extinction; **b**, **e** CD; **c**, **f** g_{Ext}. Emission spectra excited at 370 nm: **g** PL; **h** CPL; **i** g_{lum}. LH: red spectra; RH: blue spectra. **a-i** Solid lines: mean values; shaded areas: standard deviations; statistically evaluated from multiple measurements.

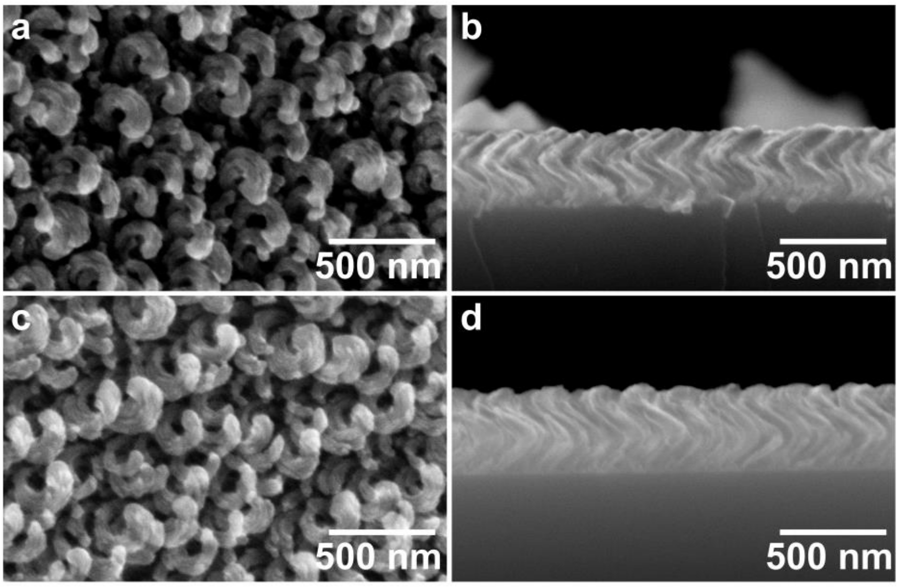

Supplementary Figure 33| Structural characterization of TiO_2 NHs, serving as the chiral templates shown in Supplementary Figure 29. **a**, **b** LH-TiO_2 NHs with *P* = 398 nm; RH-TiO_2 NHs with *P* = 395 nm. **a**, **c** SEM top-down images; **b**, **d** SEM cross-sectional images.

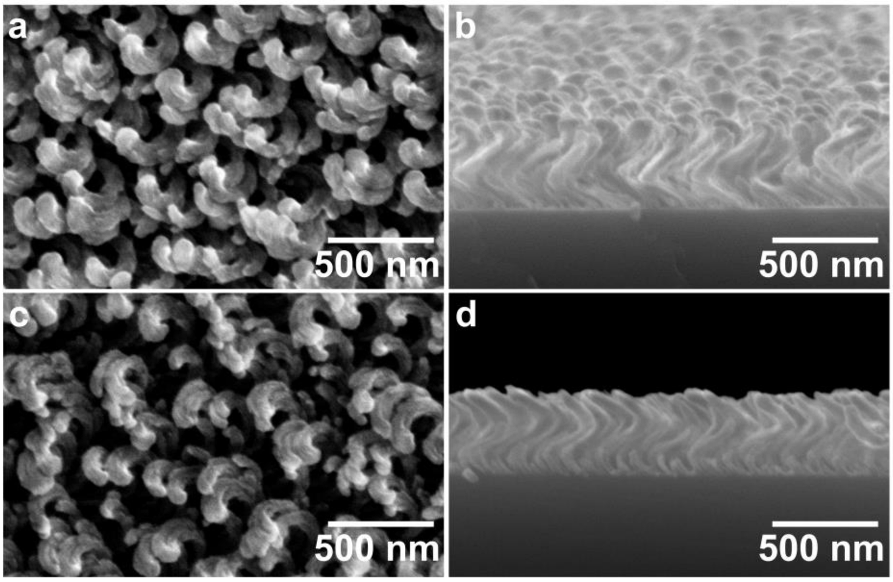

Supplementary Figure 34| Structural characterization of chiral TiO_2 NHs@ $CsPbBr_3$/Cs_4PbBr_6, as shown in Supplementary Figure 29. **a**, **b** LH-core@shells; **c**, **d** RH-core@shells. **a**, **c** SEM top-down images; **b**, **d** SEM cross-sectional images.

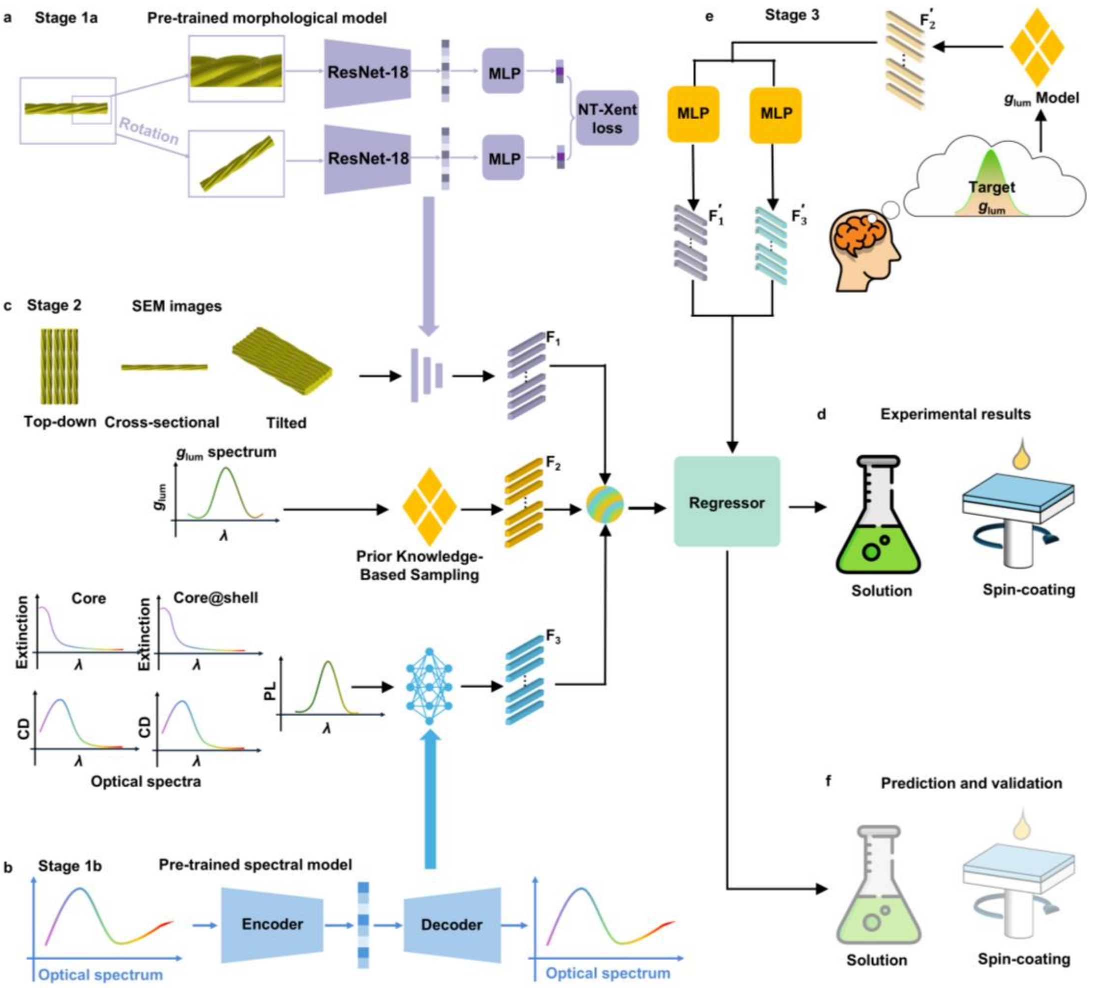

Supplementary Figure 35| The workflow of OptiCPL applied to *R*/*S*-5011/F8BT System. Stage 1 comprises two pre-trained modules: **a** a morphological module, where a pre-trained vision model is fine-tuned on SEM images of *R*/*S*-5011/F8BT with via self-supervised learning, and (**b**) a spectral module, where different types of spectral data are encoded using an autoencoder-based framework. In Stage 2 (**c**), embeddings extracted from SEM images and spectral data are fused with g_{lum} spectra, and the combined representations are used to train a regressor based on experimental result (**d**) for predicting synthesis parameters (concentration of F8BT, concentration of *R*/*S*-5011, annealing temperature, annealing duration, spin-coating rate, spin-coating duration). In Stage 3 (**e**), a g_{lum}-guided model is trained to capture correlations among g_{lum} spectra, morphological features, and spectral features. Given a desired g_{lum} spectrum, the model reconstructs both morphological and spectral embeddings, which are then processed by the regressor to (**f**) predict the optimized set of fabrication parameters, and finally validated by the experiments.

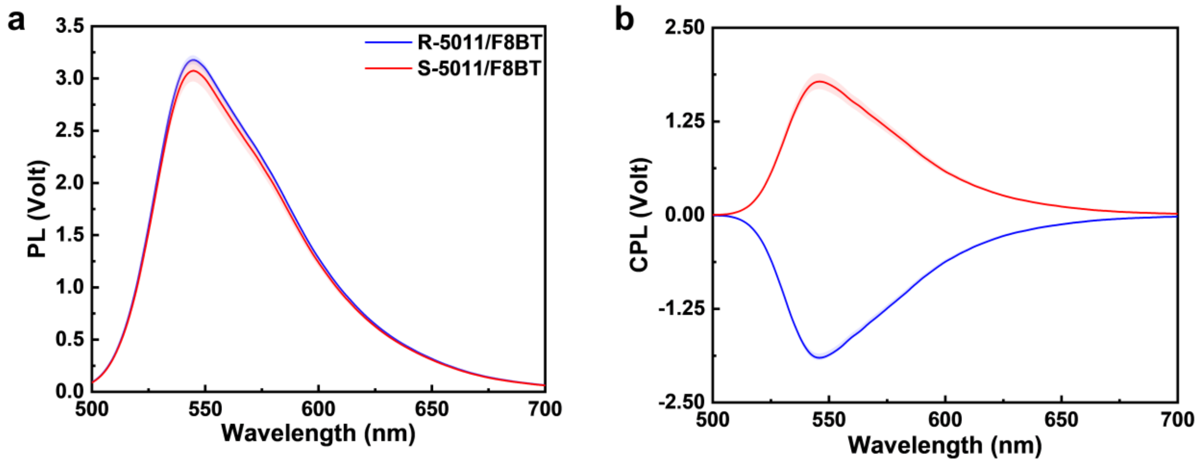

Supplementary Figure 36| Emission spectra of *R/S*-5011/F8BT, excited at 330 nm. **a** PL; **b** CPL. LH: red spectra; RH-blue spectra. **a**, **b** Solid lines: mean values; shaded areas: standard deviations; statistically evaluated from multiple measurements. Fabrication conditions: [F8BT] = 18 mg mL^{-1}; [*R/S*-5011] = 12 mg mL^{-1}; annealing temperature = 200 ℃; annealing duration = 10 min; spin-coating rate = 2,300 rpm; and spin-coating duration = 60 s.

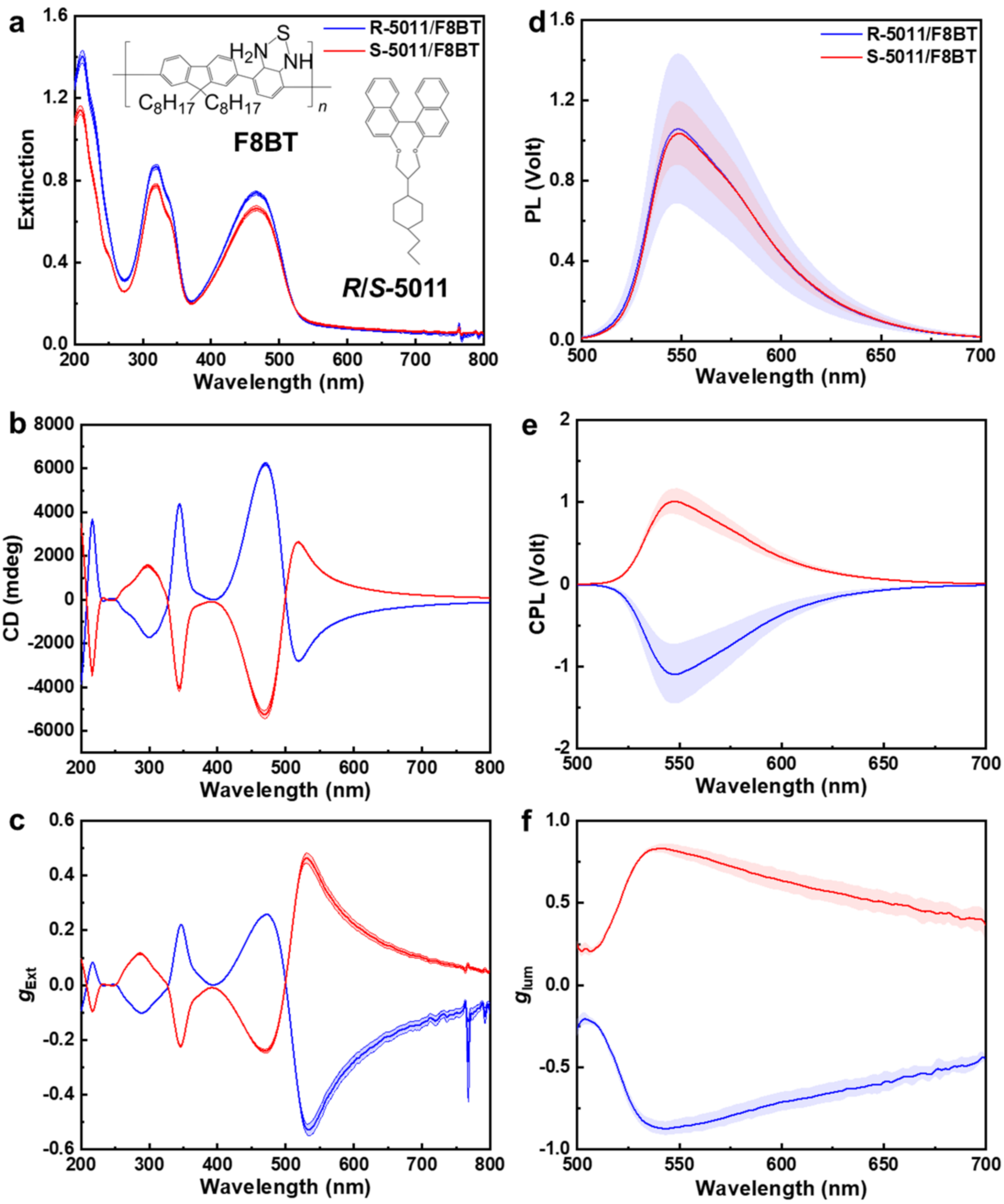

Supplementary Figure 37| Optical characterization of *R/S*-5011/F8BT. UV–visible–NIR spectra: **a** extinction; **b** CD; **c** g_{Ext}. Emission spectra (excited at 330 nm): **d** PL; (e) CPL; **f** g_{lum}. Inserts in (**a**): molecular structures of F8BT and *R/S*-5011. Blue spectra: *R*-5011/F8BT; red spectra: *S*-5011/F8BT. (a–f) Solid lines: mean values; shaded areas: standard deviations; statistically evaluated from multiple measurements. Fabrication conditions: [F8BT] = 21.1 mg mL^{-1}; [*R/S*-5011] = 13.1 mg mL^{-1}; annealing temperature = 198.5 ℃; annealing duration = 27.8 min; spin-coating rate = 2,293 rpm; and spin-coating duration = 59 s.

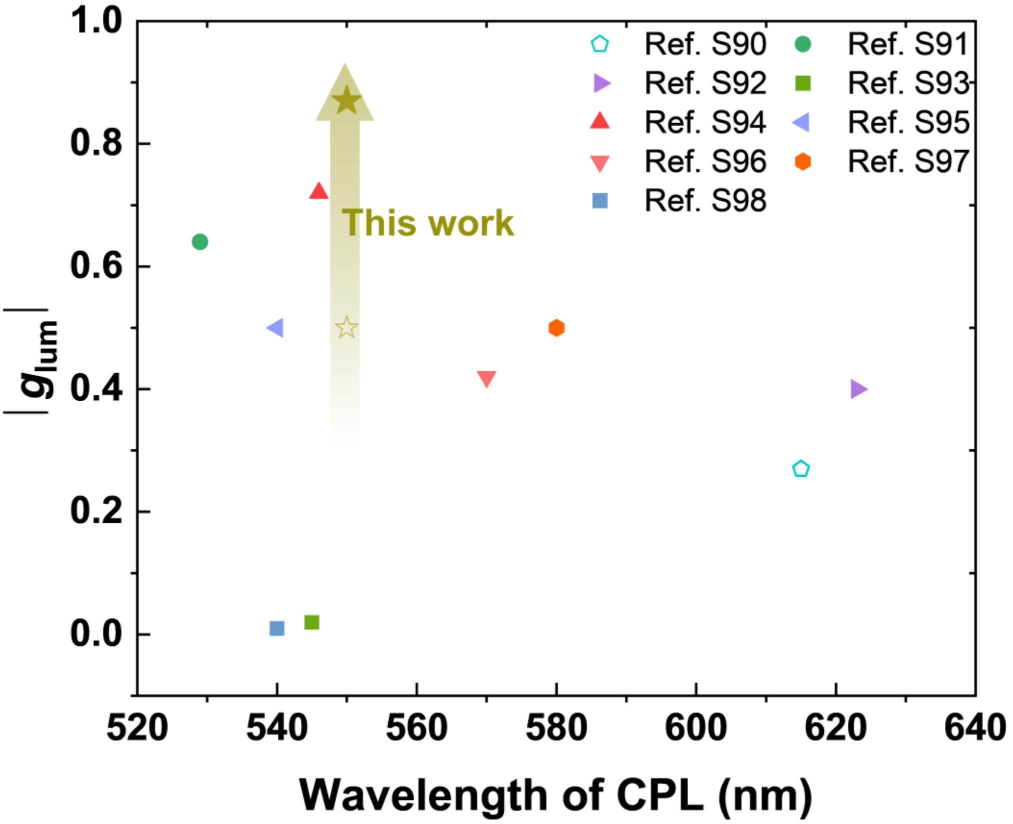

Supplementary Figure 38| Summary of |g_{lum}| of CPL generated from the F8BT-based chiral luminophores (Supplementary Table 2). In this work, |g_{lum}| of *R/S*-5011/F8BT was manually optimized as 0.50 (hollow brown star), which was increased to 0.87 (solid brown star) through the OptiCPL-guided optimization.

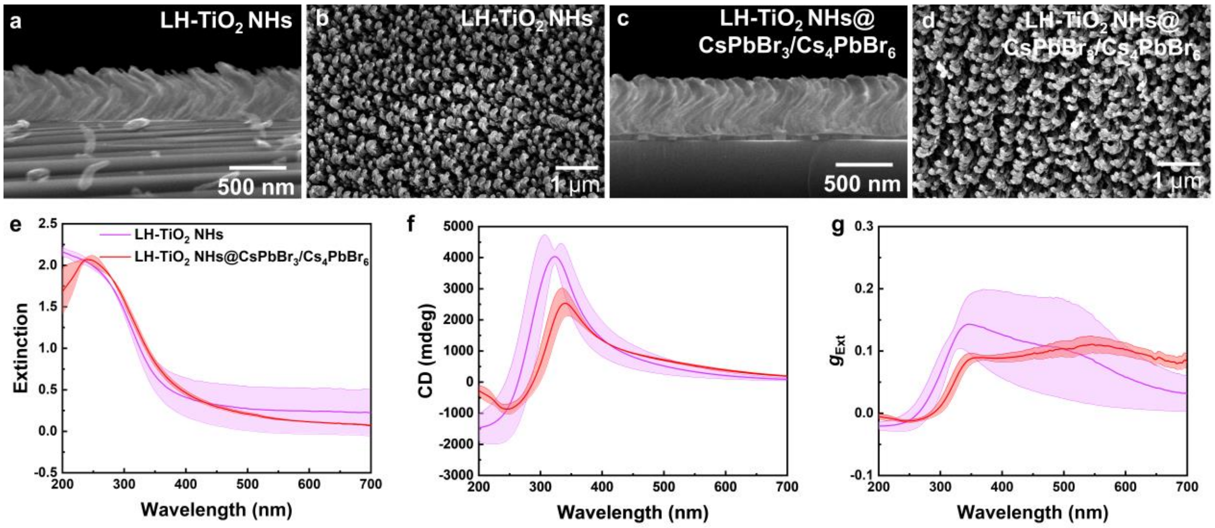

Supplementary Figure 39| Structural and optical characterization of LH-TiO_2 NHs and LH-TiO_2 NHs@$CsPbBr_3$/Cs_4PbBr_6. LH-TiO_2 NHs@$CsPbBr_3$/Cs_4PbBr_6 fabricated under the following experimental conditions that were predicted by OptiCD, to obtain CD = 2000 mdeg for the LH-core@shells: α = 87°, R_d = 0.15 nm s^{-1}, β = 0.33, P = 403.9 nm, n = 0.96, [CsBr] = 9.5 mmol L^{-1}, [$PbBr_2$] = 10.8 mmol L^{-1}, and t_s = 4.7 h. SEM (**a**, **c**) cross-sectional and (**b**, **d**) top-view images: (**a**, **b**) LH-TiO_2 NHs; (**c**, **d**) LH-TiO_2 NHs@$CsPbBr_3$/Cs_4PbBr_6. UV–visible spectra of LH-TiO_2 NHs (pink) and LH-TiO_2 NHs@$CsPbBr_3$/Cs_4PbBr_6 (red): **e** extinction; **f** CD; **g** g_{Ext}. Solid lines: mean values; shaded areas: standard deviations; statistically evaluated from multiple measurements.

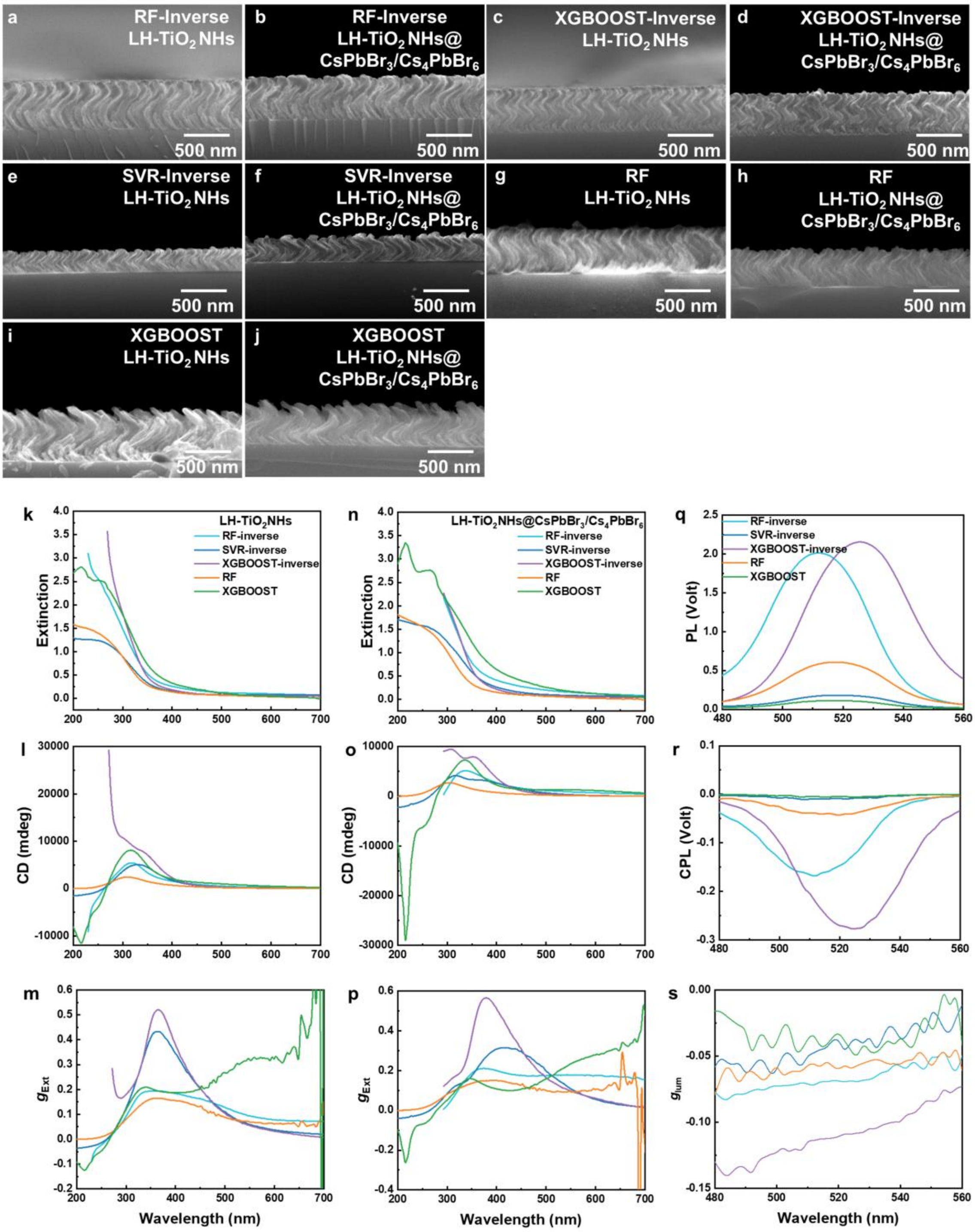

Supplementary Figure 40| Fabrication of LH-TiO_2 NHs and LH-TiO_2 NHs@$CsPbBr_3$/Cs_4PbBr_6 guided with the traditional AI models (including (a, b) RF-inverse, (c, d) XGBOOST-inverse, (e, f) SVR-inverse, (g, h) RF, and (i, j) XGBOOST), with morphological and optical characterizations. a-j SEM cross-sectional images: **a**, **c**, **e**, **g**, **i** LH-cores; **b**, **d**, **f**, **h**, **j** LH-core@shells. UV–visible spectra: **k**, **n** extinction; **l**, **o** CD; **m**, **p** g_{Ext}. Emission spectra excited at 370 nm: **q** PL; **r** CPL; **s** g_{lum}. The Experimental conditions guided by the diverse traditional AI models and those applied to generate the chiral core@shell nano-luminophores are summarized in **Supplementary Table 3 and 4**, respectively.

Supplementary Table 1. Summary of $|g_{lum}|$ generated from diverse kinds of chiral luminophores.

System	$\|g_{lum}\|$	References
Luminophores in chiral templates (liquid crystals serve as chiral templates *, cellulose serve as chiral templates ^)	1.9*	S10
	1.6*	S11
	1.5*	S12
	0.8*	S13
	0.89*	S14
	0.15*	S15
	0.027	S16
	0.0089	S17
	0.004	S18
	0.0031	S19
	0.0091	S20
	0.002	S21
	0.00075	S22
	0.003	S23
	0.0002	S24
	0.44	S25
	0.16	S26
	0.01	S27
	1.1*	S28
	0.4*	S29
	0.2^	S30
	0.38^	S31
	0.45^	S32
	0.8*	S33
	1.5*	S34
	0.5^	S35
	0.76*	S36
	2*	S37
	0.0056^	S38
	1.73*	S39
	0.00514	S40
	0.89^	S41
	1.41*	S42
	0.31*	S43
	0.35	This work

**Inorganic luminophores modified with chiral ligands**	0.068	S44
	0.0082	S45
	0.0023	S46
	0.0073	S47
	0.007	S48
	0.004	S49
	0.0036	S50
	0.0023	S51
	0.006	S52
	0.002	S53
	0.104	S54
	0.352	S55
	0.006	S56
	0.0045	S57
	0.274	S58
	0.06	S59
	0.22	S60
	0.0061	S61
	0.07	S62
	0.026	S63
	0.01	S64
	0.0013	S65
	0.048	S66
**Chiral organic aggregates**	0.32	S67
	0.15	S68
	0.44	S69
	0.004	S70
	0.0145	S71
	0.0045	S72
	0.01	S73
	0.00139	S74
	0.001	S75
	0.125	S76
	0.005	S77
	0.001	S78
	0.00054	S79
	0.349	S80
	0.05014	S81

	0.058	S82
	0.055	S83
	0.0016	S84
	0.87	This work
	0.0095	S85
**Small chiral organic molecules**	0.11	S86
	0.53	S87
	0.024	S88
	0.01	S89

Supplementary Table 2. Summary of CPL performance of the F8BT-based chiral luminophores.

	**Wavelength of CPL (nm)**	**$\lvert g_{lum} \rvert$**	**reference**
***R*/*S*-5011/F8BT**	544	0.87	This work
**F8BT-*R/S*-5011/DBP**	615	0.27	S90
**cholesteric liquid crystals/F8BT(R/S5011)**	529	0.64	S91
**[*M*,*M*]- and [*P*,*P*]-PD8H-6R/F8BT**	623	0.4	S92
**F8BT(*R/S*-6)**	545	0.02	S93
**F8BT (*R/S*-5011)**	546	0.72	S94
**F8BT/ aza[6]helicene**	540	0.5	S95
**F8BT:2 blends**	570	0.42	S96
**F8BT:aza[6]H blends**	580	0.5	S97
**F8BT(*R/S*-3)**	540	0.01	S98

Supplementary Table 3. Experimental parameters predicted by traditional machine learning models.

	α (°)	R_d (nm s^{-1})	β	*P* (nm)	*n*	[CsBr] (mmol L^{-1})	[$PbBr_2$] (mmol L^{-1})	t_s (h)
**RF-inverse**	86.983	0.151	0.330	413.322	1.028	7.162	7.162	4.7169
**XGBOOST-inverse**	86.587	0.174	0.344	260.965	1.833	16.654	16.654	4.138
**SVR-inverse**	86.975	0.149	0.329	285.611	1.155	16.654	16.654	4.840
**RF**	86.399	0.125	0.385	350.687	1.030	8.037	8.037	3.790
**XGBOOST**	86.757	0.184	0.362	549.900	1.000	4.000	4.000	5.629
**SVR**	85.746	0.100	0.330	600.000	1.000	4.870	4.870	5.640

Supplementary Table 4. Experimental parameters applied to fabricate chiral core@shell nano-luminophores, guided by traditional machine learningmodels.

	α (°)	R_d (nm/s)	β	P (nm)	n	[CsBr] (mmol L^{-1})	[$PbBr_2$] (mmol L^{-1})	t_s (h)
**RF-inverse**	87.0	0.15	0.33	413	1.02	7.2	7.2	4.7
**XGBOOST-inverse**	86.5	0.17	0.34	261	1.83	16.7	16.7	4.1
**SVR-inverse**	87.0	0.15	0.33	286	1.16	8.0	8.0	4.8
**RF**	86.5	0.13	0.39	351	1.03	4.0	4.9	3.8
**XGBOOST**	86.5	0.18	0.36	550	1	4.0	4.0	5.6
**SVR***	~~86.0~~	~~0.1~~	~~0.33~~	~~600~~	~~1~~	~~4.87~~	~~4.87~~	~~5.6~~

*The SVR's predicted structure was calculated to have a rotational speed of 0.0198 °/s for its R_r (according to equation 4 in manuscript). This parameter is far below the instrument's designed minimum rotational speed (0.04 °/s), therefore it could not be achieved.

Supplementary references

1. Rousseeuw, P. A graphical aid to the interpretation and validation of cluster analysis. *J. Comput. Appl. Math.* **20**, 53-65 (1987)

2. Dl, D. & Bouldin, D. A cluster separation measure. *IEEE Trans. Pattern Anal. Mach. Intell.* **1**, 224-227 (1979)

3. Drucker, H., Burges, C., Kaufman, L., Smola, A. & Vapnik, V. Support vector regression machines. *Advances in neural information processing systems* **9** (1996)

4. Breiman, L. Random forests. *Mach. Learn.* **45**, 5-32 (2001)

5. Friedman, J. Greedy function approximation: A gradient boosting machine. *Ann. Stat.* **29**, 1189-1232 (2001)

6. Freund, Y. & Schapire, R. A decision-theoretic generalization of on-line learning and an application to boosting. *J. Comput. Syst. Sci. Int.* **55**, 119-139 (1997)

7. Cover, T. & Hart, P. Nearest neighbor pattern classification. *IEEE Trans. Inf. Theory* **13**, 21-27 (1967)

8. Hoerl, A. & Kennard, R. Ridge regression: Biased estimation for nonorthogonal problems. *Technometrics* **12**, 55-67 (1970)

9. Zou, H. & Hastie, T Regularization and variable selection via the elastic net. *J. R. Stat. Soc. Ser. B. Stat. Methodol* **67**, 301-320 (2005)

10. Liu, S., et al. Circularly polarized perovskite luminescence with dissymmetry factor up to 1.9 by soft helix bilayer device. *Matter* **5**, 2319-2333 (2022)

11. Wang, C., et al. Fully chiral light emission from cspbx3 perovskite nanocrystals enabled by cholesteric superstructure stacks. *Adv. Funct. Mater.* **29**, 1903155 (2019)

12. Zhang, X., et al. Mechanically tunable circularly polarized luminescence of liquid crystal-templated chiral perovskite quantum dots. *Angew. Chem. Int. Ed.* **22**, e202404202 (2024)

13. Zhou, Y., et al. Helical-caging enables single-emitted large asymmetric full-color circularly polarized luminescence. *Nat. Commun.* **15**, 251 (2024)

14. Guo, Q., et al. Multimodal-responsive circularly polarized luminescence security materials. *J. Am. Chem. Soc.* **145**, 4246-4253 (2023)

15. Zhang, P., et al. Ultrastable dual-matrix meditated cspbbr3 composites with enhanced photoluminescence quantum yield and robust circular polarization luminescence. *Chem. Eng. J.* **480**, 148306 (2024)

16. Liu, Y., Yang, K., Wu, Y. & Deng, J. “Surface-filming assembly” strategy for facilely constructing and adjusting circularly polarized luminescence from perovskite/chiral helical polyacetylene films. *Adv. Opt. Mater.* **12**, 2303039 (2024)

17. Liang, Z., et al. Continuous production of stable chiral perovskite nanocrystals in electrospinning nanofibers to exhibit circularly polarized luminescence. *J. Mater. Chem. C* **10**, 12644-12651 (2022)

18. Ye, C., Jiang, J., Zou, S., Mi, W. & Xiao, Y. Core-shell three-dimensional

perovskite nanocrystals with chiral-induced spin selectivity for room-temperature spin light-emitting diodes. *J. Am. Chem. Soc.* **144**, 9707-9714 (2022)

19. Ru, Y., et al. Full-color circularly polarized luminescence of cspbx(3) nanocrystals triggered by chiral carbon dots. *Adv. Mater.* **35**, e2207265 (2023)

20. Wang, Q., et al. Strong circularly polarized luminescence from quantum dots/2d chiral perovskites composites. *Nano Res.* **16**, 7593-7599 (2023)

21. Liu, P., et al. Chiral perovskite nanocrystal growth inside helical hollow silica nanoribbons. *Nano Lett.* **23**, 3174-3180 (2023)

22. Zhang, C., Li, Z., Dong, X., Niu, Y. & Zang, S. Multiple responsive CPL switches in an enantiomeric pair of perovskite confined in lanthanide MOFs. *Adv. Mater.* **34**, e2109496 (2022)

23. Nuzzo, D, et al. Circularly polarized photoluminescence from chiral perovskite thin films at room temperature. *ACS Nano* **14**, 7610-7616 (2020)

24. Kim, M., et al. Simultaneously achieving room-temperature circularly polarized luminescence and high stability in chiral perovskite nanocrystals via block copolymer micellar nanoreactors. *J. Mater. Chem. A* **11**, 12876-12884 (2023)

25. Su, Y., et al. Magnetic assembly of magnetite/perovskite hybrid nanorods for circularly polarized luminescence. *Adv. Funct. Mater.* **34**, 2403629 (2024)

26. Mendoza-Carreno, J., et al. Nanoimprinted 2d-chiral perovskite nanocrystal metasurfaces for circularly polarized photoluminescence. *Adv. Mater.* **35**, e2210477 (2023)

27. Fu, K. & Liu, G. Full-color circularly polarized luminescence of supramolecular polymers with handedness inversion regulated by anion and temperature. *ACS Nano* **18**, 2279-2289 (2024)

28. Yang, X., Zhou, M., Wang, Y. & Duan, P. Electric-field-regulated energy transfer in chiral liquid crystals for enhancing upconverted circularly polarized luminescence through steering the photonic bandgap. *Adv. Mater.* **32**, e2000820 (2020)

29. Choi, Y., et al. Circularly polarized light emission from nonchiral perovskites incorporated into nanoporous cholesteric polymer templates. *ACS Nano* **18**, 909-918 (2024)

30. Xiong, R., et al. Self-assembly of emissive nanocellulose/quantum dot nanostructures for chiral fluorescent materials. *ACS Nano* **13**, 9074-9081 (2019)

31. Zheng, H., et al. Circularly polarized luminescent carbon dot nanomaterials of helical superstructures for circularly polarized light detection. *Adv. Opt. Mater.* **6**, 1801246 (2018)

32. Shi, Y., et al. Circularly polarized luminescence from semiconductor quantum rods templated by self-assembled cellulose nanocrystals. *J. Mater. Chem. C* **8**, 1048-1053 (2020)

33. Zhang, M., et al. Processable circularly polarized luminescence material

enables flexible stereoscopic 3d imaging. *Sci. Adv.* **9**, eadi9944 (2023)

34. Zhang, X., et al. Mechanically tunable circularly polarized luminescence of liquid crystal-templated chiral perovskite quantum dots. *Angew. Chem. Int. Ed.* **63**, e202404202 (2024)

35. Jia, S., et al. Dual-direction circularly polarized luminescence materials with on-demand handedness and superior flexibility. *Adv. Funct. Mater.* **34**, 2410206 (2024)

36. Lin, S., Ren, T., Meng, X., Kang, W. & Guo, J. Light-triggered fluorochromic cholesteric liquid crystal elastomer with hydrogen-bonded fluorescent switch: Dualmodal-switchable circularly polarized luminescence. *Sci. China Chem.* **67**, 2719-2727 (2024)

37. Ji, M. Zhao, W., Li, M. & Chen, C. Circularly polarized luminescence with high dissymmetry factors for achiral organic molecules in solutions. *Nat. Commun.* **16**, 2940 (2025)

38. Sun, Y., et al. Microbe-assisted fabrication of circularly polarized luminescent bacterial cellulosic hybrids. *Nat. Commun.* **16**, 1115 (2025)

39. Xu, L., et al. Circularly polarized luminescence with large dissymmetry factors based on perovskite and cholesteric liquid crystal polymer network films. *J. Mater. Chem. C* **13**, 7544-7549 (2025)

40. Zhang, K., et al. Multi-stimuli-responsive circularly polarized luminescence with handedness inversion and near-infrared phosphorescence in chiral metal-organic framework platform for white light emission and information encryption. *Adv. Sci.* **12**, e2502784 (2025)

41. Yuan, B., et al. Tunable and responsive circularly polarized luminescence of self-organized cellulose nanocrystal chiral superstructures loaded with aie luminogen. *Adv. Funct. Mater.* **35**, 2424601 (2025)

42. Yang, N., Zhao, J, Liu, W., Li, Y. & Yang, Y. Cholesteric liquid crystal polymer networks doped with an axially chiral fluorescent dye: Circularly polarized luminescence and anti-counterfeiting applications. *Adv. Opt. Mater.* **13**, 2402128 (2024)

43. Chen, Y., et al. Enhanced circularly polarized luminescence modulation using dichroic conjugated polymer in cholesteric liquid crystals. *Adv. Opt. Mater.* **13**, 2403100 (2024)

44. Kim, Y., et al. Strategies to achieve high circularly polarized luminescence from colloidal organic-inorganic hybrid perovskite nanocrystals. *ACS Nano* **14**, 8816-8825 (2020)

45. Cao, R., Yang, X., Wang, Y. & Xiao, Y. Induced circularly polarized luminescence of perovskite nanocrystals by self-assembly chiral gel. *Nano Res.* **16**, 1459-1464 (2022)

46. Chai, C., et al. Single-component white circularly polarized luminescence in chiral 1d double-chain perovskites. *Adv. Opt. Mater.* **11**, 2201996 (2022)

47. Shi, Y., et al. Endowing perovskite nanocrystals with circularly polarized

luminescence. *Adv. Mater.* **30**, e1705011 (2018)
48. Chen, W., et al. Two-photon absorption-based upconverted circularly polarized luminescence generated in chiral perovskite nanocrystals. *J. Phys. Chem. Lett.* **10**, 3290-3295 (2019)
49. Tao, L., Zhan, H. Cheng, Y., Qin, C. & Wang, L. Enhanced circularly polarized photoluminescence of chiral perovskite films by surface passivation with chiral amines. *J. Phys. Chem. Lett.* **14**, 2317-2322 (2023)
50. Jia, T., et al. Enantiomeric alkynyl-protected au10 clusters with chirality-dependent radiotherapy enhancing effects. *Nano Today* **39**, 101222 (2021)
51. Chen, S., et al. Chiral multidentate ligand facilitating perovskite nanocrystals with circularly polarized luminescence and chiral assembly. *Adv. Opt. Mater.* **12**, 2302883 (2024)
52. Lin, J., et al. Tuning the circular dichroism and circular polarized luminescence intensities of chiral 2d hybrid organic-inorganic perovskites through halogenation of the organic ions. *Angew. Chem. Int. Ed.* **60**, 21434-21440 (2021)
53. Zhao, X., et al. An enantiomeric pair of 2d organic–inorganic hybrid perovskites with circularly polarized luminescence and photoelectric effects. *J. Mater. Chem. C* **10**, 3440-3446 (2022)
54. He, C., et al. Room temperature circularly polarized emission in perovskite nanocrystals through bichiral-molecule-induced lattice reconstruction. *Matter* **7**, 475-484 (2024)
55. Ma, J., et al. Chiral 2d perovskites with a high degree of circularly polarized photoluminescence. *ACS Nano* **13**, 3659-3665 (2019)
56. Liu, P., et al. Optically active perovskite $cspbbr_3$ nanocrystals helically arranged on inorganic silica nanohelices. *Nano Lett.* **20**, 8453-8460 (2020)
57. Jin, X., et al. A new strategy to achieve enhanced upconverted circularly polarized luminescence in chiral perovskite nanocrystals. *Nano Res.* **15**, 1047-1053 (2021)
58. Wang, J., et al. Aqueous synthesis of low-dimensional lead halide perovskites for room-temperature circularly polarized light emission and detection. *ACS Nano* **13**, 9473-9481 (2019)
59. Long, G., et al. Spin control in reduced-dimensional chiral perovskites. *Nat. Photonics* **12**, 528-533 (2018)
60. Qin, Y., et al. Multifunctional chiral 2d lead halide perovskites with circularly polarized photoluminescence and piezoelectric energy harvesting properties. *ACS Nano* **16**, 3221-3230 (2022)
61. Yao, Q., et al. Generation and enhancement of chirality-induced spin-polarized emission in 3d@1d lead halide perovskites. *ACS Mater. Lett.* **6**, 2186-2196 (2024)
62. Yao, J., et al. Efficient green spin light-emitting diodes enabled by ultrafast

energy- and spin-funneling in chiral perovskites. *J. Am. Chem. Soc.* **146**, 14157-14165 (2024)

63. Li, J., et al. Rashba effect and spin-dependent excitonic properties in chiral two-dimensional/three-dimensional composite perovskite films. *J. Phys. Chem. Lett.* **14**, 11697-11703 (2023)

64. Yang, L. S., et al. Circularly polarized photoluminescence of chiral 2d halide perovskites at room temperature. *ACS Appl. Mater. Interfaces* **14**, 54090-54100 (2022)

65. Patra, S., Dhiman, S. & George, S. Precision synthesis of circularly polarized luminescence active organic assemblies in aqueous media via efficient living supramolecular polymerization. *Chem. Mater.* **36**, 9460-9468 (2024)

66. Zhai, L., et al. Efficient circularly polarized luminescence from mn-br hybrid perovskite assembled by achiral architectures. *Angew. Chem. Int. Ed.* **64**, e202425543 (2025)

67. Liu, J., et al. What makes efficient circularly polarised luminescence in the condensed phase: Aggregation-induced circular dichroism and light emission. *Chem. Sci.* **3**, 2737-2747 (2012)

68. Wade, J., et al. 500-fold amplification of small molecule circularly polarised luminescence through circularly polarised fret. *Angew. Chem. Int. Ed.* **60**, 222-227 (2021)

69. Li, S., et al. Advances in circularly polarized luminescence materials based on helical polymers. *J. Mater. Chem. C* **11**, 1242-1250 (2023)

70. Feng, H., Gu, X., Lam, J., Zheng, Y. & Tang, B. Design of multi-functional aiegens: Tunable emission, circularly polarized luminescence and self-assembly by dark through-bond energy transfer. *J. Mater. Chem. C* **6**, 8934-8940 (2018)

71. Ma, J., et al. Aggregation-induced cpl response from chiral binaphthyl-based aie-active polymers via supramolecular self-assembled helical nanowires. *Polymer* **143**, 184-189 (2018)

72. Liu, Q., Xia, Q., Wang. S., Li, B. & Tang, B. In situ visualizable self-assembly, aggregation-induced emission and circularly polarized luminescence of tetraphenylethene and alanine-based chiral polytriazole. *J. Mater. Chem. C* **6**, 4807-4816 (2018)

73. Zhao, N., et al. Regulation of circular dichroism behavior and construction of tunable solid-state circularly polarized luminescence based on binol derivatives. *Mater. Chem. Front.* **3**, 1613-1618 (2019)

74. Hu, Y., et al. Circularly polarized luminescence from chiral conjugated poly(carbazole-ran-acridine)s with aggregation-induced emission and delayed fluorescence. *ACS Appl. Polym. Mater.* **1**, 221-229 (2019)

75. Zhao, Z., et al. Non-aromatic annulene-based aggregation-induced emission system via aromaticity reversal process. *Nat. Commun.* **10**, 2952 (2019)

76. Xue, S., et al. Unexpected aggregation induced circular dichroism, circular polarized luminescence and helical assembly from achiral hexaphenylsilole (HPS). *RSC Adv.* **7**, 24841-24847 (2017)

77. Langeveld-Voss, B., et al. Circular dichroism and circular polarization of photoluminescence of highly ordered poly{3,4-di[(s)-2-methylbutoxy]thiophene}. *J. Am. Chem. Soc.* **118**, 4908-4909 (1996)

78. Li, F., et al. CPL emission of chiral binol-based polymers via chiral transfer of the conjugated chain backbone structure. *RSC Adv.* **5**, 105851-105854 (2015)

79. Meng, F., et al. Reversal aggregation-induced circular dichroism from axial chirality transfer via self-assembled helical nanowires. *RSC Adv.* **7**, 15851-15856 (2017)

80. Jiang, X., et al. Fluorescence study of chiral β-ketoiminate-based newly synthesized boron hybrid polymers. *Macromol. Chem. Phys.* **215**, 358-364 (2014)

81. Wu, S., Song, X. Du, C. & Liu, M. Macroscopic homochiral helicoids self-assembled via screw dislocations. *Nat. Commun.* **15**, 6233 (2024)

82. He, J., et al. [2.2]paracyclophane-based double helices: Tunable circularly polarized luminescence driven by self-assembly. *Adv. Opt. Mater.* **12**, 2302221 (2023)

83. He, M., et al. Reversible circularly polarized luminescence inversion and tunable emission in chloride-bridge-linked supramolecular polymers. *Adv. Funct. Mater.* e10772 (2025)

84. Cai, X., et al. Circularly polarized luminescence of single-handed helical tetraphenylethylene-silica nanotubes. *Chem. Commun.* **55**, 12176-12179 (2019)

85. Wang, J., et al. Chiral phosphine-copper iodide hybrid cluster assemblies for circularly polarized luminescence. *J. Am. Chem. Soc.* **143**, 10860-10864 (2021)

86. Liu, Z., et al. Intense circularly polarized luminescence induced by chiral supramolecular assembly: The importance of intermolecular electronic coupling. *Angew. Chem. Int. Ed.* **63**, e202407135 (2024)

87. Zhao, T., et al. Amino-acid-induced circularly polarized luminescence of octahedral lanthanide cage. *Angew. Chem. Int. Ed.* **64**, e202421426 (2025)

88. Yu, Y., et al. Pi-extended heli(aminoborane)s with highly bright circularly polarized luminescence and narrowband emission. *Angew. Chem. Int. Ed.* **64**, e202501645 (2025)

89. Zhao, J., et al. Boosted circularly polarized luminescence of a chiral metallacage through co-assembly in confined microfluidic environments. *Adv. Funct. Mater.* **35**, 2413920 (2024)

90. Sun, H., Yuan, C., Nishmura, T., Ikai, T. & Murata, H. Achieving high circularly polarized red luminescence and photoluminescence quantum yield through chiral induction and energy transfer in F8BT-based systems. *Jpn. J. Appl. Phys.* **64**, 10SP30 (2025)

91. Li, Y., et al. Enhanced circularly polarized luminescence emission promoted by achiral dichroic oligomers of f8bt in cholesteric liquid crystal. *Chin. Chem. Lett.* **35**, 109864 (2024)
92. Wan, L., et al. Giant circularly polarized luminescence driven by excited-state hybridization between molecular emitters and chiral environments. *Adv. Mater.* **37**, 2506941 (2025)
93. Zhang, X., Xu, Z., Zhang, Y. Quan, Y. & Cheng, Y. Controllable circularly polarized electroluminescence performance improved by the dihedral angle of chiral-bridged binaphthyl-type dopant inducers. *ACS Appl. Mater. Interfaces* **13**, 55420-55427 (2021)
94. Lee,D., Song, J. Lee, Y. Yu, C. & Kim, J. Control of circularly polarized electroluminescence in induced twist structure of conjugate polymer. *Adv. Mater.* **29**, 1700907 (2017)
95. Yang, Y., Costa, R., Smilgies, D., Campbell, A. & Fuchter, M. Induction of circularly polarized electroluminescence from an achiral light-emitting polymer via a chiral small-molecule dopant. *Adv. Mater.* **25**, 2624 (2013)
96. Morgenroth, M., Scholz, M., Guy, L., Oum, K. & Lenzer, T. Spatiotemporal mapping of efficient chiral induction by helicene-type additives in copolymer thin films. *Angew. Chem. Int. Ed.* **61**, e202203075 (2022)
97. Yan, H., et al. Enhancing hole carrier injection via low electrochemical doping on circularly polarized polymer light-emitting diodes. *J. Mater. Chem. C* **10**, 9512-9520 (2022)
98. Zhang, X., Xu, Z., Zhang, Y., Quan, Y. & Cheng, Y. High brightness circularly polarized electroluminescence from conjugated polymer f8bt induced by chiral binaphthyl-pyrene. *J. Mater. Chem. C* **8**, 15669-15676 (2020)
