# Supplementary material for: Multimodal deep-learning optimization of chiroptical properties in all-inorganic perovskite-coated TiO2 nanohelices and inverse-design transfer to organic chiral luminophores
Source: Nat Commun. 2026 Jun 4;17:7180. doi: 10.1038/s41467-026-74010-2 (PMC13396460; doi:10.1038/s41467-026-74010-2)
Supplement: Supplementary file 3 — Transparent Peer Review file [file 41467_2026_74010_MOESM3_ESM.pdf]

nature portfolio

Peer Review File

Multimodal deep-learning optimization of chiroptical properties in all-inorganic perovskite-coated TiO2 nanohelices and inverse-design transfer to organic chiral luminophores

Corresponding Author: Professor Zhifeng Huang

This file contains all reviewer reports in order by version, followed by all author rebuttals in order by version.

Version 0:

Reviewer comments:

Reviewer #1

(Remarks to the Author)
H. Sun et al reported materials informatics for facile design of CPL-indicating composite materials through multimodal deep learning.

(1) What are the noteworthy results?
Precise design of TiO2 nanofelices@CsPbBr3/Cs4PbBr6 core-shell composites and optimization of the deposition angle, deposition rate, helical pitch, the number of the pitches, concentration of the starting materials of [CsBr] and [PbBr2], and soaking period for preparation conditions in terms of the higher luminescence dissymmetry factor through the systematic experimental comparisons are the noteworthy results.

(2) Will the work be of significance to the field and related fields?
As the authors mentioned by showing Figure 1, finding the CPL-generating materials have been attracted by many researchers. In this context, this work is supportive to accelerate the field of the materials designers using the method of multimodal deep learning.

(3) Does the work support the conclusions and claims, or is additional evidence needed?
Following points are still required further explanation:
i) In line 1 of page 9, why 87˚ is the best angle? The authors merely quoted ref 63. Further elucidation should be described in the manuscript.
ii) In line 1 of page 12, the suppression mechanisms of the chiroptical activities of the thicker shell of the CsPbBr3/Cs4PbBr6 surrounding the TiO2 core are still uncertain even though the authors referred refs 68-70. The authors should describe the details of the reasons why the change of dielectric permittivity of the CsPbBr3/Cs4PbBr6 resulted in the weakening their chiroptical activities.
iii) In line 9 of page 12, what is the gear backlash?

(4) Are there any flaws in the data analysis, interpretation and conclusions?
In line 17 of page 13, the word of “coin” is correct? Is it coil?

(5) Is the methodology sound?
Yes

(6) Is there enough detail provided in the methods for the work to be reproduced?
Yes

(7) Additional comments:
The authors should recheck the list of references. For examples, refs 31 and 33 are the same. Refs 25 and 71 are also the same.

(Remarks on code availability)

Reviewer #2

(Remarks to the Author)
This manuscript reports an artificial intelligence–assisted strategy, termed OptiCPL, for optimizing circularly polarized luminescence (CPL) in chiral core–shell nanostructures based on GLAD-fabricated TiO_2 nanohelices with tunable pitches. These morphochiral helices act as templates that transfer their handedness to conformally coated perovskite shells ($CsPbBr_3$/Cs_4PbBr_6), producing green CPL emitters with manually optimized g_lum values up to 0.20.

To overcome the limitations of conventional trial-and-error optimization, the authors introduce a reverse-design framework that integrates multimodal data—including SEM-derived morphological features and spectroscopic responses. OptiCPL predicts synthesis conditions that maximize the luminescence dissymmetry factor. Guided by this approach, |g_lum| is enhanced by 75% to 0.35 in inorganic systems, and further boosted from 0.50 to 0.87 in polymer emitters, demonstrating strong generality and transferability.

The manuscript reports several noteworthy advances:
(a) record enhancement of CPL signals using an AI-guided strategy;
(b) identification of pitch-dependent resonant behavior governing CPL amplification;
(c) simultaneous learning from SEM images and spectroscopic data within a unified framework; and
(d) a conceptual shift from trial-and-error optimization to property-driven inverse design.

The work addresses a major bottleneck in CPL materials research—navigating complex synthetic parameter spaces to optimize chiroptical performance. By linking target CPL spectra directly to synthesis parameters, the study establishes a powerful materials informatics approach aligned with the physics of chiral photonic nanostructures, making a significant contribution at the intersection of chiral photonics and AI-assisted materials design.

Despite these strengths, several aspects could be clarified or strengthened. First, the framework is formulated specifically for CPL optimization. Although CD spectra are included as auxiliary inputs, the manuscript does not discuss how OptiCPL could be adapted to directly design or optimize CD responses, which would broaden its impact in chiroptical materials research. Second, while the model successfully captures pitch-dependent resonant behavior, it is not clear which specific electromagnetic quantities the model is using. A clearer connection between learned variables and underlying electromagnetic modes or structural descriptors would improve physical interpretability. In addition, uncertainties associated with SEM-based morphology—such as resolution limits, segmentation accuracy, and nanoscale variations in helix width or shell coverage—are not discussed in relation to model robustness. Finally, the framework is validated only for dielectric helical emitters where CPL arises from photonic mode coupling. Its applicability to systems governed by different mechanisms, such as molecular CPL emitters, remains unexplored and should be clarified.

To summarize, the AI-guided optimization demonstrates substantial improvements in g_lum for both inorganic and organic systems, underscoring the effectiveness and generality of the approach. The study therefore represents a significant step forward in data-driven design of chiroptical materials.

(Remarks on code availability)

Reviewer #3

(Remarks to the Author)
This manuscript reports an all-inorganic chiral core@shell nano-luminophore system optimized using a multimodal deep learning framework (OptiCPL) to enhance circularly polarized luminescence. The experimental platform is carefully constructed, and the achieved glum values are competitive for all-inorganic CPL materials, with the extension to an organic polymer system representing broader applicability. However, several major issues remain regarding the practical effectiveness of the machine-learning-assisted optimization and the overall rigor of the manuscript presentation. In particular, some claims appear insufficiently supported by the available data, and minor but non-negligible issues in data interpretation and reference handling detract from the clarity and reliability of the study.
1. The authors state that machine learning is employed to reduce experimental effort and accelerate optimization. However, the OptiCPL model requires extensive inputs, including synthesis parameters, detailed morphological information, and multiple spectral datasets, all of which demand a substantial number of experiments and characterizations. It is therefore unclear whether this approach meaningfully reduces the overall experimental burden compared to careful human-guided optimization. The authors should clarify the practical advantage of OptiCPL in terms of experimental cost and efficiency.
2. The OptiCPL-predicted optimal parameters closely resemble the conditions associated with peak glum values already identified in Figure 3 through manual analysis. This raises the question of whether similar optimization outcomes could be achieved by directly interpreting the experimental trends without a deep learning model. While an optimization framework that operates effectively with truly limited data would be highly valuable, the dataset used here is relatively large for an

experimental study. The authors should therefore better justify the added value of OptiCPL over conventional data-driven optimization performed by an experienced experimentalist using the same dataset.
3. The application of OptiCPL to an organic polymer system (F8BT/R,S-5011) is a positive step toward demonstrating generality. However, molecular weight is a key parameter in polymer-based chiroptical systems and is not included as an experimental variable in this study. The authors should clarify whether molecular weight effects were considered and discuss the limitations this omission imposes on the claimed generality of the framework.
4. References 25 and 71 correspond to the same publication: Liu, P., et al., Optically active perovskite $CsPbBr_3$ nanocrystals helically arranged on inorganic silica nanohelices, Nano Letters 20, 8453–8460 (2020).

(Remarks on code availability)

Version 1:

Reviewer comments:

Reviewer #1

(Remarks to the Author)
The authors well-addressed all the issues listed in the reviewers' reports, thus allowing to improve the quality of the work. I read this new version very carefully, in particular all new parts, and confirmed that the present revised form now can be ready for publication in the Nature Communications.

(Remarks on code availability)

Reviewer #2

(Remarks to the Author)
The reviewer acknowledges the authors' strong efforts in addressing the points raised, which covered mainly four aspects: (i) extending the framework beyond CPL to directly design CD responses; (ii) clarifying the physical interpretability of the aquired results and their link to electromagnetic descriptors; (iii) discussing uncertainties associated with SEM-based morphology and the robustness of the model; and (iv) clarifying the applicability of OptiCPL to CPL systems governed by different mechanisms. The authors provided sound clarifications through important additions, including a new CD-targeted variant (OptiCD) with experimental validation (Supplementary Fig. 39), a statistically significant correlation between the gExt,NHs proxy and |glum| (Supplementary Fig. 26), Grad-CAM attribution maps demonstrating physically meaningful attention on helical pitch-textured regions (Supplementary Fig. 30), and a quantitative noise-perturbation robustness test confirming the stability of the morphology modality (Supplementary Fig. 31). The authors have also added a dedicated "Application scope of OptiCPL" section.
As a conclusion, the reviewer supports the publication of the manuscript in its revised format.

(Remarks on code availability)

Reviewer #3

(Remarks to the Author)
I appreciate the authors' additional explanation regarding the practical motivation for introducing OptiCPL. However, from the manuscript alone, it is still not entirely clear whether the machine learning framework is explicitly addressing the coupled effects among multiple experimental parameters, rather than primarily learning the trends of individually varied parameters. As currently presented, the work may still be read as a well-executed parameter-trend optimization study, and this makes the conceptual distinction from conventional experimental optimization less evident than it could be.
I therefore encourage the authors to revise the manuscript to more clearly highlight how multi-parameter coupling is incorporated into the ML framework and why this aspect provides an advantage beyond simple trend analysis. Making this point more explicit would help readers better understand the uniqueness and significance of the work. If this issue is clearly addressed in the revised manuscript, I would be happy to support acceptance.

(Remarks on code availability)

Open Access This Peer Review File is licensed under a Creative Commons Attribution 4.0 International License, which permits use, sharing, adaptation, distribution and reproduction in any medium or format, as long as you give appropriate credit to the original author(s) and the source, provide a link to the Creative Commons license, and indicate if changes were made.
In cases where reviewers are anonymous, credit should be given to 'Anonymous Referee' and the source.
The images or other third party material in this Peer Review File are included in the article's Creative Commons license, unless indicated otherwise in a credit line to the material. If material is not included in the article's Creative Commons license and your intended use is not permitted by statutory regulation or exceeds the permitted use, you will need to obtain permission directly from the copyright holder.
To view a copy of this license, visit https://creativecommons.org/licenses/by/4.0/

RESPONSE TO THE REVIEWERS' COMMENTS

Reviewer #1

H. Sun et al reported materials informatics for facile design of CPL-indicating composite materials through multimodal deep learning.

(1) What are the noteworthy results?

Precise design of TiO2 nanofelices@CsPbBr3/Cs4PbBr6 core-shell composites and optimization of the deposition angle, deposition rate, helical pitch, the number of the pitches, concentration of the starting materials of [CsBr] and [PbBr2], and soaking period for preparation conditions in terms of the higher luminescence dissymmetry factor through the systematic experimental comparisons are the noteworthy results

(2) Will the work be of significance to the field and related fields? As the authors mentioned by showing Figure 1, finding the CPL-generating materials have been attracted by many researchers. In this context, this work is supportive to accelerate the field of the materials designers using the method of multimodal deep learning.

Response: We sincerely appreciate the reviewer's positive feedback on our manuscript and constructive comments. Accordingly, we have responded to all the comments point-to-point and made revision in the manuscript, as follows.

(3) Does the work support the conclusions and claims, or is additional evidence needed? Following points are still required further explanation:

i) In line 1 of page 9, why 87˚ is the best angle? The authors merely quoted ref 63. Further elucidation should be described in the manuscript.

Response: We are thankful for this insightful comment. In the previously

submitted manuscript (**Line 11–16, Pg. 15**), we had discussed the optimization of the GLAD deposition angle (α), as follows: "First of all, GLAD was performed at α varying in a range of 86.5°–89°, and the fabrication of TiO_2 NHs was optimized at α = 87° (**Supplementary Fig. 12**). The NHs could hardly be formed at α > 88.5°.[74] α < 87° made the NH arrays too close-packed to facilitate the conformal shell-coating, while the loose-packed NH arrays deposited at α > 87° exhibited weak PL and CPL emission."

To support the claim that "α < 87° made the NH arrays too close-packed to facilitate the conformal shell-coating, while the loose-packed NH arrays deposited at α > 87° exhibited weak PL and CPL emission", **Supplementary Figs. 12m-o** were added, as shown below. The close-packed NH arrays could not be reliably obtained at deposition angles of 89°, 88.5°, and 88°. As shown in **Supplementary Fig. 12o-III**, g_{lum} was maximized at α = 87°, validating our claim that "the fabrication of TiO_2 NHs was optimized at α = 87°".

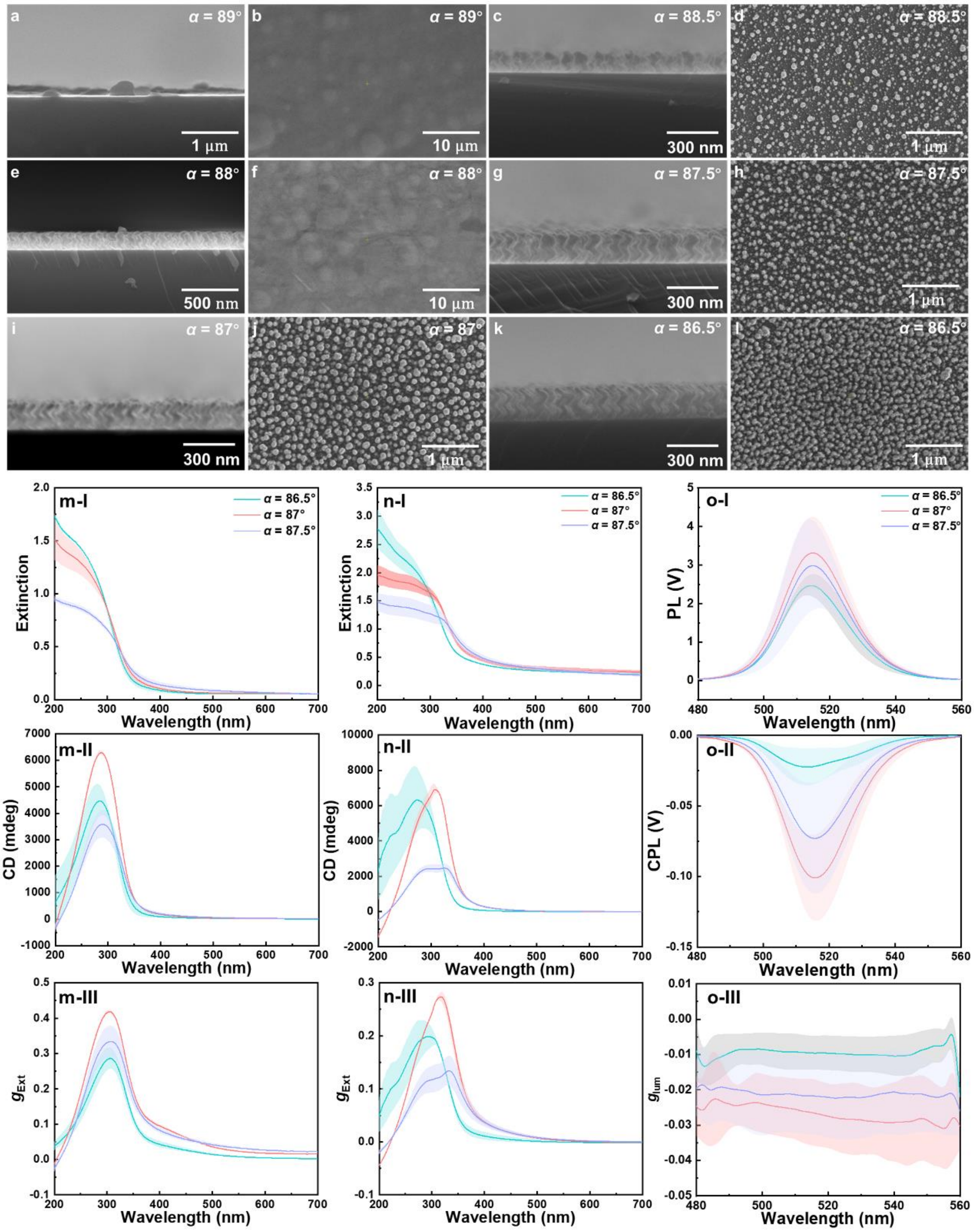

Supplementary Figure 12| Structural and optical characterization of LH-TiO_2 NHs (*P* = 100 nm, and *n* = 3) deposited at controlled *α* and LH-TiO_2 NHs@$CsPbBr_3$/Cs_4PbBr_6 grown on these chiral cores. The chiral cores were deposited at *α* of (a, b) 89°, (c, d) 88.5°, (e, f) 88°, (g, h) 87.5°, (i, j) 87°, and (k, l) 86.5°. (a, c, e, g, i, k) SEM cross-sectional images; (b, d, f, h, j, and l) SEM top-down images. (m) LH-core NHs; (n, o) LH-core@shells. UV–visible spectra: (m-I, n-I) extinction; (m-II, n-II) CD; (m-III, n-III) g_{Ext}. Emission spectra excited at 370 nm: (o-I) PL; (o-II) CPL; (o-III) g_{lum}. Experimental conditions: R_d

= 2.5 Å s^{-1}; P = 100 nm; n = 3; [CsBr] = [$PbBr_2$] = 20 mmol L^{-1}, t_s = 3 h; β = 0.39 (at 86.5°), 0.33 (at 87°), 0.34 (at 87.5°), 0.29 (at 88°), 0.26 (at 88.5°), and 0.20 (at 89°).

According to the suggestion, we have revised the manuscript (**see Line 15, Pg. 15**) and highlighted them in yellow:

Revisions made in the manuscript

*First of all, GLAD was performed at α varying in a range of 86.5°–89°, and the fabrication of TiO_2 NHs was optimized at α = 87° (**Supplementary Fig. 12**). The NHs could hardly be formed at α > 88.5°. α < 87° made the NH arrays too close-packed to facilitate the conformal shell-coating, while the loose-packed NH arrays deposited at α > 87° exhibited weak PL and CPL emission (**Supplementary Fig. 12n, o**).*

ii) In line 1 of page 12, the suppression mechanisms of the chiroptical activities of the thicker shell of the CsPbBr3/Cs4PbBr6 surrounding the TiO2 core are still uncertain even though the authors referred refs 68-70. The authors should describe the details of the reasons why the change of dielectric permittivity of the CsPbBr3/Cs4PbBr6 resulted in the weakening their chiroptical activities.

Response: We thank the reviewer for offering valuable questions and suggestions.

As discussed in Refs. 68–70 (the revised version: Refs. 67-69.), the chiroptical response of plasmonic or dielectric chiral nanostructures is governed not only by their morphological chirality, but also by the local distribution of electromagnetic field and optical chirality in their surrounding medium. Increasing the thickness of the $CsPbBr_3$/Cs_4PbBr_6 shells leads to a pronounced change in the local dielectric environment, which modifies both the amplitude

and spatial distribution of the near fields in the vicinity of TiO_2 NHs.

In particular, the perovskite shells possess a higher effective dielectric permittivity than that of the air (the original medium of TiO_2 NHs), thus reducing the electromagnetic field gradients in the vicinity of chiral cores. According to the framework of optical chirality (Ref. [69] *Phys. Rev. Lett.*, 2010, **104**, 163901), such change directly suppresses the local optical chirality density, which is responsible for the enhancement of chiroptical interactions. As a result, the electric–magnetic dipole interference underlying CD are weakened.

This effect has been experimentally observed in some related systems, where the conformal coating or surface grafting with dielectric materials leads to reducing CD intensity by damping chiral near-field enhancement and mode coupling (Refs. [67] *Small*, 2016, **12**, 6698-6702, [68] *Phys. Rev. X*, 2012, **2**, 031010). In our system, the $CsPbBr_3/Cs_4PbBr_6$ shell plays a similar role, effectively screening the chiral electromagnetic fields generated by the TiO_2 NHs, thereby resulting in the suppression of chiroptical activity.

According to the suggestion, we have revised the manuscript (**see Line 2-7, Pg. 12**) and highlighted them in yellow.

Revisions made in the manuscript

The chiroptical activity of TiO_2 NHs is governed not only by their helicity-associated morphological chirality, but also by the local electromagnetic field and optical chirality in the vicinity of TiO_2 NHs.[69] Compared to the surrounding air medium of the chiral cores, the shell coating leads to increasing the medium dielectric permittivity, reducing the electromagnetic field gradients near the chiral cores, suppressing the local density of optical chirality, and thus decreasing the chiroptical activity of TiO_2 NHs.[67, 68]

(4) Are there any flaws in the data analysis, interpretation and conclusions? In line 17 of page 13, the word of “coin” is correct? Is it coil?

Response: We thank the reviewer for careful reading and correction. Yes, it should be “coil”, as revised in **Line 23, on Pg. 13.** In addition, we have gone through the manuscript to revise the typos and grammatical errors.

(5) Is the methodology sound?

Yes

(6) Is there enough detail provided in the methods for the work to be reproduced?

Yes

Response: We appreciate the reviewer’s positive comments.

(7) Additional comments: The authors should recheck the list of references. For example, refs 31 and 33 are the same. Refs 25 and 71 are also the same.

Response: We thank the reviewer for careful checking and correction. Accordingly, we have revised the reference list in the manuscript.

Reviewer #2

This manuscript reports an artificial intelligence–assisted strategy, termed OptiCPL, for optimizing circularly polarized luminescence (CPL) in chiral core–shell nanostructures based on GLAD-fabricated TiO_2 nanohelices with tunable pitches. These morphochiral helices act as templates that transfer their handedness to conformally coated perovskite shells ($CsPbBr_3$/Cs_4PbBr_6), producing green CPL emitters with manually optimized g_lum values up to 0.20. To overcome the limitations of conventional trial-and-error optimization, the authors introduce a reverse-design framework that integrates multimodal data—including SEM derived morphological features and spectroscopic responses. OptiCPL predicts synthesis conditions that maximize the luminescence dissymmetry factor. Guided by this approach, |g_lum| is enhanced by 75% to 0.35 in inorganic systems, and further boosted from 0.50 to 0.87 in polymer emitters, demonstrating strong generality and transferability.

The manuscript reports several noteworthy advances:

(a) record enhancement of CPL signals using an AI-guided strategy;

(b) identification of pitch-dependent resonant behavior governing CPL amplification;

(c) simultaneous learning from SEM images and spectroscopic data within a unified framework; and

(d) a conceptual shift from trial-and-error optimization to property-driven inverse design.

The work addresses a major bottleneck in CPL materials research—navigating complex synthetic parameter spaces to optimize chiroptical performance. By

linking target CPL spectra directly to synthesis parameters, the study establishes a powerful materials informatics approach aligned with the physics of chiral photonic nanostructures, making a significant contribution at the intersection of chiral photonics and AI-assisted materials design.

Response: We sincerely appreciate the reviewer's positive comments to support this work.

Despite these strengths, several aspects could be clarified or strengthened. First, the framework is formulated specifically for CPL optimization. Although CD spectra are included as auxiliary inputs, the manuscript does not discuss how OptiCPL could be adapted to directly design or optimize CD responses, which would broaden its impact in chiroptical materials research.

Response: We are grateful for this constructive suggestion. We agree that extending OptiCPL beyond CPL optimization to directly target **CD** could broaden the methodological impact on the development of chiroptical materials. At the same time, we would like to clarify why the original framework was formulated with respect to CPL and g_{lum} as the primary objective. From the perspectives of emissive applications (Such as CP-LED (Ref. [R1] *Adv. Mater.* 2025, **37**, 2413669), Bioimaging (Ref. [R2] *Chem. Sci.* 2019, **10**, 4163–4168), and anticounterfeiting (Ref. [R3] *ACS Appl. Mater. Interfaces.* 2022, **14**, 30362–30370), g_{lum} is a key figure of merit, as it reflects the dissymmetry of the emitted circularly polarized light. One should notice that, for general CPL systems, CD (absorption chirality) and CPL (emission chirality) are not necessarily positively correlated, as they may be governed by different electronic/photonic processes and coupling factors. For example, the chirality can be generally imposed on two-dimensional hybrid organic–inorganic perovskites via incorporating chiral

amines at the A site. This modification perturbs the electronic structure and induces pronounced chiroptical responses, with CD signals typically reaching ~100 mdeg (Ref. [R4] *J. Am. Chem. Soc.* 2020, **142**, 9, 4206–4212). However, strong chiroptical activity in absorption is not necessarily translated into efficient CPL emission. The relationship depends on the relative orientation of electric and magnetic transition dipole moments, as well as the balance between radiative and non-radiative decay pathway parameters that are not readily predicted without detailed first-principles calculations. (Ref. [R5] *Adv. Mater.* 2023, **35**, 2108431) Therefore, optimizing CD alone does not guarantee improved CPL, which motivates our focus on directly optimizing $g_{lum.}$ In addition, the CD signals of our inorganic core@shell system is already very strong, so CD optimization was not the primary motivation of this work. We mainly focused on the optimization of CPL performance (i.e., g_{lum}), shedding light on the emissive applications.

Nevertheless, to address the reviewer's concern, we gave an attempt on re-parameterizing the inverse-design stage to directly forecast CD. In this **CD-adapted implementation ("OptiCD")**, the role of the target in the inverse design is swapped such that a **prescribed CD target** is used to reconstruct the corresponding spectral latent representation, and the model then predicts a feasible combination of synthesis parameters under the same multimodal encoders and prediction module. As an example, we prescribed a targeted CD = 2000 mdeg, whereby **OptiCD** predicted a specific set of synthesis parameters (i.e., α = 87°, R_d = 0.15 nm s^{-1}, β = 0.33, P = 403.9 nm, n = 0.96, [CsBr] = 9.5 mmol L^{-1}, [$PbBr_2$] = 10.8 mmol L^{-1}, and t_s = 4.7 h). Accordingly, we fabricate the chiral core@shell nano-luminophores to obtain CD = ~2500 mdeg (**Supplementary Fig. 39f, red spectrum**), which is in well agreement with the

prediction target. This OptiCD result clearly demonstrates that OptiCPL can also be repurposed to predict **CD** by redefining the target and retraining the inverse-design objective accordingly. These results and a corresponding discussion have been added to the revised manuscript and Supplementary Information.

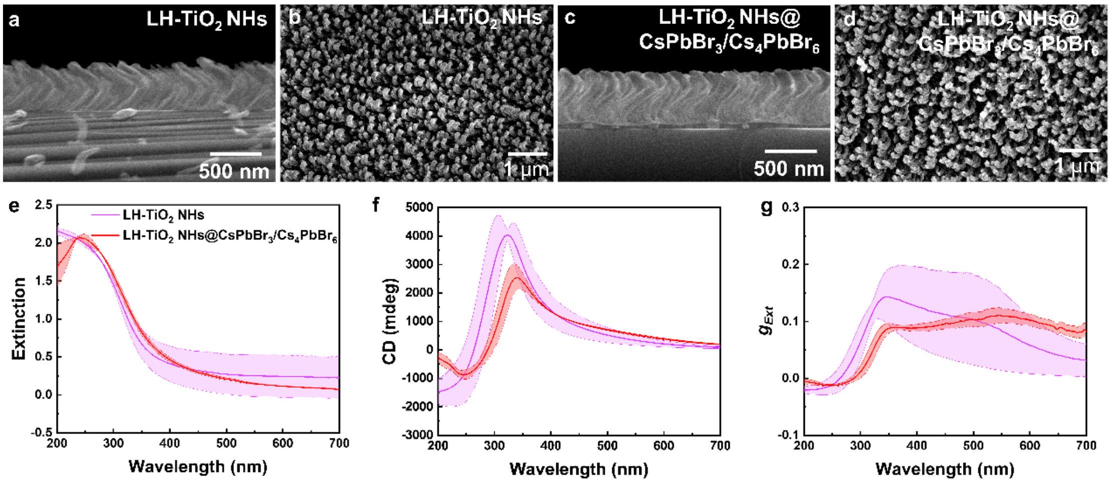

Supplementary Figure 39| Structural and optical characterization of LH-TiO_2 NHs and LH-TiO_2 NHs@$CsPbBr_3$/Cs_4PbBr_6. LH-TiO_2 NHs@$CsPbBr_3$/Cs_4PbBr_6 fabricated under the following experimental conditions that were predicted by OptiCD, to obtain CD = 2000 mdeg for the LH-core@shells: α = 87°, R_d = 0.15 nm s^{-1}, β = 0.33, P = 403.9 nm, n = 0.96, [CsBr] = 9.5 mmol L^{-1}, [$PbBr_2$] = 10.8 mmol L^{-1}, and t_s = 4.7 h. SEM (a, c) cross-sectional and (b, d) top-view images: (a,b) LH-TiO_2 NHs; (c,d) LH-TiO_2 NHs@$CsPbBr_3$/Cs_4PbBr_6. UV–visible spectra of LH-TiO_2 NHs (pink) and LH-TiO_2 NHs@$CsPbBr_3$/Cs_4PbBr_6 (red): (e) extinction; (f) CD; (g) g_{Ext}. Solid lines: mean values; shaded areas: standard deviations; statistically evaluated from multiple measurements.

According to the suggestion, we have revised the manuscript (**see Line 3-11, Pg. 25**) and highlighted them in yellow.

Revisions made in the manuscript

Beyond CPL optimization, the same modular formulation can be readily adapted to the inverse design for optimizing CD signals. By redefining the target

*property in the inverse-design stage from g_{lum} to a prescribed CD spectral profile, the framework called "OptiCD" can propose feasible synthesis parameter combinations for achieving desired CD features. As demonstrated in the proof-of-concept study in **Supplementary Fig. 39**, OptiCD successfully generated the synthesis conditions targeting a specified CD spectrum, and the experimental validation yielded a CD response in considerable agreement with the target. This extension illustrates that OptiCPL is not limited to CPL optimization but can be potentially generalized to other chiroptical design objectives.*

Second, while the model successfully captures pitch dependent resonant behavior, it is not clear which specific electromagnetic quantities the model is using. A clearer connection between learned variables and underlying electromagnetic modes or structural descriptors would improve physical interpretability.

Response: We appreciate the reviewer's valuable comment. Providing a clearer description of electromagnetic information is certainly beneficial. However, quantitative CPL modeling and its direct simulation remain challenging, primarily due to two practical limitations.

First, as a principle-based paradigm, a quantitively predictive theory for CPL in heterogeneous emitters, especially when combined with template-based assembling, has yet to be established. In particular, accurately correlating the measured g_{lum} with the underlying electromagnetic response or structural descriptors is non-trivial, especially when maintaining predictive accuracy across realistic structural disorder and material inhomogeneity (Ref. [R6] *ChemPhotoChem*, 2022, **6**, e202100154; Ref [R7] *Matter*, 2022, **5**, 837-

875; Ref. [R8] *arXiv preprint arXiv*: 2412.13513, 2024).

Second, even if a refined physical theory becomes available in the future, high-fidelity electromagnetic simulations of fabricated core@shell nanostructures would remain difficult. This is due to the polycrystalline nature of the shells, which is composed of grains in a typical size of ~10 nm separated by abundant amorphous grain boundaries, and the corresponding non-uniform dielectric functions. Simplified models can hardly capture such complex experimental microstructures (Ref. [R9] *Appl. Phys. Rev.* 2024, **11**, 011308, and Ref. [R10] *ACS Photonics,* 2022, **9**, 3196-3214). These factors currently hinder precise simulation and principle-based design of g_{lum} for heterogenous CPL-emission materials.

In contrast, OptiCPL is designed to learn directly from experimentally measured far-field electromagnetic observables, that inherently encode resonant and chiroptical signatures. Specifically, extinction and CD spectra are used as physically meaningful proxy descriptors for the chiral cores, while g_{lum} serves as the design target. By avoiding explicitly solving electromagnetic eigenmodes or near-field distributions, the OptiCPL captures the underlying structure–property relationships that govern the measurable chiroptical proxies and in turn CPL performances.

As a footnote of this interpretation, a statistical association was observed between the electromagnetic proxy ($g_{Ext,\ NHs}$ derived from CD and extinction measurements) and the final CPL output ($|g_{lum}|$) in **Supplementary Fig. 26**. Here, the $|g_{lum}|$ exhibits a statistically significant monotonic dependence on $g_{(ext,NHs)}$ (Pearson $r = 0.555$, Spearman $\rho = 0.419$, distance correlation $=$ 0.452; trends supported by both LOWESS and piecewise linear fitting). This provides a quantitative link between a chiroptical proxy ($g_{(ext,NHs)}$) derived from

far-field measurements and experimentally achieved CPL performance ($|g_{lum}|$), confirming that the model leverages physically meaningful chiroptical signatures.

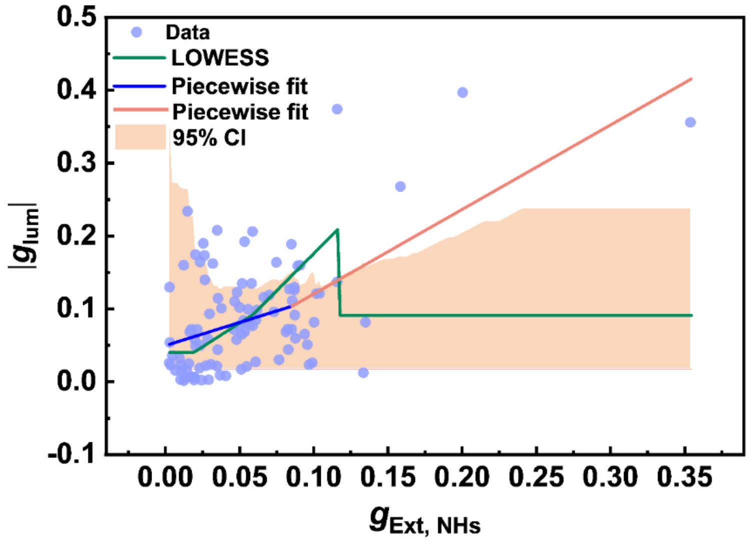

Supplementary Figure 26| Correlation and non-linear analysis between $|g_{lum}|$ and $g_{Ext,NHs}$ of TiO_2 NHs@$CsPbBr_3$/Cs_4PbBr_6. The dark-blue line and orange line represent the piecewise-linear regression, where the optimal breakpoint was selected by minimizing the residual sum of squares within the interior range of $g_{Ext,NHs}$; the two segments remain continuous at the junction. The green line corresponds to the LOWESS non-parametric smoothing (span = 0.3), and the shaded orange shaded region shows its 95% bootstrap confidence interval (CI) (1,000 resamples). Both Pearson ($r = 0.555$, $p < 10^{-3}$) and Spearman ($\rho = 0.419$, $p < 10^{-3}$) tests indicate a significant positive association, further supported by a distance-correlation coefficient of 0.452 ($p \approx 5.0 \times 10^{-4}$). The fitted curves collectively reveal an overall near-linear, monotonic positive dependence, with a slight attenuation of slope at intermediate $g_{Ext,NHs}$ values. The Pearson correlation coefficient (r) denotes the strength and direction of a linear relationship between two continuous variables, ranging from -1 (perfect negative) to +1 (perfect positive), and $r = 0$ indicates no linear relationship. p represents the statistical significance, suggesting the chance of observing your results (or more extreme ones) if the null hypothesis (no real effect) were true, which helps us to decide if an experiment's findings are statistically significant. A smaller p values (e.g., <0.05) suggesting stronger evidence against the null hypothesis and towards a real effect. The Spearman rank correlation coefficient (ρ) denotes the strength and direction of a monotonic relationship (not necessarily linear) between two ranked variables,

ranging from -1 (perfect negative) to +1 (perfect positive), with 0 indicating no association.

Finally, to further strengthen interpretability on the morphology modality, the post-hoc attribution analyses (Ref. [R11] *2017 IEEE International Conference on Computer Vision (ICCV)*, 2017, 618-626) were included in the revised manuscript and supplementary information. Applying Grad-CAM to the SEM encoder (a SimCLR-based ResNet-18) reveals that the model predominantly attends to structurally relevant regions, such as pitch textures and coverage regions related to the NHs in cross sectional views, while showing assigning low attribution to background or irrelevant area. These analyses provide a clearer and more intuitive connection between the learned representations in OptiCPL model and the measurable chiroptical spectral proxies, as well as the structural descriptors.

According to the suggestion, we have revised the manuscript (**see Line 25-26, Pg. 21, and Line 1-8, Pg. 22**) and highlighted them in yellow.

Revisions made in the manuscript

*To gain deeper insight into how morphology modality works, post hoc attribution analyses were performed (**Supplementary Fig. 30**). The results show that the morphology modality of OptiCPL consistently highlights physically relevant structural regions, such as pitch textures and nanostructure-coverage areas, while assigning minimal attribution to background or irrelevant areas. This indicates that the model effectively captures the physically plausible structural cues. Moreover, additional tests show that these attribution patterns remain robust under extra Gaussian noises with 0.1–0.5 in normalized intensity, maintaining high correlation and significant spatial overlap of salient regions compared to the unperturbed baseline (**Supplementary Fig. 31**).*

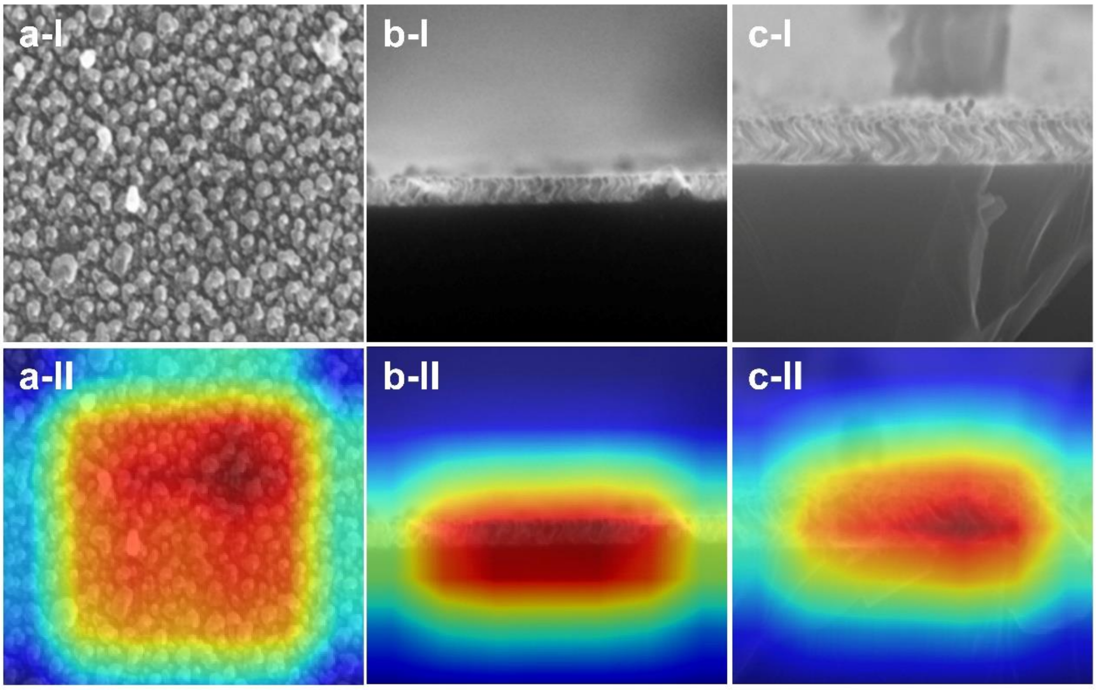

Supplementary Figure 30 | Grad-CAM visualization of morphology modality. (a-I) Representative SEM top-view image and (a-II) Grad-CAM overlay show dominant attribution within structure-bearing regions. (b-I) Representative SEM cross-sectional image and (b-II) Grad-CAM overlay (Morphological Module last convolutional block). High activation regions (red) align with the helical layer/pitch-textured region, rather than the substrate/background. (c-I) Representative SEM tilted image and (c-II) Grad-CAM overlay (Morphological Module last convolutional block). All SEM images were cropped to remove scale bars and annotations prior to training and attribution. Darker red and blue areas indicate greater and less AI attention, respectively.

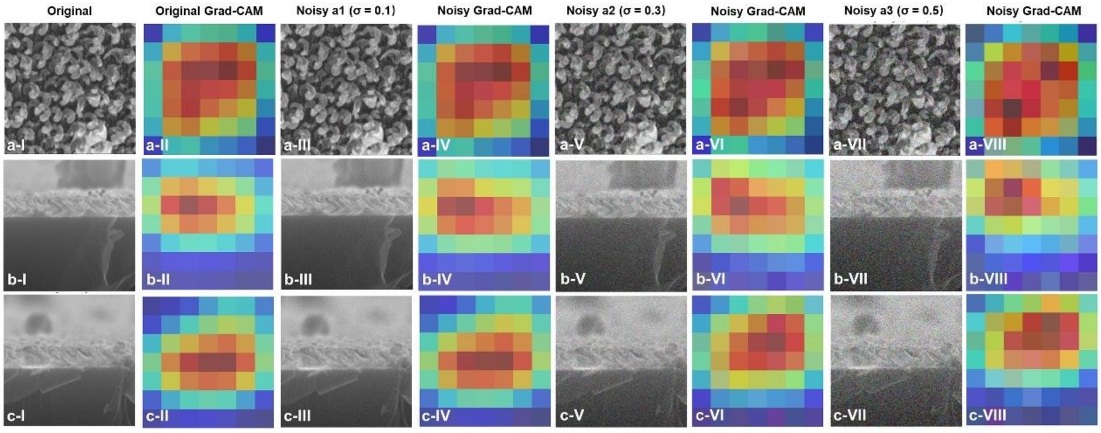

Supplementary Figure 31 | Robustness of Grad-CAM under additive noise perturbations in SEM images. SEM images: (a) top-down viewing; (b) cross-sectional viewing; (c) tilted viewing. (I) Original SEM images. (II) Grad-CAM maps computed from the original SEM images. SEM images via adding Gaussian noise with amplitudes = (III) 0.1, (V) 0.3, (VII) 0.5 (defined on the normalized image-intensity scale), and the corresponding (IV, VI, and VIII) Grad-CAM maps, respectively. It is found that the Grad-CAM patterns remain highly stable under increasing noise levels: the mean Pearson correlation is ~0.992 at 0.1 noise, ~0.933 at 0.3 noise, and remains high (~0.862) at 0.5 noise. These results indicate that the model's attention is not dominated by noise, and the morphology modality remains robust to SEM perturbations within practical ranges.

In addition, uncertainties associated with SEM-based morphology—such as resolution limits, segmentation accuracy, and nanoscale variations in helix width or shell coverage— are not discussed in relation to model robustness.

Response: As suggested by the reviewer, it is important to consider the uncertainties in morphology modality. Accordingly, the morphology modality of OptiCPL is intentionally designed to learn robust morphological representations rather than relying on low-level pixel patterns. Concretely, we implemented an ImageNet-pretrained ResNet-18 and further applied SimCLR pretraining with stochastic image augmentations to improve robustness of morphology-related imaging variability, particularly for random resized cropping, grayscale conversion, random rotation and gaussian blurring (**Supplementary Fig. 27**). In our implementation, each SEM image was randomly resized and cropped to 224 × 224 in pixels, randomly converted to grayscale and rotation, and randomly treated with Gaussian blurring. These transformations were designed to encourage morphology modality for getting robust morphological representations rather than superficial image patterns. All SEM inputs were standardized to a fixed resolution (224 × 224 in pixels) with ImageNet

normalization to avoid confounding variations in pixel sampling.

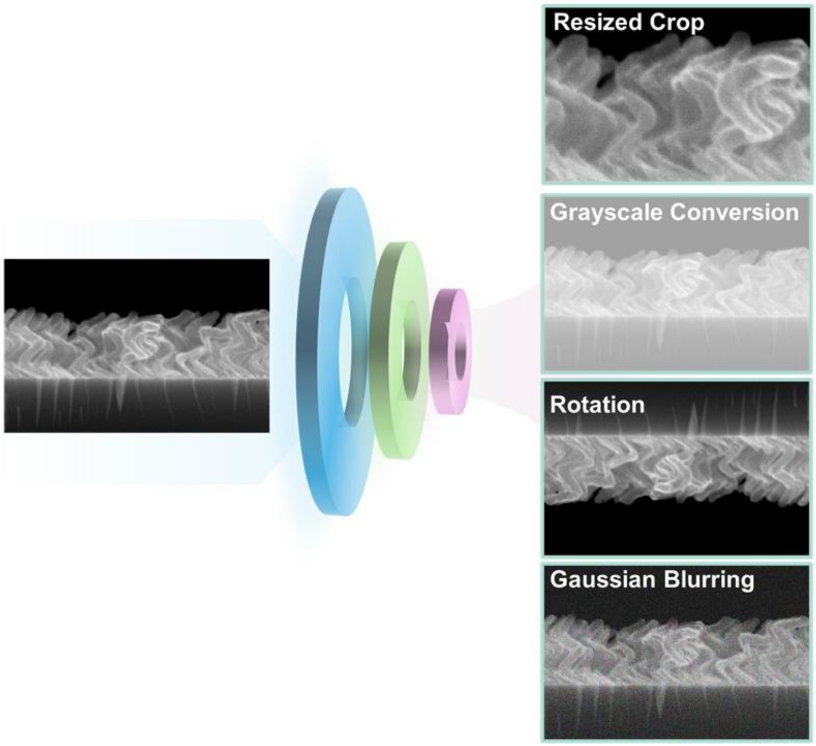

Supplementary Figure 27 | Data augmentation applied in SimCLR. Random resized cropping, grayscale conversion, random rotation and gaussian blurring are implemented for the unsupervised contrastive learning in the morphology modality.

To gain deeper insight into how morphology modality works, post hoc attribution analyses were performed (**Supplementary Fig. 30**). The results show that the morphology modality of OptiCPL consistently highlights physically relevant structural regions, such as pitch textures and nanostructure-coverage areas, while assigning minimal attribution to background or irrelevant areas. Following the suggestion from the reviewer and to provide a more comprehensive engineering assessment, we performed a systematic perturbation test on SEM images and quantified the robustness of the model's morphology attention via Grad-CAM. Specifically, we added Gaussian noise at

a level of 0.1 (10% noise), 0.3 (30% noise), and 0.5 (50% noise) to representative samples for mimicking the low quality images in characterizations, and compared the resulting Grad-CAM maps to the original maps via Pearson correlation of activation patterns (**Supplementary Fig. 31**). The results show that the Grad-CAM patterns remain highly consistent across increasing noise levels, with the mean Pearson correlations of 0.992 at 10% noise, 0.933 at 30% noise, and 0.862 even at 50% noise. These results indicate that the model's attention is not dominated by spurious noise and that the morphology modality remains robust to realistic levels of SEM imaging perturbations.

Based on these findings, we have added a discussion explaining how uncertainties in morphology modality are mitigated by invariance learned through data augmentation in SimCLR pretraining. This empirically verified stability under controlled perturbations further strengthens both the robustness and interpretability of the morphology modality in OptiCPL.

According to the suggestion, we have revised the manuscript (and highlighted them in yellow.

From Line 23, Pg. 18 to Line 3, Pg. 19:

*To address the domain gap between natural image features and microscopic textures present in SEM images, we employed a self-supervised contrastive learning strategy (SimCLR) to adapt a ResNet-18 encoder to our dataset. By leveraging contrastive learning with random augmentations including rotation, cropping, and blurring on unlabeled SEM images, the SimCLR model learned transformation-invariant features that effectively capture the nano-morphological characteristics (**Supplementary Fig. 27** and **28**).*

From Line 24, Pg. 21 to Line 7, Pg. 22:

*To gain deeper insight into how morphology modality works, post hoc attribution analyses were performed (**Supplementary Fig. 30**). The results show that the morphology modality of OptiCPL consistently highlights physically relevant structural regions, such as pitch textures and nanostructure-coverage areas, while assigning minimal attribution to background or irrelevant areas. This indicates that the model effectively captures the physically plausible structural cues. Moreover, additional tests show that these attribution patterns remain robust under extra Gaussian noises with 0.1–0.5 in normalized intensity, maintaining high correlation and significant spatial overlap of salient regions compared to the unperturbed baseline (**Supplementary Fig. 31**).*

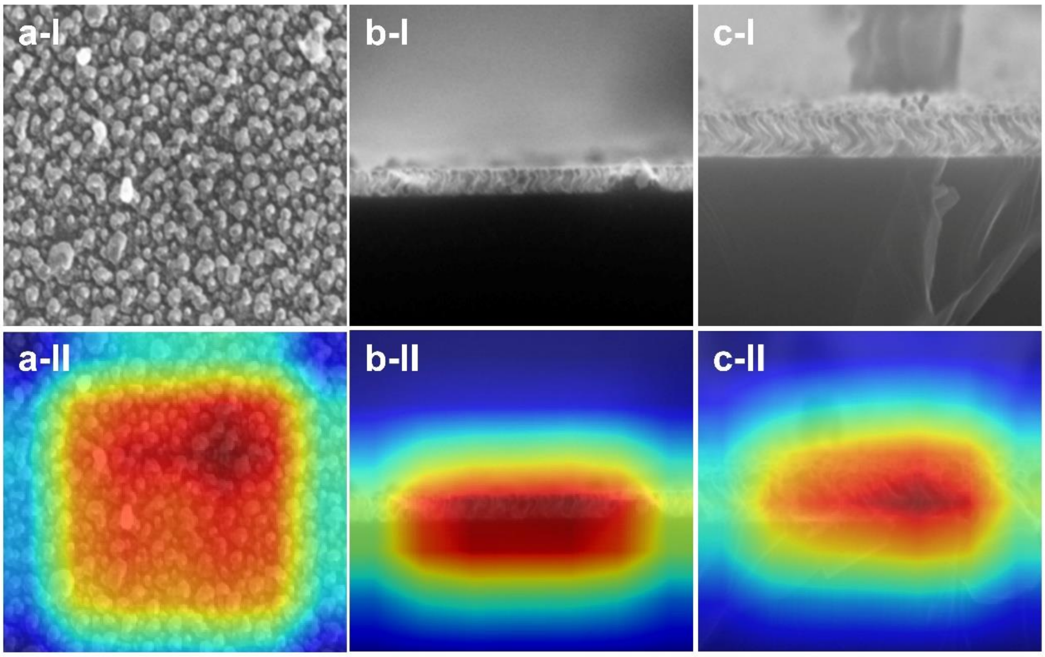

Supplementary Figure 30 | Grad-CAM visualization of morphology modality. (a-I) Representative SEM top-view image and (a-II) Grad-CAM overlay show dominant attribution within structure-bearing regions. (b-I) Representative SEM cross-sectional image and (b-II) Grad-CAM overlay (Morphological Module last convolutional block). High activation regions (red) align with the helical layer/pitch-textured region, rather than the substrate/background. (c-I) Representative SEM tilted image and (c-II) Grad-

CAM overlay (Morphological Module last convolutional block). All SEM images were cropped to remove scale bars and annotations prior to training and attribution. Darker red and blue areas indicate greater and less AI attention, respectively.

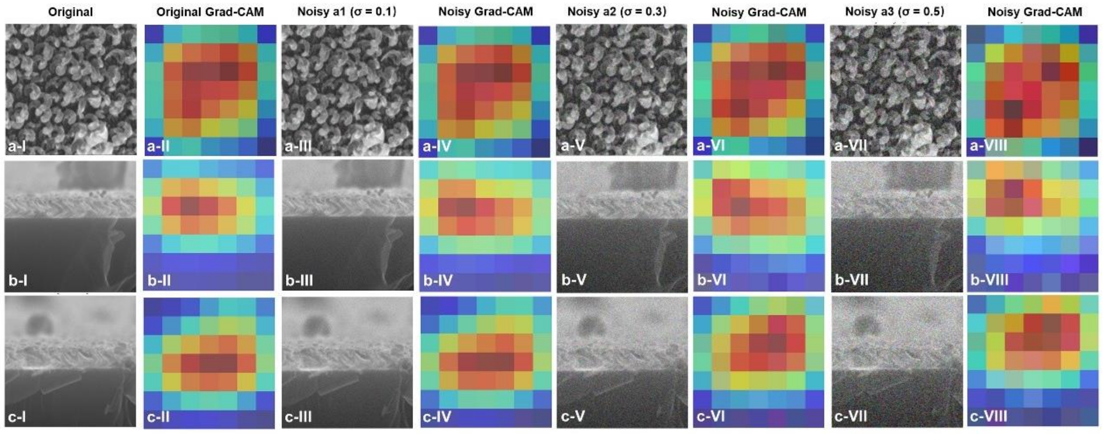

Supplementary Figure 31 | Robustness of Grad-CAM under additive noise perturbations in SEM images. SEM images: (a) top-down viewing; (b) cross-sectional viewing; (c) tilted viewing. (I) Original SEM images. (II) Grad-CAM maps computed from the original SEM images. SEM images via adding Gaussian noise with amplitudes = (III) 0.1, (V) 0.3, (VII) 0.5 (defined on the normalized image-intensity scale), and the corresponding (IV, VI, and VIII) Grad-CAM maps, respectively. It is found that the Grad-CAM patterns remain highly stable under increasing noise levels: the mean Pearson correlation is ~0.992 at 0.1 noise, ~0.933 at 0.3 noise, and remains high (~0.862) at 0.5 noise. These results indicate that the model's attention is not dominated by noise, and the morphology modality remains robust to SEM perturbations within practical ranges.

Finally, the framework is validated only for dielectric helical emitters where CPL arises from photonic mode coupling. Its applicability to systems governed by different mechanisms, such as molecular CPL emitters, remains unexplored and should be clarified.

Response: We sincerely thank the reviewer for the insightful comment. As commented by the reviewer, this work primarily focuses on dielectric nanohelical emitters, where the CPL arises predominantly from the chiral

photonic mode coupling.

That said, the OptiCPL framework is not inherently restricted to the dielectric helical systems at the levels of its learning formulation. In fact, the framework learns a mapping between experimentally measurable inputs, such as synthesis parameters, morphological features, and optical spectrum (extinction/CD/PL/CPL), and targeted chiroptical outputs. Consequently, OptiCPL can be generally extended to diverse CPL emitting systems, as long as these systems provides a consistent dataset that can be constructed while linking controllable variables to the desired chiroptical responses.

A typical example for the OptiCPL's generality is its successful application to F8BT/(*R*/*S*)-5011 system reported in the manuscript. F8BT, as an organic, is an achiral polymer luminophore (chemical structure shown in **Supplementary Fig. 37**), and its CPL can be ascribed to the chirality transfer from chiral inducers (*R/S*)-5011. The successful transfer of OptiCPL from all-inorganic frameworks to this organic chiral system, as two fundamentally distinct regimes, strongly illustrates the framework's transferability.

At the same time, we acknowledge that OptiCPL has not yet been validated for intrinsically chiral molecular emitters, where CPL is substantially governed by molecular electronic coupling in a chiral manner. Extending OptiCPL framework to such molecular systems is conceptually straightforward, which would involve redefining controllable variables and incorporating molecular descriptors along with solution or solid morphology proxies. However, this will require dedicated datasets that adequately capture the relevant degrees of freedom, such as molecular structure, conformational/aggregation states, host–guest interactions, and processing history. At the current stage, we present the F8BT/(*R*/*S*)-5011 case as a meaningful step toward broadening the scope of

OptiCPL, while deliberately avoiding overclaiming its universal applicability.

Accordingly, we revised the manuscript, as follows (**from Line 14, Pg. 24 to Line 12, Pg. 25**) and highlighted them in yellow.

Revisions made in the manuscript

***Application scope of OptiCPL**. OptiCPL is designed as a modular multimodal inverse-design workflow that jointly leverages the morphological information and experimentally measured optical observables (extinction, CD, PL, and g_{lum}) of CPL materials. Here, the OptiCPL framework is validated on the chiral core@shell CPL system, and its generalizability is demonstrated by the successful transfer from inorganic core@shell nanostructures to organic chiral luminophores (F8BT/(R/S)-5011). The applicability of OptiCPL to broader classes of CPL emitters, however, warrants further exploration. In particular, for intrinsically chiral molecular luminophores, the chiroptical performance is governed by intricate factors such as molecular configurations and local packing motifs, [89] which may not be fully captured by the current morphology-based modality in OptiCPL. Extending the framework to such systems would likely require integrating new data modalities, such as molecular graph representations[90] or conformational fingerprints[91] alongside the development of larger, domain-specialized datasets. While these frontiers remain to be fully explored, the modular architecture of OptiCPL provides a scalable foundation for evolving it into a universal platform for AI-driven CPL material discovery.*

Beyond CPL optimization, the same modular formulation can be readily adapted to the inverse design for optimizing CD signals. By redefining the target property in the inverse-design stage from g_{lum} to a prescribed CD spectral profile, the framework called "OptiCD" can propose feasible synthesis parameter combinations for achieving desired CD features. As demonstrated in

*the proof-of-concept study in **Supplementary Fig. 39**, OptiCD successfully generated the synthesis conditions targeting a specified CD spectrum, and the experimental validation yielded a CD response in considerable agreement with the target. This extension illustrates that OptiCPL is not limited to CPL optimization but can be potentially generalized to other chiroptical design objectives.*

To summarize, the AI-guided optimization demonstrates substantial improvements in g_lum for both inorganic and organic systems, underscoring the effectiveness and generality of the approach. The study therefore represents a significant step forward in data-driven design of chiroptical materials.

Response: We respectfully thank the reviewer's supportive summary and insightful feedback, which have greatly contributed to improve the quality of this manuscript. In response to these comments and suggestions, we have implemented substantive revisions, including: (i) adding a CD-targeted inverse-design demonstration to show that OptiCPL can be readily adapted to design and control CD responses; (ii) strengthening model interpretability with additional analyses and discussion that more explicitly connect learned representations to physical chiroptical proxies; (iii) expanding the robustness evaluation of the SEM modality by discussing morphology uncertainties and providing stability tests; and (iv) clarifying the current scope and limitations of applicability by delineating the mechanisms and material classes validated in this work and outlining what extensions would be required for systems governed by different CPL mechanisms. We believe these revisions will significantly enhance the scientific quality of this work.

Reviewer #3

This manuscript reports an all-inorganic chiral core@shell nano-luminophore system optimized using a multimodal deep learning framework (OptiCPL) to enhance circularly polarized luminescence. The experimental platform is carefully constructed, and the achieved glum values are competitive for all-inorganic CPL materials, with the extension to an organic polymer system representing broader applicability. However, several major issues remain regarding the practical effectiveness of the machine-learning-assisted optimization and the overall rigor of the manuscript presentation. In particular, some claims appear insufficiently supported by the available data, and minor but non-negligible issues in data interpretation and reference handling detract from the clarity and reliability of the study.

Response: We appreciate the reviewers' insightful comments, which have helped us improve our manuscript. Below, we address each comment in detail.

1. The authors state that machine learning is employed to reduce experimental effort and accelerate optimization. However, the OptiCPL model requires extensive inputs, including synthesis parameters, detailed morphological information, and multiple spectral datasets, all of which demand a substantial number of experiments and characterizations. It is therefore unclear whether this approach meaningfully reduces the overall experimental burden compared to careful human-guided optimization. The authors should clarify the practical advantage of OptiCPL in terms of experimental cost and efficiency.

Response: We thank the reviewer for raising this important point regarding the practical cost and efficiency of OptiCPL. We would like to clarify that the input modalities used by OptiCPL, including synthesis parameters, morphology, and

spectral characterizations, are not deliberate measurements introduced solely for deep learning. Instead, they are part of the standard experimental workflow commonly required in CPL materials studies to evaluate structure, optical response, and performance. Therefore, OptiCPL does not impose extra experimental burden at the level of sample's characterization.

Actually, this project initially followed a traditional experimental route. Mr. Haifeng Sun spent over one year to generate preliminary data through manual optimization, but progress stagnated at g_{lum} = 0.2, with months of human-guided screening failing to yield further improvement. The research trajectory shifted upon Mr. Yilun Zhang's PhD enrollment and the introduction of machine learning, leading to the development of the OptiCPL framework based on the existing experimental data as the foundation for model training. This approach circumvented the previous optimization deadlock. Only around 20 additional supplementary syntheses were subsequently conducted to diversify the dataset (bringing the total to 128 experiments), and the complete dataset were finally utilized by OptiCPL to achieve g_{lum} = 0.35. By leveraging existing data and integrating information routinely collected, the OptiCPL model enabled us to prioritize more promising synthesis conditions, thereby improving the efficiency of experiments indeed.

Importantly, the challenge in synthesis condition optimization is not caused by these characterization measurements, but by the high-dimensional, coupled, and continuous nature of the design space, which involves numerous synthesis parameters (8 of them were intensively considered in this study). Manual inspection (**Fig. 3**) can qualitatively localize a promising region, but it does not provide a practical way to reliably identify the joint optimum under strong parameter coupling. After preliminary experiments confirmed the feasibility of

the CPL-generating chore@shell system, the influence of individual parameters on the final outcome remained unclear by manual exploration. Variable-control experiments were therefore applied, and the optimal value was determined through iterative screening.

However, another critical issue arises in this process: once parameters in the later stage are optimized, the previously optimized parameters may no longer remain valid. For example, during pitch optimization, increasing the pitch from 300 nm to 600 nm made the original precursor concentration (4 mmol L^{-1}) insufficient to fully cover the chiral cores (**Supplementary Fig. 16-19**), causing g_{lum} decreased from 0.2 to the order of magnitude of 10^{-2} (**Fig. 3a**). This necessitated re-selection and re-optimization of the precursor concentration. But there is no choice. If the variable control was discarded, even a conservative coarse grid search would require an impractically large number of trials. For example, only three discrete levels per parameter already implies $3^8 = 6561$ candidate conditions.

Hence, although manual optimization is feasible, it heavily relies on the experience and judgment of the experimenter. In such a complex system, even an experienced experimenter could not avoid a large number of ineffective or failed experiments, resulting in substantial time consumption and resource consumption. In this context, constructing a dataset of 128 independent syntheses (with 230 total instances used for model training after including data augmentation, as clarified in the revised Methods and Supplementary Information) represents a comparatively economical investment to enable data-driven navigation of the feasible experimental manifold. Importantly, even failed experiments can contribute valuable information by enriching the dataset for better defining the underlying data manifold, which in turn improves the

efficiency and accuracy of the optimization. The result is significant: OptiCPL optimization led to an experimentally validated improvement from $g_{lum} \approx 0.20$ (best manually identified in the original dataset) to $g_{lum} \approx 0.35$ and from $g_{lum} \approx 0.50$ to $g_{lum} \approx 0.87$ in the F8BT/(*R*/*S*)-5011 luminophores.

To put our dataset size into context, it is worth noting that AI-assisted inverse-design studies, that aim to learn actionable process–property correlations in similar systems with multiple noisy and strongly coupled variables, typically operate with hundreds of experiments. For example, Henke *et al.* reported a benchtop "Synthesizer" for perovskite nanocrystal growth that used 287 experimentally samples to form a dataset, enabling reliable prediction and optimization across the accessible synthesis space (Ref. [R12] *Adv. Mater.* 2026, **38**, e09472). Likewise, Zhou *et al.* combined spectroscopic descriptors, generative ML, and an automated synthesis/characterization platform, constructing a dataset of 462 synthesized and characterized high-entropy catalysts to train and validate their inverse-design workflow.(Ref. [R13] *Nat. Synth.*, 2026,). Compared to these similarly complex works, the number of experiments guided by OptiCPL in this work is relatively modest.

According to the suggestion, we have revised the manuscript and Supplementary Information, and highlighted them in yellow.

Revisions made in the manuscript: **Line 1-2, Pg. 18**

Stage 1: construction of multimodal dataset and fine-tuning pre-trained models. *The dataset of OptiCPL comprises 230 data points (128 independent synthesis and data augmentation;* ***Supplementary information: OptiCPL****), ...*

Revisions made in the supporting information: **Line 2-5, Pg. 2**

Data augmentation. *The dataset of OptiCPL contains 230 datapoints,*

stemming from 128 independent synthesis. Data augmentation involved repeatedly measuring a given sample to obtain optical signals exhibiting a certain degree of deviation, thereby increasing the total size of dataset.

2. The OptiCPL-predicted optimal parameters closely resemble the conditions associated with peak glum values already identified in Figure 3 through manual analysis. This raises the question of whether similar optimization outcomes could be achieved by directly interpreting the experimental trends without a deep learning model. While an optimization framework that operates effectively with truly limited data would be highly valuable, the dataset used here is relatively large for an experimental study. The authors should therefore better justify the added value of OptiCPL over conventional data-driven optimization performed by an experienced experimentalist using the same dataset.

Response: We thank the reviewer for this important concern. We agree that Fig. 3 allows a qualitative localization of the high-performance region in the parameter space. As mentioned above, the key added value of OptiCPL lies not in identifying the general vicinity of the optimum, but in resolving an experimentally actionable optimum in a high-dimensional, coupled design space under feasibility constraints, which is a task that could not be reliably accomplished through manual trend inspection alone.

Fig. 3 primarily reflects one-factor trends. In our system, however, chiroptical performance is governed by a multiple-dimensional coupled design space, in which multiple parameter combinations can produce similar apparent one-factor trends while still producing substantially different outcomes because of parameter interactions, for example, between geometric factors (such as the helical pitch *P* and the number of pitches–*n*) and chemical variables (such as

concentrations and soaking time t_s). As a result, although Fig. 3 indicates a promising region for several parameters (including α, β, Rd, P, n, [CsBr], [$PbBr_2$] and t_s), it does not uniquely specify the joint optimum.

Furthermore, the optimal combination predicted by OptiCPL does not simply correspond to a re-selection of the best condition already presented in the original dataset. For instance, P and t_s in the experimental data were sampled at discrete intervals, (e.g. P = 100, 150, 200, … nm; t_s = 4, 5, 6, ... h). However, OptiCPL could identify 107 nm and t_s = 4.9 h as the optimal condition. Importantly, this optimal condition by OptiCPL was experimentally validated and yielded a significantly better CPL performance (g_{lum}) than the best value achieved in the original dataset, illuminating that OptiCPL can move beyond discrete experimental candidates and pinpoint a more precise optimum in the global design space.

Following the suggestion from the reviewer, to further address the "added value over conventional data-driven optimization using the same dataset", we implemented a benchmark against six widely used baseline ML workflows under identical experimental constraints (**Supplementary Table S3**). Specifically, we evaluated: (i) inverse baselines (RF/XGBoost/SVR) that map a 40-dimensional target g_{lum} vector to the 8 synthesis parameters (the same used for OptiCPL); and (ii) forward baselines (RF/XGBoost/SVR) trained to predict $g_{lum,peak}$ from the 8 parameters, followed by Bayesian optimization to propose candidate conditions. For each method, we experimentally tested the proposed conditions. Although some of the predictions results also located in the "ridge region" in Fig. 3 (RF-inverse and RF), we found that these conventional baselines either proposed non-realizable conditions (e.g. SVR predictions violating the allowed range of n), or yielded substantially lower

experimentally verified g_{lum} (typically 0.08–0.14) than OptiCPL. In contrast, OptiCPL proposes a realizable condition and achieves a markedly higher experimentally validated g_{lum}, surpassing the best manually identified CPL performance from the original search. These results collectively indicate that OptiCPL provides practical value beyond manual trend interpretation.

Finally, we agree that the dataset is considerably large for an experimental study, but relatively speaking, it is merely a tip of the iceberg in the vast data space for such complex system, so this does not diminish the utility of OptiCPL. Instead, the conventional data-driven prediction results demonstrate that with such a sizable dataset, the optimization problem remains non-trivial due to high dimensionality parameter coupling. Even the conventional baseline ML workflows could fail in such circumstance. Importantly, once trained, OptiCPL is intended to reduce subsequent trial-and-error. We have revised the manuscript to clarify this point and to present the above benchmarking analyses explicitly.

Accordingly, we have revised the manuscript (**see Line 13-24, Pg. 25**) and highlighted them in yellow.

Comparison with conventional data-driven methods. *To benchmark OptiCPL against conventional data-driven workflows, six widely used machine learning baseline models were evaluated under the identical experimental constraints, including inverse models (RF/XGBoost[92]/SVR) mapping a target* g_{lum} *spectrum to synthesis parameters, as well as forward models (RF/XGBoost/SVR) predicting* g_{lum} *from the synthesis parameters followed by Bayesian optimization. Experimental validation showed that these conventional approaches either propose non-realizable conditions due to instrument constraints (like SVR) or yield substantially lower verified* g_{lum} *values (in a range*

*of 0.08–0.14). In contrast, OptiCPL generates feasible parameter sets and achieves a markedly higher experimentally validated CPL performance, surpassing the best manually identified points in the original search space (**Supplementary Fig. 40**, and **Supplementary Table 3 and 4**).*

Supplementary Table S3. Experimental parameters predicted by traditional machine learning models.

	α (°)	R_d (nm/s)	β	P (nm)	n	[CsBr] (mmol L^{-1})	[$PbBr_2$] (mmol L^{-1})	t_s (h)
**RF-inverse**	86.983	0.151	0.330	413.322	1.028	7.162	7.162	4.7169
**XGBOOST-inverse**	86.587	0.174	0.344	260.965	1.833	16.654	16.654	4.138
**SVR-inverse**	86.975	0.149	0.329	285.611	1.155	16.654	16.654	4.84
**RF**	86.399	0.125	0.385	350.687	1.03	8.037	8.037	3.79
**XGBOOST**	86.757	0.184	0.362	549.9	1	4	4	5.629
**SVR**	85.746	0.1	0.33	600	1	4.87	4.87	5.64

Supplementary Table 4. Experimental parameters applied to fabricate chiral core@shell nano-luminophores, guided by the diverse traditional AI models.

	α (°)	R_d (nm/s)	β	P (nm)	n	[CsBr] (mmol L^{-1})	[$PbBr_2$] (mmol L^{-1})	t_s (h)
**RF-inverse**	87.0	0.15	0.33	413	1.02	7.2	7.2	4.7
**XGBOOST-inverse**	86.5	0.17	0.34	261	1.83	16.7	16.7	4.1
**SVR-inverse**	87.0	0.15	0.33	286	1.16	8.0	8.0	4.8
**RF**	86.5	0.13	0.39	351	1.03	4.0	4.9	3.8
**XGBOOST**	86.5	0.18	0.36	550	1	4.0	4.0	5.6
**SVR**	~~86.0~~	~~0.1~~	~~0.33~~	~~600~~	~~1~~	~~4.87~~	~~4.87~~	~~5.6~~

The SVR's predicted structure was calculated to have a rotational speed of 0.0198 °/s for its R_r (according to the equation 4 in manuscript). This parameter is far below the instrument's designed minimum rotational speed (0.04 °/s), therefore it could not be achieved.

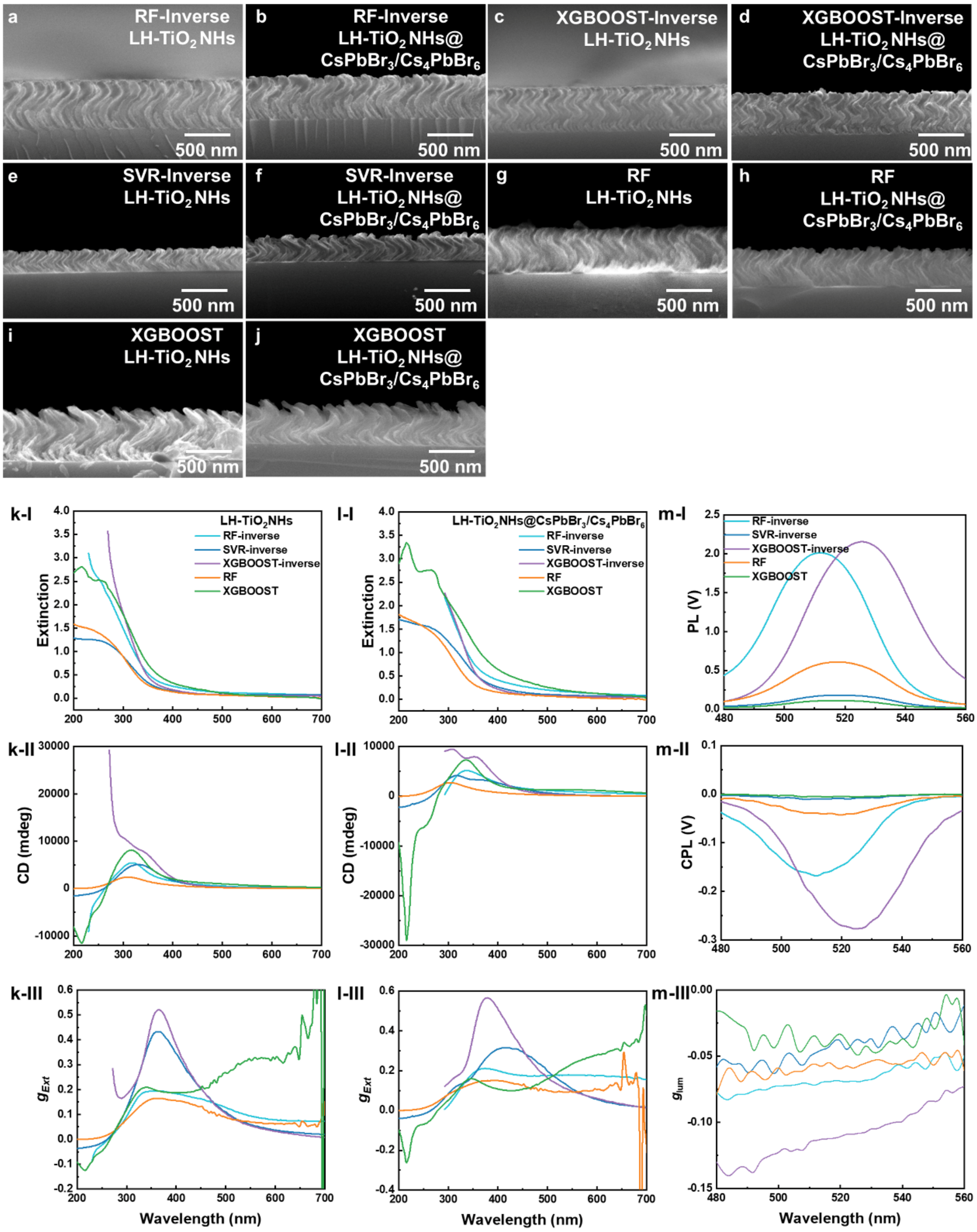

Supplementary Figure 40| Fabrication of LH-TiO_2 NHs and LH-TiO_2 NHs@$CsPbBr_3$/Cs_4PbBr_6 guided with the traditional AI models (including (a, b) RF-inverse, (c, d) XGBOOST-inverse, (e, f) SVR-inverse, (g, h) RF, and (i, j) XGBOOST), with morphological and optical characterizations. (a-j) SEM cross-sectional images: (a, c, e, g, i) LH-cores; (b, d, f, h, j) LH-core@shells. UV–visible spectra: (k-I, l-I) extinction; (k-II, l-II) CD; (k-III, l-III) g_{Ext}. Emission spectra excited at 370 nm: (m-I) PL; (m-II) CPL; (m-III) g_{lum}. The Experimental conditions guided by the diverse traditional AI models and those

applied to generate the chiral core@shell nano-luminophores are summarized in **Supplementary Table 3 and 4**, respectively.

3. The application of OptiCPL to an organic polymer system (F8BT/R,S-5011) is a positive step toward demonstrating generality. However, molecular weight is a key parameter in polymer-based chiroptical systems and is not included as an experimental variable in this study. The authors should clarify whether molecular weight effects were considered and discuss the limitations this omission imposes on the claimed generality of the framework.

Response: We appreciate this insightful comment. As mentioned by the reviewer, the molecular weight (MW) is of course a critical parameter in polymer-based luminophore systems, due to its impact on chain conformation, aggregation, polymer morphology, and consequently the measured chiroptical response. We missed to explicitly report MW information in the previous submission. Here is the detailed information of F8BT and provided in the method section: purchased from Luminescence Technology Corp.; batch: LT-S957-250317001; MW = 35,902 g mol^{-1}; polydispersity index (PDI) = 3.24.

Our purpose of introducing the F8BT/(*R*/*S*)-5011 luminophores is to provide an independent, experimentally accessible testbed to evaluate the *transferability* of the OptiCPL workflow beyond the all-inorganic core@shell nano-luminophores. We selected this system after literature survey, because it (i) shows promising potential for generating CPL, (ii) relies on commercially available materials, and (iii) has a comparatively simple and reproducible processing route, making it suitable as a practical generalization demonstration. Hence, although working as a critical factor, the MW of F8BT was not treated as a tunable design variable in this work. Instead, we intentionally fixed the MW

by using the same product batch throughout the work, so that the OptiCPL-guided optimization was conducted under a given MW $\approx$ 36000.

It is also worth-noting that optimizing the MW of F8BT and its impact on properties (such as absorbance wavelength, photoluminescence properties, glass transition temperature and surface morphology) have been reported extensively in previous works (Ref. [R14] *Polymers*, 2022, **14**, 641, Ref. [R15] *Macromolecules*, 2003, **36**, 2838-2844, and Ref. [R16] *Org. Electron.* 2015, **23**, 87-98). Furthermore, the effect of MW on the addition of F8BT as a luminescent agent to a mixed CPL system of chiral inducible molecules was reported (Ref. [R17] PhD thesis of Claudia Dillmann, Universität zu Köln.) These works have already discussed the importance effect of MW on CPL performance. Herein, we aim to demonstrate the transferability of OptiCPL from all-inorganic nano-luminophores to, e.g., polymer-based organic luminophores. Therefore, we used F8BT with the commercialized MW $\approx$ 36,000 to testify the OptiCPL transferability. At the given MW, we applied OptiCPL to guide the engineering of other experimental conditions (including concentration of F8BT, concentration of *R*/*S*-5011, annealing temperature, annealing duration, spin-coating rate, spin-coating duration), resulting in the significant amplification of g_{lum} from 0.50 to 0.87. This result vividly demonstrates the transferability of the OptiCPL workflow from the all-inorganic to polymer-based luminophores, which is one of the main objectives in this work.

According to the suggestion, we have revised the manuscript and highlighted them in yellow.

Revisions made in the manuscript

Line 2-3, Pg. 24: *In the experiment, F8BT with commercialized molecular weight (MW) $\approx$ 36,000 was utilized.*

Line 1-6, Pg. 27*:* Batch LT-S957-250317001), with MW = 35,902 g mol^{-1} and the reported PDI = 3.24), and S-(13bR)-5,6-Dihydro-5-(trans-4-propylcyclohexyl)-4H-dinaphtho[2,1-f:1',2'-h][1,5]dioxonin (*S*-5011, Macklin, batch: Lot#C15307502, MW = 450.62 g mol^{-1}), R-(13bR)-5,6-Dihydro-5-(trans-4-propylcyclohexyl)-4H-dinaphtho[2,1-f:1',2'-h][1,5]dioxonin (*R*-5011 Bidepharma, batch: BD01413661, MW = 450.61 g mol^{-1}),…

4. References 25 and 71 correspond to the same publication: Liu, P., et al., Optically active perovskite CsPbBr3 nanocrystals helically arranged on inorganic silica nanohelices, Nano Letters 20, 8453–8460 (2020)

Response: We are thankful for the reviewer's careful reading and correction. We have revised the reference list in manuscript.

We sincerely thank Reviewer #3 for the insightful and constructive comments. The reviewer's critical questions regarding the practical effectiveness of OptiCPL, its advantages over manual and conventional data-driven optimization strategies, and the rigor of the organic system validation prompted us to improve the quality of this work. We have significantly enhanced the scientific rigor and completeness of the manuscript by supplementing systematic benchmarking against traditional machine learning baselines, analyzing the improvement efficiency after applying OptiCPL, and providing complete molecular weight information. These revisions not only clarify potential misunderstandings but also highlight the unique advantages of OptiCPL in navigating high-dimensional, complex material design spaces. We sincerely hope that these revisions adequately address the reviewer's concerns and bring the manuscript to a publishable standard.

References

R1. Yao, J., et al. Efficient Spin-Light-Emitting Diodes With Tunable Red to Near-Infrared Emission at Room Temperature. *Adv. Mater.* **37**, 2413669 (2025)

R2. He, T., et al. Water-soluble chiral tetrazine derivatives: towards the application of circularly polarized luminescence from upper-excited states to photodynamic therapy. *Chem. Sci.**10***, 4163 (2019)

R3. Lin, S., et al. Turn-On Mode Circularly Polarized Luminescence in Self-Organized Cholesteric Superstructure for Active Photonic Applications. ACS Appl. Mater. Interfaces 14, 26, 30362–30370 (2022)

R4. Ahn, J., et al. Chiral 2D Organic Inorganic Hybrid Perovskite with Circular Dichroism Tunable Over Wide Wavelength Range. *J. Am. Chem. Soc.* **142**, 9, 4206–4212 (2020)

R5. Jiang, S., et al. Circular Polarized Light Emission in Chiral Inorganic Nanomaterials. *Adv. Mater.* **35**, 2108431 (2023)

R6. Meskers, S. Circular Polarization of Luminescence as a Tool To Study Molecular Dynamical Processes. *ChemPhotoChem* **6**, e202100154 (2022)

R7. Zhang, Y., et al. Circularly polarized luminescence in chiral materials. *Matter* **5**, 837–875 (2022)

R8. Zhao, W., et al. Uncovering the Maximum Chirality in Dielectric Nanostructures. arXiv:2412.13513

R9. Quirk, J., et al. Grain boundaries in polycrystalline materials for energy applications: First principles modeling and electron microscopy. *Appl. Phys. Rev.* **11**, 011308 (2024)

R10. Kar, S. et al. Optical Simulations in Perovskite Devices: A Critical Analysis. *ACS Photonics* **9**, 10, 3196–3214 (2022)

R11. Selvaraju, R., et al. Grad-CAM: Visual Explanations from Deep Networks via Gradient-Based Localization. 2017 IEEE International Conference on Computer Vision (ICCV) 618-626 (2017)

R12. Henke, N., et al. Synthesizer: Chemistry-Aware Machine Learning for Precision Control of Nanocrystal Growth. *Adv. Mater.* **38**, (2026)

R13. Zhou, D., et al. A practical inverse design approach for high-entropy catalysts using generative ai. Nat. Synth. (2026)

R14. Ghasemi, B., et al. Thickness Dependence of Electronic Structure and Optical Properties of F8BT Thin Films. *Polymers* **14(3)**, 641 (2022)

R15. Banach, M., et al. Influence of the Molecular Weight on the Thermotropic Alignment of Thin Liquid Crystalline Polyfluorene Copolymer Films. *Macromolecules* **36**, 8, 2838–2844 (2003)

R16. Abdulla, M. et. al Morphological study of F8BT:PFB thin film blends. *Org. Electron.* **23**, 87-98 (2015)
R17. Claudia, D., Optimisation and Analysis of a Circularly Polarised Organic Light Emitting Diode (CP-OLED). PhD thesis, Universität zu Köln. (2024)

RESPONSE TO THE REVIEWERS' COMMENTS

Reviewer #1

The authors well-addressed all the issues listed in the reviewers' reports, thus allowing to improve the quality of the work. I read this new version very carefully, in particular all new parts, and confirmed that the present revised form now can be ready for publication in the Nature Communications.

Response: We sincerely thank the reviewer for the positive comments that the revised version is now ready for publication in ***Nature Communications***. The reviewer's rigorous and constructive feedback has been invaluable in strengthening this work, and we are deeply grateful for the time and expertise devoted to this review.

Reviewer #2

The reviewer acknowledges the authors' strong efforts in addressing the points raised, which covered mainly four aspects: (i) extending the framework beyond CPL to directly design CD responses; (ii) clarifying the physical interpretability of the aquired results and their link to electromagnetic descriptors; (iii) discussing uncertainties associated with SEM-based morphology and the robustness of the model; and (iv) clarifying the applicability of OptiCPL to CPL systems governed by different mechanisms. The authors provided sound clarifications through important additions, including a new CD-targeted variant (OptiCD) with experimental validation (Supplementary Fig. 39), a statistically significant correlation between the gExt,NHs proxy and |glum| (Supplementary Fig. 26), Grad-CAM attribution maps demonstrating physically meaningful attention on helical pitch-textured regions (Supplementary Fig. 30), and a quantitative noise-perturbation robustness test confirming the stability of the morphology modality (Supplementary Fig. 31). The authors have also added a dedicated "Application scope of OptiCPL" section. As a conclusion, the reviewer supports the publication of the manuscript in its revised format.

Response: We sincerely thank the Reviewer for the careful evaluation of our revised manuscript and for the detailed recognition of our efforts across the four key aspects. The Reviewer's constructive comments were instrumental in guiding these improvements, and we are deeply grateful for the time and expertise devoted to this review. We are truly encouraged by the Reviewer's supportive conclusion for publication.

Reviewer #3

I appreciate the authors' additional explanation regarding the practical motivation for introducing OptiCPL. However, from the manuscript alone, it is still not entirely clear whether the machine learning framework is explicitly addressing the coupled effects among multiple experimental parameters, rather than primarily learning the trends of individually varied parameters. As currently presented, the work may still be read as a well-executed parameter-trend optimization study, and this makes the conceptual distinction from conventional experimental optimization less evident than it could be. I therefore encourage the authors to revise the manuscript to more clearly highlight how multi-parameter coupling is incorporated into the ML framework and why this aspect provides an advantage beyond simple trend analysis. Making this point more explicit would help readers better understand the uniqueness and significance of the work. If this issue is clearly addressed in the revised manuscript, I would be happy to support acceptance.

Response: We sincerely appreciate your valuable comment. Following your comment, now we have further clarified the conceptual distinction between our OptiCPL framework and conventional trend-based optimization and outstood the advantage of OptiCPL by adding two explicit paragraphs in the manuscript. Their core arguments and locations are summarized below:

1. *Highlighting the multi-parameter coupling problem (Lines 367-378, Pg. 16)*

   In this new paragraph, we directly address why the low-dimensional manual optimizations in Fig. 4, although informative, are fundamentally insufficient for identifying the true joint optimum. Using the core@shell system as a concrete example, we explain that the chiroptical response g_{lum} is not governed by any single parameter individually. Instead, it emerges from the coupled effects among template geometry, GLAD conditions, and shell-growth variables. Crucially, we point out that the favorable precursor concentration and soaking time depend on the helix geometry itself (P and n), shell coverage, and chirality transfer. This explicit example vividly shows why simply one-factor scans cannot guarantee the joint optimum. This coupling inherently motivates a deep learning model that learns correlations across the full parameter space rather than isolated one-dimensional trends.

2. *Outstanding how OptiCPL well addresses this coupling (Lines 517-526, Pg. 22, and Line 527, Pg. 23).*

   In the second added paragraph, we clarify the conceptual distinction at the level of the model architecture and workflow. We emphasize that OptiCPL treats each experiment as a multimodal data instance rather than a collection of

independently varied parameters. The regressor is trained to simultaneously predict the multi-dimensional parameter vector, where the correlations and interactions among geometry, shell-growth conditions, and optical responses can be implicitly encoded within the model. In the inverse-design stage, a target g_{lum} spectrum is mapped back to morphology and spectral embeddings and subsquently to a feasible fabrication parameter vector. This means OptiCPL performs joint optimization over the coupled experimental manifold, rather than selecting parameter values independently from one-factor trends. As we now state in the manuscript, this is the indeed factor what allows OptiCPL to bypass the fundamental limitation of manual optimization based on independent parameters in Figure 4.

Revisions made in the manuscript

Line 367-378, Pg. 16:

Although the manual optimization in Fig. 4 identifies several promising parameter regions, these plots represent low-dimensional projections of a substantially higher-dimensional design space. In the present core@shell system, glum is not governed by any single parameter independently, but by coupled effects among template geometry (P, n), GLAD conditions (α, R_d, β), and shell-growth variables ([CsBr], [$PbBr_2$], and t_s). For example, the favorable precursor concentration ([CsBr], [$PbBr_2$]) and soaking time (t_s) depend on the helix geometry, because pitch (P) and turn number (n) affect precursor diffusion, shell coverage, optical resonance, and chirality transfer. Therefore, simply combining the apparent maxima from one-factor scans does not necessarily yield the joint optimum. This coupling makes conventional trend-based optimization inefficient and motivates the use of DL model that learns correlations across the full parameter vector rather than isolated one-dimensional trends.

Line 517- 526, Pg. 22, and Line 527, Pg. 23

This success of OptiCPL comes from its treatment on each experiment as a multimodal data instance, rather than as a collection of independently varied parameters. The regressor is trained to simultaneously predict the multi-dimensional parameter vector, where the correlations and interactions among geometry, shell-growth conditions, and optical responses are implicitly encoded. In the inverse-design stage, a target glum spectrum is mapped back to morphology and spectral embeddings and then to a feasible fabrication parameter vector. Thus, OptiCPL performs joint optimization over the coupled

experimental manifold, rather than selecting parameter values independently from one-factor trends. That makes the OptiCPL easily bypass the limit of manual optimization based on independent parameters in Fig 4.

Finally, we sincerely thank the reviewer again for the constructive and invaluable comments, which are critical for us to improve our manuscript. By following the reviewers' comments, we hope these revisions will be helpful for the publish of the work.
